# Lipid-trap mass spectrometry identifies lipid–protein interactions in cells

**DOI:** 10.1038/s41556-026-01928-6

**Published:** 2026-04-13

**Authors:** Andrea Paquola, Clare E. Benson, Smita Eknath Desale, Cagakan Ozbalci, Elisabeth M. Storck, Stephen J. Terry, Bhagyashree Dasari Rao, Kelechi N. Nwite, Federica Ferrentino, Ulrike S. Eggert

**Affiliations:** 1 Randall Centre for Cell and Molecular Biophysics, King’s College London, London, UK; 2 Department of Chemistry, King’s College London, London, UK; 3 Present Address: Department of Pathology and Cell Biology, Columbia University, New York, NY USA; 4 Present Address: SCIEX UK, Macclesfield, UK; 5 Present Address: Metabolon, London, UK; 6 Present Address: London Centre for Nanotechnology, University College London, London, UK; 7 Present Address: Nikon Imaging Centre at King’s College London, London, UK

**Keywords:** Cell division, Lipids

## Abstract

Cells actively maintain complex lipidomes that encompass thousands of lipids; however, many of the roles of these lipids remain unexplored. Specific interactions between lipids and membrane proteins are a likely reason for lipidome complexity. Here we report the development of a technique, named lipid-trap mass spectrometry (LTMS), to systematically study lipid–protein interactions directly captured from mammalian cells. LTMS uses immunoprecipitation of GFP-tagged proteins expressed in HeLa cells, followed by lipidomic analysis of lipids bound to the GFP-tagged protein. We applied LTMS to cell division to illustrate the technique. We chose this process because membranes regulate their lipid composition as they undergo major changes during cytokinesis, and many cytokinetic proteins, including RACGAP1 and ESCRT-III components CHMP4B and CHMP2A, are membrane-associated. Using LTMS, we found that RACGAP1 and CHMP4B associate with specific lipid species in dividing compared with non-dividing cells. We expand our understanding of lipid diversity during cell division and present a general approach to explore lipid–protein interactions to further our knowledge of the roles of lipids in mammalian cells.

## Abstract (web summary)

Paquola et al. present a technique named lipid-trap mass spectrometry (LTMS) to study lipid–protein interactions directly captured from mammalian cells.

## Main

Lipids are important biological molecules that participate in many essential cellular processes. They primarily reside in the plasma membrane and the membranes of internal organelles. Mammalian cells use evolutionarily conserved pathways to produce thousands of chemically distinct lipids, which requires substantial investments of energy and resources. Phospholipid diversity is defined by variations in polar head groups and hydrophobic acyl chains. In the past, it was thought that lipids’ principal role was to form water-impermeable barriers to compartmentalize different cellular reactions^1^. It is now clear that lipids also have many other functions; for example, they play key signalling roles and are necessary in metabolism and energy storage^1–3^. However, none of these roles explain why cells produce (and therefore presumably require) such large and diverse lipidomes, especially because much of the chemical diversity lies in the hydrophobic fatty acyl chains that are normally buried within lipid bilayers. Emerging literature suggests that specific lipids can interact with membrane proteins^4,5^ and can be required for the functions of these proteins, raising the possibility that specific and regulated lipid–protein interactions could be a reason for the need for lipid diversity. However, so far, systematic studies of lipid–protein interactions in mammalian cells have been limited, both owing to a lack of general understanding of lipid regulation and because there were limited techniques to do so.

It is becoming increasingly clear that lipids are essential for protein activities^5–7^. The lipid environment can affect the binding of agonists and antagonists to GPCRs^7^. Lipids also play key roles in the folding and oligomerization state of membrane-associated proteins^4,6,8^. For example, the addition of cardiolipin to the sugar transporter SemiSWEET can change the equilibrium from monomer to dimer^4^. In addition, experiments in membrane models showed that hydrophobic mismatches due to a difference between the hydrophobic thickness of a membrane and a transmembrane protein segment could induce protein clustering, leading to changes in both their conformation and function^9,10^. The study of lipid–protein interactions and how they modulate protein activity is of particular interest for pharmaceutical development, considering that up to 60% of drug targets are located in the plasma membrane^11^. Different techniques have been developed to investigate lipid–protein interactions in vitro, for example, lipid overlay and lipid pulldown assays. In these assays, lipids are immobilized on a solid support. Proteins are added to the immobilized lipids, and bound proteins are analysed via immunodetection. While this technique has resulted in valuable information, its main downside is that lipid–protein interactions are studied under artificial conditions. Chemically derivatized lipids have also been used to study lipid–protein interactions^12–14^. These lipids can be characterized by a bifunctional unit, such as a photo-activatable group to allow cross-linking with the protein, and an azide or alkyne group that can be ‘clicked’ with an affinity tag, with subsequent identification of protein interactors by mass spectrometry (MS). This powerful approach, which is technically challenging, takes advantage of the specificity of click chemistry to provide informative insights.

Of the techniques developed to study lipid–protein interactions in cellular assays, affinity purification lipidomics using immunoglobulin-based pulldowns is the most straightforward. This method was applied to enzymes involved in the ergosterol biosynthesis pathway in yeasts. A variety of small metabolites, including lanosterol and ergosterol, were isolated^15^. Native protein MS is another promising and successful method^4,5,16,17^. The rationale for the mass spectrometric analysis of native protein assemblies is based on the measurement of small changes in the mass-to-charge ratio (*m*/*z)*, which can indicate the binding of small molecules, including lipids^18^. This technique has already substantially contributed to our understanding of lipid–protein interactions, especially in bacteria. However, we have lacked a technique to systematically study membrane-associated proteins in mammalian cells, yet this is imperative to better understand lipid diversity and shed light on how lipids are dysregulated in disease.

Lipids can associate with membrane proteins in different ways, traditionally classified as non-annular, annular or bulk lipid binding. Non-annular lipids bind to membrane-associated proteins directly, often in specific binding pockets, and have restricted mobility. Annular lipids were traditionally defined as forming a ‘ring’ around the protein and are in dynamic exchange with the surrounding bulk lipids^19^. Although recent research blurs the distinction between annular and bulk lipids^20^, this model provides a tool for conceptualizing how lipids might form dynamic (and possibly functional) nanodomains around membrane-associated proteins. The continuum of associated lipids defines a local nanoenvironment that could, for example, be involved in maintaining the amphipathic milieu necessary for the folded state of a protein^21^, controlling a protein’s localization or defining physical features such as areas of specific curvature. Understanding how membrane-associated proteins interact with their surrounding lipids, both tightly bound and more dynamic lipid domains, is essential to fully investigate their many biological functions. Here, we report the development of a technique that allows the isolation and identification of lipids associated with tagged proteins extracted from their native environment in live cells. We name this technique lipid-trap MS (LTMS) and apply it in a model study of membrane-associated proteins during cytokinesis, the final stage of cell division.

When cells divide, the plasma membrane and all internal organelles, including the lipids they are composed of, are rearranged and distributed among daughter cells^22,23^. The field has a good understanding of which proteins are involved in cell division, how they are regulated and which tasks they perform. However, much less is known about the lipids and the membranes they reside in. We reported that cells regulate their lipid composition with high temporal and spatial precision as they divide. Cells specifically accumulate certain lipids in the midbody, the small structure that forms between cells just before the final cut occurs during cytokinesis^24^. Lipids have also been shown to be involved in several other aspects of cell division^23^. The combination of known important roles, coupled with precise regulation of lipid identity and localization during division, suggested that cytokinesis would be a good model system to test the hypothesis that membrane-associated proteins in cells interact specifically with certain lipids. Applying LTMS to proteins involved in cell division, we show that these proteins interact with specific lipids and that these interactions change with the cell cycle. This work provides insights into cytokinesis and lays the foundation for systematic analyses of interactions between membrane proteins and their associated lipids and subsequent functional studies.

## Results

### Development of LTMS, a technique to identify lipid–protein interactions

**Fig. 1:**
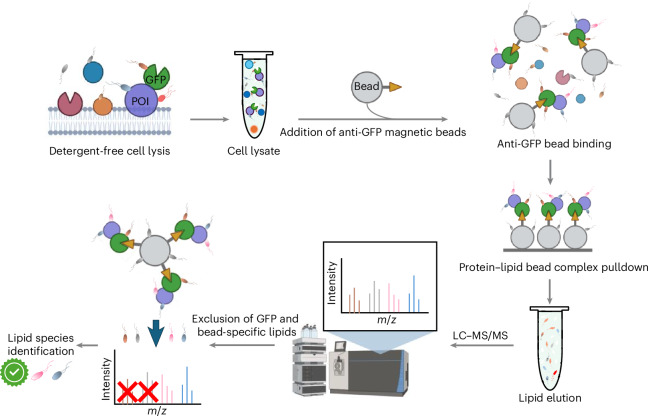
Experimental steps in LTMS. A flowchart illustrating the identification of lipids bound to membrane-associated proteins of interest is shown.

Our approach is to use cells expressing GFP-tagged proteins of interest, lyse the cells under detergent-free conditions to avoid dissociation of lipid-membrane protein bonds, immunoprecipitate the tagged protein, extract the lipids bound to the tagged proteins and finally identify the lipids using lipidomic MS (Fig. 1).

**Fig. 2:**
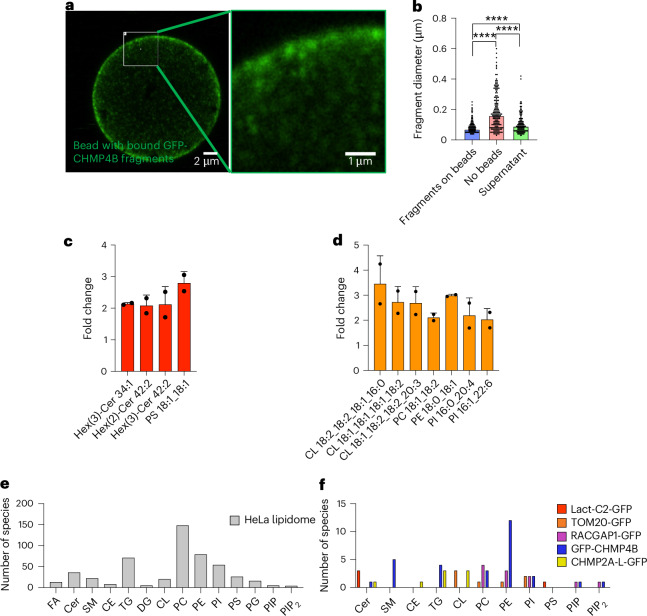
LTMS from GFP-Lact-C2 and TOM20-GFP. **a**, Representative confocal immunofluorescence image of CHMP4B-LTMS lysate derived from HeLa cells stably expressing GFP–CHMP4B (green). Lysates were imaged bound to GFP-Trap beads. Images were acquired using an AXR point-scanning confocal microscope equipped with a super-resolution NSPARC detector (optical resolution 100–120 nm; pixel size 0.05 µm). Left: a single optical section of a bead (scale bar, 2 µm). Right: magnified inset (scale bar, 1 µm). **b**, Quantification of fragment lengths from the lysates shown in Extended Data Fig. 2, performed in the *xy* plane. Data are presented as median ± s.d. (*n* = 3 independent experiments). The approximate median diameters for the ‘fragments on beads’, ‘no beads’ and ‘supernatant’ fractions are 70 ± 41 nm, 160 ± 124 nm and 90 ± 62 nm, respectively. Statistical significance was determined by one-way ANOVA followed by Tukey’s multiple comparisons test (****P < 0.0001; n.s., not significant with a *P* = 0.1211). Note that although smaller structures (<100 nm) may appear visible, reliable physical quantification below this limit is precluded by the optical resolution of the AXR NSPARC system (lateral 100 nm; axial 200 nm; sampling 0.05 µm per pixel). Data are presented as median ± s.d. (*n* = 3). **c**, Bar graph showing the fold increase of all lipids detected with Lact-C2-GFP LTMS compared with MyrPalm-GFP (control). All lipids with a fold increase ≥2 compared with MyrPalm-GFP are shown. Data represent mean value and s.d.; three replicates per experiment were run; *n* = 3 experiments, *P* ≤ 0.05 for all lipids shown. **d**, Bar graph showing the fold increase of the lipids detected in TOM20-GFP LTMS compared with MyrPalm-GFP (control). Lipids with a fold increase ≥1.5 compared with the MyrPalm-GFP control are shown. Data represent mean value and s.d.; three replicates per experiment were run; *n* = 3 experiments, *P* ≤ 0.05 for all lipids shown. **e**, Number of lipid species extracted from the HeLa lipidome. **f**, Combined number of lipid species extracted using LTMS from Lact-C2-GFP, TOM20-GFP, RACGAP1-GFP, GFP-CHMP4B and CHMP2A-L-GFP. FA, fatty acid; Cer, ceramide; CE, cholesteryl ester; DG, diacylglycerol; CL, cardiolipin; PG, phosphatidylglycerol. Source data

**Extended Data Fig. 1:**
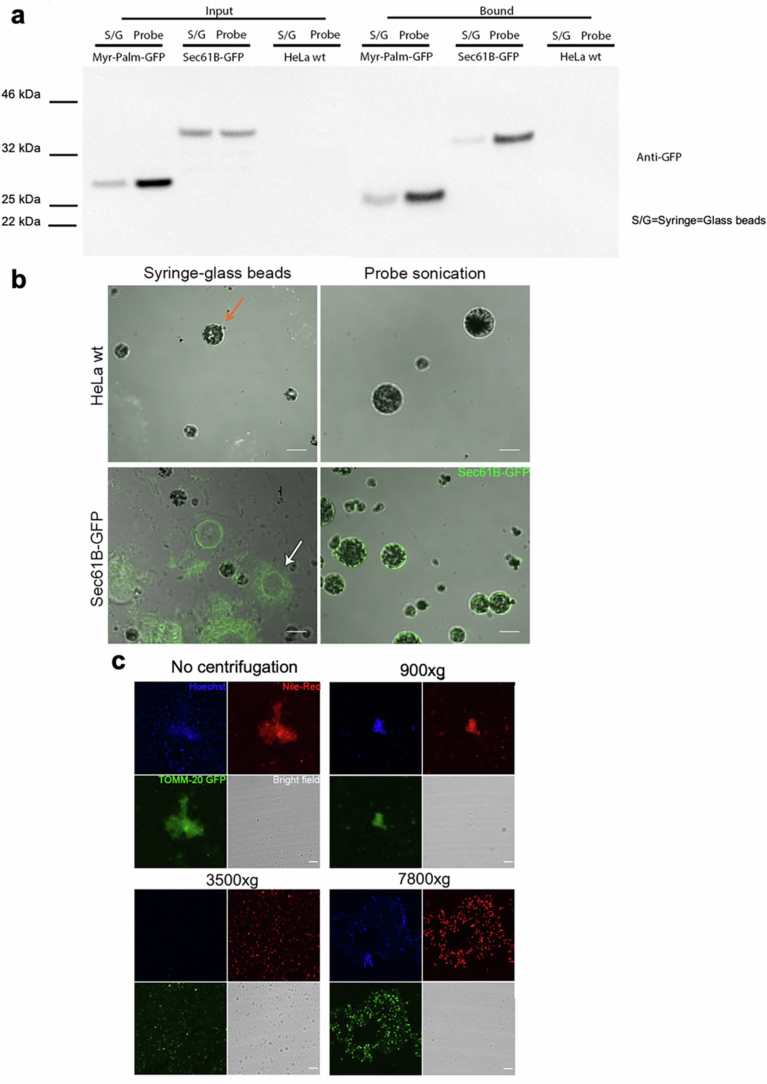
Optimisation and validation of lysis conditions for lipid-trap mass spectrometry. **(a)** Western blot of HeLa wt cells expressing no GFP tag and HeLa cells stably expressing MyrPalm-GFP or SEC61B-GFP, after homogenization with syringe/glass beads (S/G) and probe sonication (Probe), 0.5% of input (left) and 25% of bound fraction to GFP-Trap beads (right) were loaded. N = 2. Unprocessed blots are available in source data. **(b)** Representative confocal images of GFP-Trap beads after equilibration with cell lysates from HeLa wt or HeLa cells stably expressing SEC61B-GFP (green) after homogenization with syringe/glass beads or probe sonication, scale bar=20μm. Orange arrow: GFP-Trap bead. White arrow: intact ER membrane. N = 3. **(c)** Confocal images of cell lysate supernatant from HeLa cells stably expressing TOM20-GFP (green) after probe sonication stained with Hoechst (blue, DNA stain) and Nile-Red (red, neutral lipid stain) after different centrifugation speeds (no centrifugation, 900xg, 35000xg, 7800xg), scale bar=10μm. Source data

**Extended Data Fig. 2:**
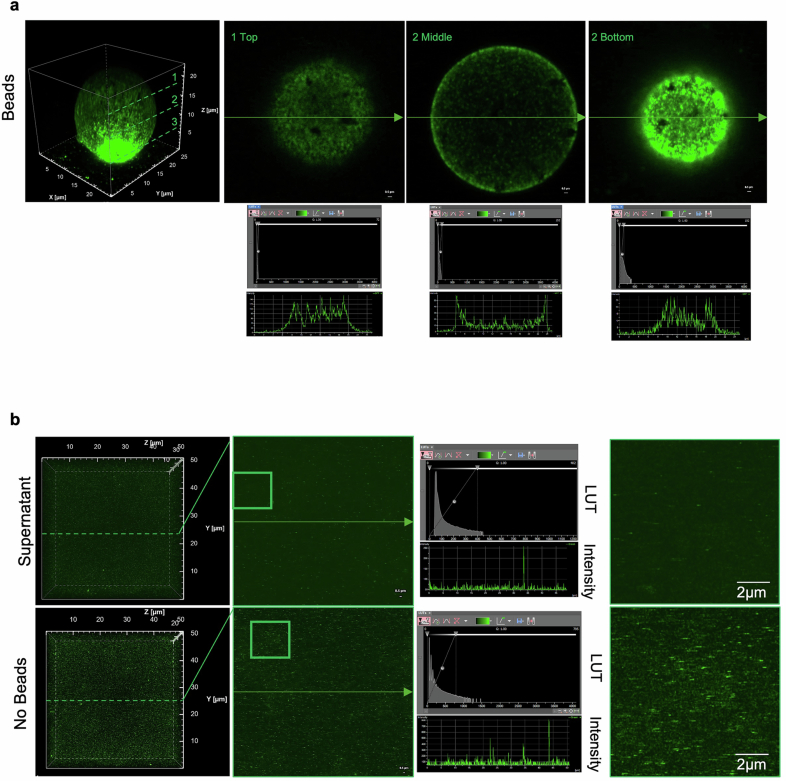
Analysis of LTMS fragment size. **(a)** Representative three-dimensional confocal immunofluorescence images of CHMP4B-LTMS lysates derived from HeLa cells stably expressing GFP–CHMP4B (green). Lysates were imaged on GFP-Trap beads. Images were acquired using an AXR point-scanning confocal microscope equipped with a super-resolution NSPARC detector (optical resolution: 100–120 nm; pixel size: 0.05 µm). The left panel shows a 3D reconstruction of the acquisition, subsequent panels display a single optical sections at the top, middle and bottom of the bead (scale bar=0.5 µm). Below each optical layer are shown the lookup tables (LUT) together with intensity distribution profiles measured along the green arrow. N = 3. **(b)** Representative three-dimensional confocal immunofluorescence images of CHMP4B-LTMS lysates derived from HeLa cells stably expressing GFP–CHMP4B (green). Lysates were imaged before (no beads) and after (supernatant) addition of GFP-Trap beads. Images were acquired using an AXR point-scanning confocal microscope equipped with a super-resolution NSPARC detector (optical resolution: 100–120 nm; pixel size: 0.05 µm). The left panels shows 3D reconstruction of the acquisition, the left middle panels displays a single optical section (scale bar, 0.5 µm) and the right middle panel presents the lookup table (LUT) of the middle layer together with intensity distribution profiles measured along the green arrow. The right panel is a magnification of the green box in the middle panel (scale bar=2 µm). N = 3. The data is quantitated in Fig. 2b.

**Extended Data Fig. 3:**
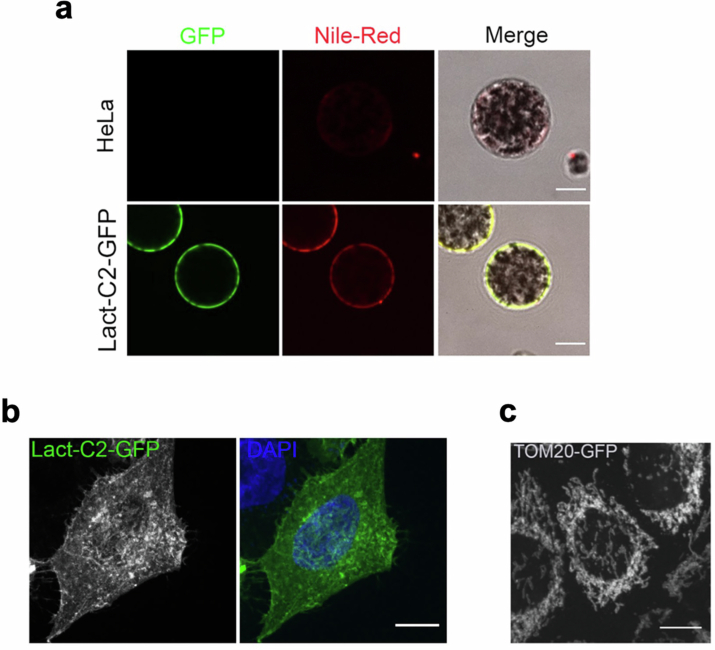
Images of LTMS beads and cell lines used. **(a)** Representative confocal images of GFP-Trap beads after equilibration with cell lysates from HeLa wt and HeLa cells stably expressing Lact-C2-GFP (green) stained with Nile red (red, neutral lipid stain), scale bar=10μm. **(b)** Representative confocal image of HeLa cell stably expressing GFP-LactC2 (green), fixed and stained with DAPI (blue) to visualize DNA, scale bar=10μm. **(c)** Representative confocal image of HeLa cells stably expressing TOM20-GFP (grey), scale bar=10μm.

The first step was to identify a protein tag that is reliable in pulldown experiments and is widely used, which is a prerequisite for a generalizable and modular technique. We chose GFP—there are many well-characterized constructs available where GFP tags do not affect protein function. In addition, the correct localization of the protein of interest in the cell, as well as attachment to beads, can be monitored by fluorescence microscopy. For our initial optimization, we used a HeLa cell line expressing SEC61-GFP, a widely used marker of the endoplasmic reticulum (ER; Extended Data Fig. 1a). GFP-Trap beads, which are magnetic agarose beads coated with a GFP antibody to pull down tagged proteins, are commercially available and commonly used^25,26^. We optimized lysis conditions by comparing sonication or mechanical disruption by vortexing with glass beads and passing the lysate through a syringe with a 27 G 1/2 needle (Extended Data Fig. 1b). A lysate obtained by sonication, followed by centrifugation at 3,500*g* to clear large debris, was added to GFP-Trap beads. The fluorescent GFP signal distributed uniformly over the surface of the magnetic beads (Extended Data Fig. 1c). Nikon Spatial Array Confocal (NSPARC) super-resolution microscopy of lysate from cells expressing the cytokinesis protein GFP-CHMP4B showed GFP-positive membrane fragments in the lysate range from the optical detection limit of ~100 nm up to 500 nm, with a median diameter of 160 ± 124 nm (Fig. 2a,b and Extended Data Fig. 2). Some of the larger fragments are probably incompletely dissociated or aggregated membrane structures that do not appear to bind to beads. Primarily smaller fragments close to the detection limit were captured by the beads (median diameter 70 ± 41 nm), keeping in mind the optical detection limit of 100 nm (Figs. 2a,b). Using Nile Red as a generic lipid label with high affinity for neutral lipids^27^, we showed that GFP-positive beads costained with the lipid dye (Extended Data Fig. 3a). The bead-bound fraction was then taken forward for lipidomic analysis.

After washing and isolation with a magnet, we extracted lipids from these beads with the organic solvent mixture CHCl_3_/MeOH (2:1, v/v)^28^. Lipids were then separated by ultra-high-performance liquid chromatography based on their size and hydrophobicity prior to being subjected to MS. After the initial MS run, we performed statistical analysis to identify species (called features) that were enriched in LTMS pulldowns. We used membrane-localized myristoylated and palmitoylated GFP (MyrPalm-GFP) as a control for non-specific lipid binding (to GFP or beads). All experiments were performed using MyrPalm-GFP and GFP-PoI (protein of interest) in parallel. Any features that were pulled down in similar amounts by controls and PoI were excluded from further analysis, including lipid identification (Fig. 1). Selected features with significant changes compared with control were analysed by tandem MS. LIPID MAPS^29^, MS-DIAL^30^ and MS-FINDER databases were used to match the lipid fragments from tandem MS and identify which lipid species corresponds to a selected feature (Supplementary Notes 1 and 2).

### LTMS identifies known lipid–protein interactions

Before applying LTMS to proteins involved in cell division, we validated this method using known lipid–protein interactions. First, we created a HeLa cell line stably expressing GFP-tagged lactadherin-C2 (Extended Data Fig. 3b). Lactadherin is a peripheral membrane protein involved in different functions, including stabilizing the phospholipid bilayer that surrounds triglyceride globules in breast milk and mediating phagocytosis of dying cells^31^. Lactadherin binds phosphatidylserine (PS) through its C2 domain (Lact-C2), which is used to visualize PS in cellular imaging studies^32,33^. We found that a PS species (PS 18:1_18:1) was indeed pulled down (Fig. 2c, Supplementary Table 1 and Supplementary Notes 1). Several other lipids, glycosylated ceramides, also associated with Lact-C2. Glycosylated ceramides are synthesized in the Golgi, as is a pool of PS, and then both are trafficked to the plasma membrane where glycosylated ceramides are usually found on the outer leaflet of the plasma membrane, whereas PS is found in the inner leaflet. These data provided the promising first hints that, as was our intention, we are isolating regions of lipids or areas of the membrane associated with our protein of interest, rather than only non-annular interactors.

Next, we created a cell line stably expressing GFP-tagged TOM20. TOM20 is located in the outer membrane of mitochondria and is part of the mitochondrial protein translocation machinery (Extended Data Fig. 3c). Applying LTMS, we identified several lipids associated with TOM20, including cardiolipins (Fig. 2d). Cardiolipins are unique to mitochondria, further showing that the lipids pulled down by our technique are specific to the protein of interest. Although cardiolipins are mostly found in the inner membrane of mitochondria, they are required for the function of the TOM complex and specifically the interaction of TOM20 with other components of the complex^34,35^, confirming our finding. We also identified specific phospholipids, including phosphatidylethanolamine (PE), phosphatidylinositol (PI) and phosphatidylcholine (PC) (Fig. 2d, Supplementary Table 1 and Supplementary Note 1), all of which are found in mitochondria^36^. These data further support that we are identifying individual lipids as well as regions of the membrane associated with our tagged proteins.

The lipids identified by LTMS from Lact-C2 and TOM20 were consistent for each protein across several repetitions but different between the two proteins, minimizing the possibility that we are observing non-specific binding. We further confirmed this by comparing the number of lipid species identified from a whole-cell HeLa extract (Supplementary Table 2) with lipids from the LTMS experiments presented in this manuscript (Supplementary Table 1). The lipids from different LTMS experiments varied and were different from the whole HeLa lipidome (Figs. 2e,f). Therefore, our validation experiments show conclusively that LTMS can successfully identify lipids associated with membrane proteins.

### The cytokinesis protein RACGAP1 binds to specific lipids during division

**Fig. 3:**
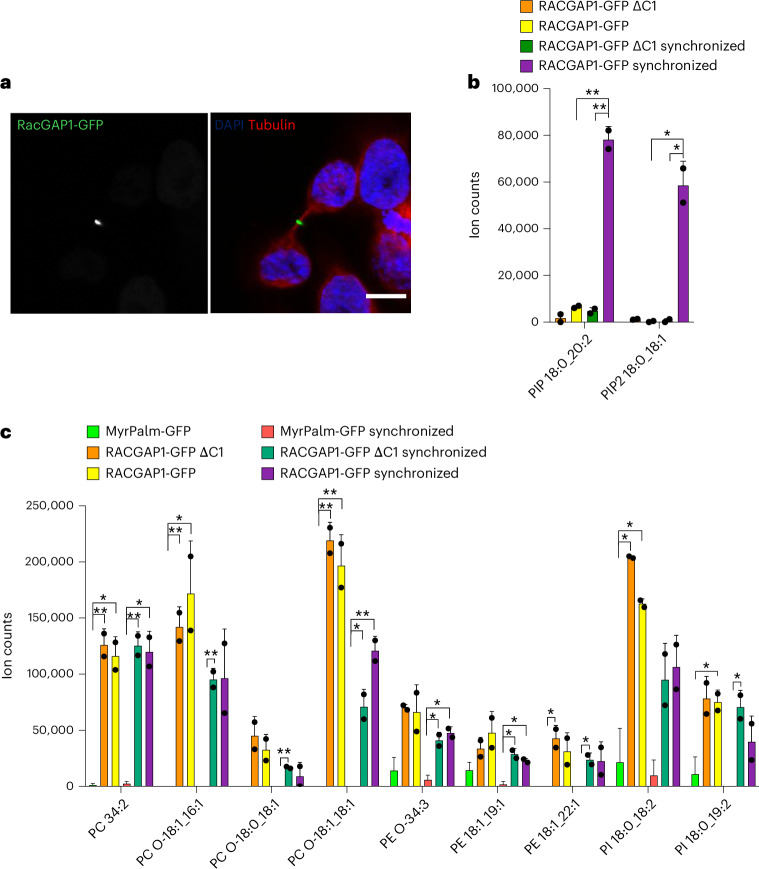
LTMS from RACGAP1-GFP. **a**, Representative confocal image of HeLa cell in cytokinesis stably expressing inducible RACGAP1-GFP (green), induced for 1 h, fixed and stained with DAPI (blue) and anti-tubulin antibody (red) to visualize DNA and microtubules. Scale bar, 10 μm. **b**, Bar graph showing ion counts of all altered PIP species detected after LTMS of RACGAP1-GFP in wild type and C1 deletion, in synchronized or non-synchronized cells. Data represent mean value and s.d.; three replicates per experiment were run; *n* = 2 experiments. **c**, Bar graph showing the ion counts of lipids detected from RACGAP1-GFP and RACGAP1-GFP ΔC1 LTMS experiments in synchronized or non-synchronized samples. All lipids with a fold increase ≥2 compared with MyrPalm-GFP and FDR-adjusted *P* values ≤0.05 are shown. Statistical significance was assessed using multiple two-sided *t*-test based on FDR with the two-stage linear step-up procedure of Benjamini, Krieger and Yekutieli, with *Q* = 5%. *P ≤ 0.05, **P ≤ 0.01. Data represent mean value and s.d.; three replicates per experiment were run; *n* = 2 experiments. Source data

**Extended Data Fig. 4:**
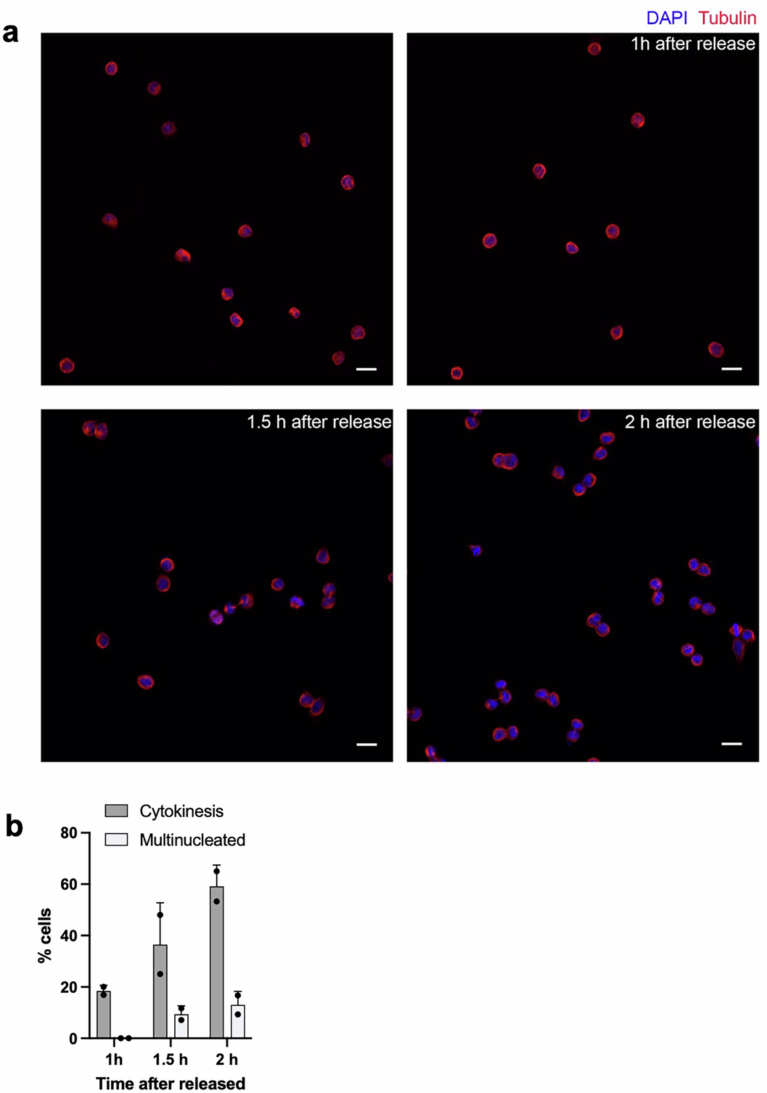
Synchronization of HeLa cells at cytokinesis. **a)** Representative confocal images of HeLa cells fixed and stained with DAPI (blue) and anti-tubulin antibody (red) to visualize DNA and microtubules. Cells were treated with nocodazole (100 ng/mL) for 12 h, washed and imaged 1 h, 1.5 h and 2 h after mitotic shake-off. Note the highest number of cytokinetic cells is 2 h after release. The image was acquired with an A1 confocal inverted microscope using a 40X air lens. Scale bar=20μm. **(b)** Quantification of the percentage of HeLa cells synchronized at cytokinesis at 1 h, 1.5 h and 2 h. Data represent mean value and standard deviation (SD), ~ 60 cells per experiment. N *=* 2 experiments. Source numerical data are available in source data. Source data

**Extended Data Fig. 5:**
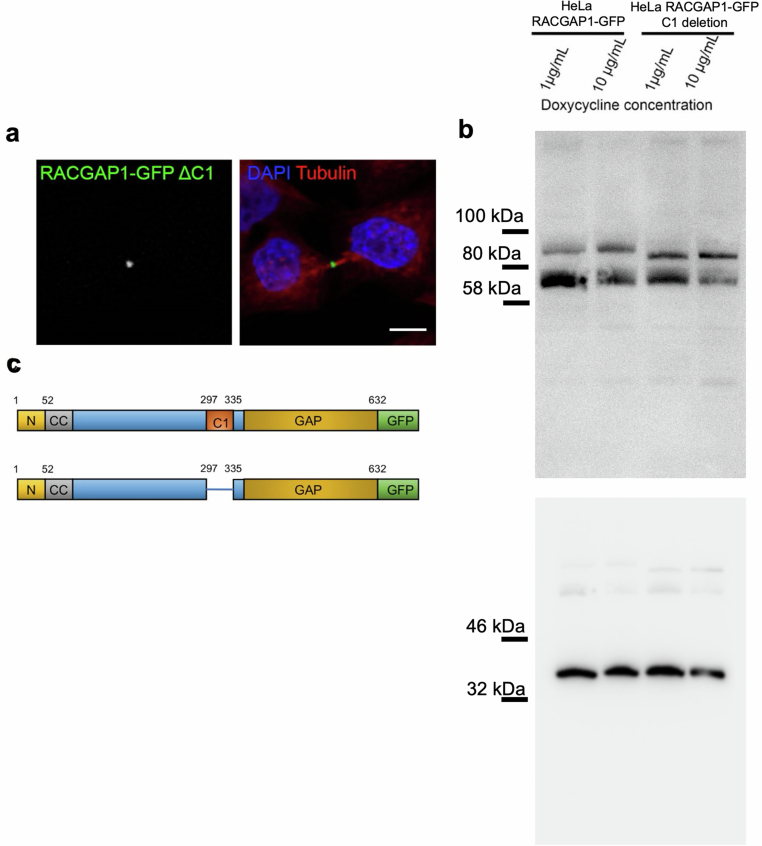
RACGAP1 C1 domain deletion. **(a)** Confocal image of HeLa cell stably expressing RACGAP1-GFP ΔC1 (green) at cytokinesis, fixed and stained with DAPI (blue) and anti-tubulin antibody (red) to visualize DNA and microtubules, respectively scale bar=10μm, N = 3. **(b)** Western blot of HeLa cells stably expressing RACGAP1 and RACGAP1 ΔC1 showing endogenous RACGAP1 (lower band) and transfected RACGAP1-GFP (upper band, black arrow), GAPDH (loading control). The SDS-PAGE gel was run using the total lysate of HeLa cells stably expressing RACGAP1-GFP and RACGAP1 ΔC1- GFP. N = 2. Unprocessed blots are available in source data. **(c)** Cartoon depicting domains of RACGAP1 and its ΔC1 mutant; C1, cysteine-rich domain; CC, coiled-coil; GAP, GTPase-activating domain. Adapted from Lekomtsev et al. Source data

Having established that LTMS can identify lipids known to be associated with membrane proteins, we next investigated proteins involved in cytokinesis, starting with RACGAP1. RACGAP1 links the mitotic spindle to the plasma membrane and is essential for central spindle formation^37^. It is localized at the intercellular bridge during cytokinesis and predominantly in the nucleus in interphase^38^ (Fig. 3a). We chose to focus on this protein because it is essential for cytokinesis and has a known lipid interaction; a domain responsible for this interaction (C1) has been documented^38^. To better understand any cytokinesis-specific interactions, we compared lipids bound to protein extracted from non-synchronized cells with those from cells synchronized at cytokinesis (~65% synchrony; Extended Data Fig. 4a,b). As stable overexpression of RACGAP1 is deleterious to cells, we created stable cell lines expressing inducible full-length RACGAP1-GFP^38^ (Fig. 3a) as well as a mutant lacking the C1 lipid-binding domain^38^ (Extended Data Fig. 5a–c). After expression of RACGAP1-GFP or RACGAP1-ΔC1-GFP was induced for 1 h, we used LTMS to identify lipids associated with these proteins. It was previously shown that the C1 domain of RACGAP1 can interact with PI phosphates (PIPs) PI(4,5)P_2_ and PI(4)P in vitro^38^. As our standard lipidomic MS protocol cannot reliably detect PIPs, we performed a PIP derivatization to investigate whether RACGAP1 can bind to PIPs in vivo. This protocol cannot differentiate between PIP phosphorylation sites, for example, PI(4,5)P_2_ versus PI(3,4)P_2_, as they have the same molecular mass but it can identify the acyl chain configurations within this lipid family, which previous studies were unable to do. We found that only full-length RACGAP1 from cells synchronized at cytokinesis was able to bind PIPs. LTMS from non-synchronized cells or with constructs lacking the PIP-binding C1 domain showed no or small amounts of PIPs (Fig. 3b), confirming previous in vitro findings^38^ and further validating our technique. From our control HeLa extract (prior to LTMS), we were able to identify five PIP and four PI bisphosphate (PIP_2_) species with different acyl chain patterns, with PIP 18:0_20:4 and PIP_2_ 18:0_18:1 being the most abundant species (Supplementary Table 2). Interestingly, however, we could only detect a single PIP_2_ (PIP_2_ 18:0_18:1, which is most abundant in HeLa extract) and a single PIP (PIP 18:0_20:2, which is least abundant in HeLa extract) species bound to RACGAP1 (Fig. 3b and Supplementary Table 1). Although there has been much research on the important signalling roles of PIPs^22,39^, there has been little emphasis on which specific acyl chain configurations might be involved. Our study shows that proteins in live cells distinguish between PIP species with high specificity and therefore presumably use specific PIP species for different purposes.

In addition to analysing PIPs, we also investigated if RACGAP1 and RAGAP1-ΔC1 bound other lipids (Fig. 3c, Supplementary Table 1 and Supplementary Notes 1). Several lipids were pulled down reproducibly but independently of cell cycle state or inclusion of the C1 domain. These data suggest that RACGAP1 can interact with lipids specifically but independently of the C1 domain. This may represent a general membrane attachment state that is then altered by specific interactions with PIPs via the C1 domain during cell division.

### Proteins of the ESCRT-III abscission machinery are associated with specific lipids

**Fig. 4:**
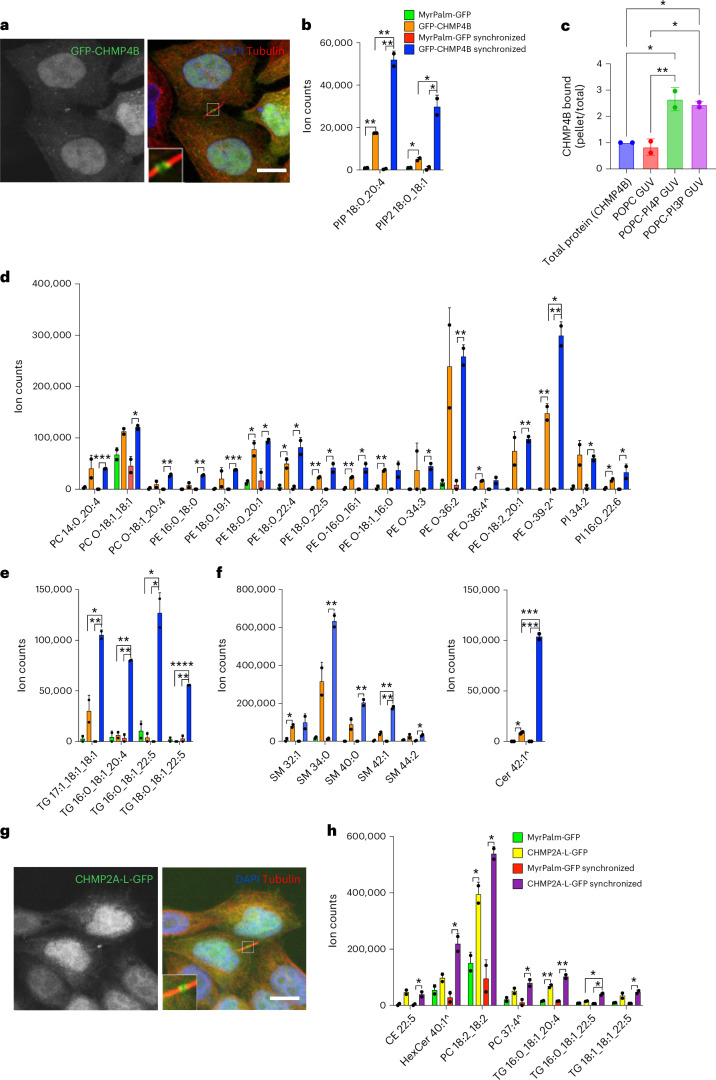
LTMS from GFP-CHMP4B and CHMP2A-L-GFP. **a**, Representative confocal images of HeLa cells stably expressing GFP-CHMP4B (green) fixed and stained with DAPI (blue) and anti-tubulin antibody (red) to visualize DNA and microtubules. Scale bar, 10 μm. **b**, Bar graph showing the abundance of PIP 18:0_20:4 and PIP_2_ 18:0_18:1 detected after pulldown of CHMP4B in synchronized and non-synchronized cells. Data represent mean value and s.d.; three replicates per experiment were run; *n* = 2 experiments. **c**, Quantification of liposome precipitation assay, the values species ratio of bound CHMP4B (pellet/total) with liposome. For the comparison, ordinary one-way ANOVA followed by Tukey’s test for multiple comparison was performed to compare the means; *P < 0.01, **P < 0.005; *n* = 2 experiments. **d**–**f**, Lipid analysis from GFP-CHMP4B-LTMS in non-synchronized and synchronized samples showing phospholipids (**d**), TGs (**e**) and sphingolipids (**f**). Bold: increased in synchronized samples relative to non-synchronized samples. All lipids with a fold increase ≥2 compared with MyrPalm-GFP and FDR-adjusted *P* values ≤0.05 are shown. Statistical significance was assessed using multiple two-sided *t*-test based on FDR with the two-stage linear step-up procedure of Benjamini, Krieger and Yekutieli, with *Q* = 5%. *P ≤ 0.05, **P ≤ 0.01, ***P ≤ 0.001, ****P ≤ 0.0005. Data represent mean value and s.d.; three replicates per experiment were run; *n* = 2 experiments. **g**, Representative confocal images of HeLa cells stably expressing CHMP2A-L-GFP (green) in interphase and cytokinesis stained with DAPI (blue) and tubulin (red) to visualize DNA and microtubules. Scale bar, 10 μm. Inset: zoom with brightness enhancement of the intercellular bridge. **h**, Lipid analysis from CHMP2A-L-GFP in synchronized and non-synchronized samples. Bold: increased in synchronized samples. All lipids with a fold increase ≥2 compared with MyrPalm-GFP and FDR-adjusted *P* values ≤0.05 are shown. Statistical significance was assessed using multiple two-sided *t*-test based on FDR with the two-stage linear step-up procedure of Benjamini, Krieger and Yekutieli, with *Q* = 5%. *P ≤ 0.05, **P ≤ 0.01, ***P ≤ 0.001, ****P ≤ 0.0005. Data represent mean value and s.d.; three replicates per experiment were run; *n* = 2 experiments. Caret represents tentative assignment with only retention time, *m*/*z* and adduct type due to low abundance. Source data

Next, we applied LTMS to two components of the ESCRT-III machinery required for the final cytokinetic membrane abscission: CHMP4B and CHMP2A. CHMP4B and CHMP2A are pivotal in cytokinesis, as their depletion perturbs abscission^40,41^. ESCRT-III components are dynamic and, in addition to an initial pool localized close to the midbody, a second pool, probably responsible for the final abscission, is found at the midbody periphery^42^. The ESCRT-III complex is also essential for other biological functions such as endosomal sorting, viral budding and nuclear envelope sealing^43,44^. Similarly to CHMP4B (Fig. 4a), CHMP2A localizes to the midbody during cytokinesis (Fig. 4f), and, as with CHMP4B, is recruited in all the main ESCRT-III-catalysed processes^45^. Despite acting at a similar timing during cytokinesis and both being necessary for abscission, CHMP4B and CHMP2A appear to have slightly different functions. CHMP4B seems to be the key component for the physical abscission of the intracellular bridge, while CHMP2A is essential for CHMP4B localization and important for the recruitment of VPS4, the ATPase responsible for depolymerization of ESCRT-III^46,47^. We were interested to understand better how ESCRT-III interacts with membranes and if these two components that localize in the same membrane region interact with similar lipids.

In vitro, some experimental evidence suggests that CHMP4B has a higher affinity for membrane invaginations compared with planar membranes and that it binds preferentially to negatively charged membranes^48^. By contrast, other findings show that CHMP4B and other ESCRT-III components do not have an affinity for negatively charged membranes on their own but they may require ESCRT-I and ESCRT-II to coordinate their assembly^49^. However, CHMP2A was shown to bind positively curved membranes and to be required for CHMP4B localization in membrane tube structures^49^. These studies gave us important information about CHMP4B’s and CHMP2A’s preferred membrane conformation, but they were done using cell-free membrane models. Therefore, we decided to explore the nature of these interactions in cells.

**Extended Data Fig. 6:**
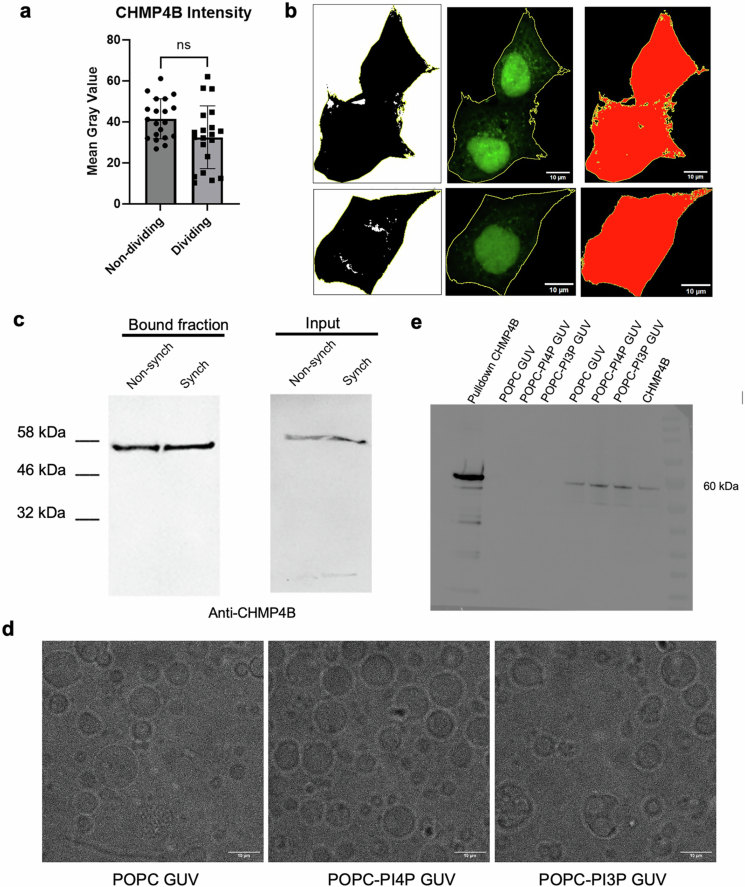
Evaluation of CHMP4B and PIP interactions. **a**) Quantification of GFP-CHMP4B fluorescence intensity in dividing versus non-dividing cells, measured using ImageJ software. Data are presented as mean ± SEM (or specify as appropriate). N = 3. Statistical significance was assessed using a two-tailed Mann-Whitney U test. P = 0.0559 Source numerical data are available in source data. **b)** Representative images of GFP-CHMP4B intensity analysis workflow, quantitated in **a)**. HeLa cells stably expressing GFP-CHMP4B were stained with anti-tubulin. Left panels show binary mask images of tubulin signal, middle panels representative images of GFP-CHMP4B channel showing binary mask generated from tubulin signal. Right panels show images of masked GFP-CHMP4B signal threshold and selection. White arrows show segmented intercellular bridge and midbody. Top row shows a representative cell in cytokinesis and bottom row a non dividing cell, scale bar=10μm N = 3. **c)** Western blot showing input and pulldown of GFP-CHMP4B in synchronized and non-synchronized samples. N = 2 experiments. Unprocessed blots are available in source data. **(d)** GUV images acquired with a spinning disk confocal system. **(e)** Evaluation of CHMP4B and PIP interaction through liposome precipitation assay containing CHMP4B and PI4P brain extract and 18:0-20:4 PI3P followed by Western blotting. Partly purified GFP-CHMP4B was allowed to bind to liposomes of various conditions 100% POPC (no PIP), POPC/PI4P brain mix (90/10), POPC/18:0-20:4PI3P (90/10). Western blot shows presence of GFP-fused CHMP4B in the pellet fraction indicating interaction of CHMP4B with PIP containing liposomes. N = 2 experiments. Unprocessed blots are available in source data. Source data

The change in the localization of CHMP4B and CHMP2A between interphase and cytokinesis could suggest that the lipid species that these ESCRT-III components bind vary as cells progress through the cell cycle. For this reason, we synchronized cells at cytokinesis (Extended Data Fig. 4), as we did with HeLa cells expressing RACGAP1-GFP, and compared the LTMS data from dividing and non-dividing cells. Interestingly, we not only observed that specific lipids interact with CHMP4B and CHMP2A but also that some of these lipids were enriched in synchronized compared with non-synchronized samples. The overall intensity of GFP-CHMP4B expression did not change in synchronized versus non-synchronized cells, as measured by imaging (Extended Data Fig. 6a,b). In addition, western blots showed that comparable amounts of CHMP4B were isolated in LTMS experiments of synchronized or non-synchronized cells (Extended Data Fig. 6c). Together, these data show that ESCRT-III alters its lipid binding according to the cell cycle. Cell cycle-dependent changes in its localization result in a new lipid environment, suggesting that these lipids may be involved in its regulation and/or function (Figs. 4b,d–f,h and Supplementary Table 1). These data also highlight the sensitivity of LTMS and its utility in probing dynamic processes.

LTMS resulted in a variety of phospholipids, especially from GFP-CHMP4B (Figs. 4b,d and Supplementary Table 1), including some anionic phospholipids such as PSs and phosphatidylinositols. This is consistent with the previous in vitro studies showing that CHMP4B has a higher affinity for negatively charged lipids^48^. Neutral lipid species such as PCs, PEs and sphingomyelins (SMs) were also identified, suggesting that CHMP4B (and/or its protein complex partners) could also directly interact with or be surrounded by these lipid species. Ether PEs and ether PCs were also isolated (Fig. 4d, Supplementary Table 1 and Supplementary Note 1). Ether phospholipids are characterized by an ether, rather than ester, bridge between their alkyl chain and their glycerol backbone and are major components of biological membranes^50^. However, apart from their structural roles and a potential role in membrane signalling and trafficking, it is not well known what functions they play in cells. Our result suggests that these lipid species interact with CHMP4B and potentially with other membrane-associated proteins.

The most striking differences in LTMS of dividing and non-dividing cells were in triacylglycerols (TGs) and PIPs (Figs. 4b,e, Supplementary Table 1 and Supplementary Note 1). Four different TG species were increased in the synchronized samples, suggesting a possible specific interaction of CHMP4B with these lipids during cytokinesis. In addition, after performing the derivatization protocol, we detected PIP 18:0_20:4 and PIP_2_ 18:0_18:1. PIP_2_ 18:0_18:1 was the same species identified from RACGAP1, suggesting that this PIP_2_, the most abundant detected in the total HeLa extract (Supplementary Table 2), might have a role in the localization of membrane-associated cytokinetic proteins at the midbody. While we cannot exclude the possibility that the observed PIP binding of RACGAP and CHMP4B could be partly due to a relative enrichment of these lipid species during cell division, we note that different PIPs are pulled down by RACGAP and CHMP4B. Although the same, abundant PIP_2_ is enriched in both experiments, it is not enriched in synchronized control conditions (Figs. 3b and 4b). To further confirm the interactions we observed, we assessed the binding of CHMP4B to one of the lipids we identified by LMTS in a cell-free environment. A strength of LTMS is that it allows analysis of lipid–protein interactions in their native environment, which is characterized by high lipid and protein complexity and diversity. Therefore, a minimalist cell-free system would only partly capture binding akin to the cellular environment. Nevertheless, a strong interaction might be detectable and would validate our LTMS data. PI3P 18:0_20:4 is the only commercially available specific lipid species that we identified, and a mix of PI4P that includes these acyl chains is also available. We synthesized giant unilamellar vesicles (GUVs) that consisted of only POPC or POPC plus 10% PIP (Extended Data Fig. 6d) and incubated these with GFP-CHMP4B that we partially purified from cells by immunoprecipitation^51^. Pleasingly, we observed increased binding to PIP-containing GUVs (Fig. 4c and Extended Data Fig. 6e), further supporting our LTMS data. This result confirms previous in vitro studies showing that snf7, the yeast orthologue of CHMP4B, binds PIPs^52^ and at the same time highlights the sensitivity and high information content obtained by LTMS.

Another difference between synchronized and non-synchronized CHMP4B samples was Ceramide 42:1. Interestingly, another sphingolipid, SM 42:1, was also increased in synchronized samples (Fig. 4f). SMs are ceramides linked through a phosphodiester bond to a choline head group. SM 42:1 has the same number of carbons and double bonds as Cer 42:1, suggesting it might be a related species, although our mass spectrometer is not capable of proving this conclusively as it cannot determine the location of double bonds. These data may indicate that CHMP4B, when isolated from dividing cells, has a higher affinity for sphingolipids with the specific 42:1 configuration. Along with TGs, PIPs, SM 42:1 and Cer 42:1, PE O-39:2 was also observed to be increased in synchronized samples. The open question now is how this variety of lipids contributes to CHMP4B function during cytokinesis.

LTMS identified fewer lipids from CHMP2A: two PC species, especially PC 18:2_18:2, a cholesterol ester species, CE 22:5, and a glycosylated ceramide, HexCer 40:1, were enriched. This indicates that CHMP2A directly interacts with these lipid species or is localized in a subregion rich in these lipids (Fig. 4g and Supplementary Table 1), suggesting that this construct of CHMP2A might localize in a different membrane environment compared with CHMP4B. Interestingly, unlike CHMP4B, CHMP2A interacted exclusively with neutral lipids and did not interact with PIPs. This supports the idea that CHMP2A does not have an affinity for negatively charged lipids^49^. One caveat is that CHMP2A fused to a flexible linker followed by GFP, CHMP2A-L-GFP, was used for the experiment. The presence of this linker is required to observe the expected localization of CHMP2A at the midbody^53^ but may have altered its molecular interactions. The most interesting result for CHMP2A was in the TGs it associated with. As for CHMP4B, there was a significant difference in abundance between synchronized and non-synchronized samples for a specific TG species, TG 16:0_18:1_22:5. We detected 57 TGs in our control HeLa lipidome (Supplementary Table 2), with TG 16:0_18:1_22:5 being barely detectable in HeLa extract (making up ~0.04% of all TGs). However, more abundant species (which collectively make up ~75% of TGs) were not bound to CHMP4B or CHMP2A, suggesting that these proteins can discern between TG species. The fact that two independent experiments with different complex partners pull down the same lipid suggests that TGs might be involved in the interaction of ESCRT-III with membranes during cytokinesis, although it is unclear where in the cell they would meet, as TGs are usually found in lipid droplets or the ER.

**Fig. 5:**
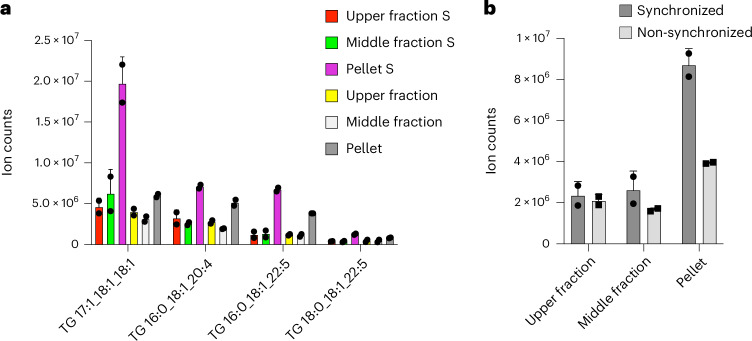
CHMP4B interacts with TGs in membranes. **a**, Bar graph showing the abundance of the TGs species binding CHMP4B after ultracentrifugation in the pellet, middle and upper fractions, in synchronized (S) and non-synchronized samples. Two replicates were run. **b**, Bar graph showing the combined abundance of TGs shown in **a** in the pellet, middle and upper fractions, in synchronized and non-synchronized samples. Two replicates were run. Source data

**Extended Data Fig. 7:**
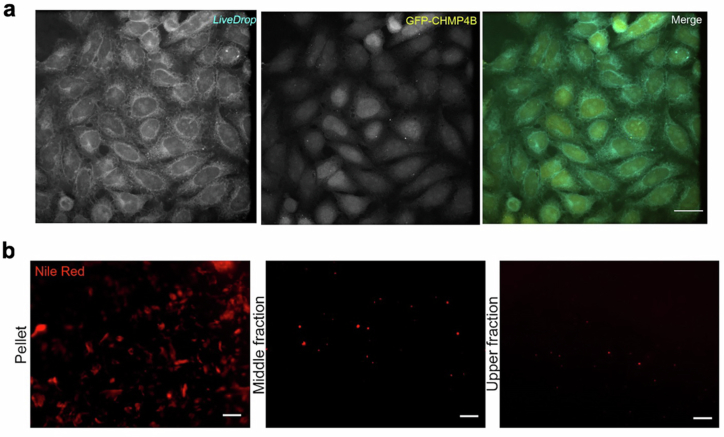
Nascent lipid droplets staining and isolation of CHMP4B-associated membrane fragments with ultracentrifugation. **(a)** Representative confocal images of HeLa cells stably expressing GFP-CHMP4B (yellow) transiently transfected with BFP-LiveDrop (cyan), scale bar=20μm, N *=* 3. **(b)** Microscopy images of membrane fragments stained with Nile Red after ultracentrifugation, scale bar = 5 μm.

Lipid droplets are organelles that store neutral lipids, including TGs. To investigate if CHMP4B interacts with TGs in lipid droplets budding off the ER, we transiently transfected HeLa cells stably expressing GFP-CHMP4B with BFP-LiveDrop^54^ (Extended Data Fig. 7a). LiveDrop is a hydrophobic hairpin sequence derived from GPAT4, which accumulates on lipid droplets as they form in the ER^54^ and is a marker for nascent lipid droplets. We did not observe a strong colocalization between CHMP4B and LiveDrop, suggesting that CHMP4B interacts with TGs in other membrane structures or the cytosol, which could also explain why we only found minor TG species bound to CHMPs. To investigate this, we used ultracentrifugation to separate different cell components and performed LTMS. CHMP4B bound to TGs primarily in the pellet fraction from dividing cells (Fig. 5a,b and Extended Data Fig. 7b). This suggests that CHMP4B interacts with TGs in membrane structures in dividing cells, supporting a role during cell division. These data again highlight the utility and versatility of LTMS, permitting analysis of subcellular fractions from live cells.

## Discussion

Here, we introduce a technique, named LTMS, that allows systematic analysis of lipid–protein interactions from dynamic mammalian cells and subcellular fractions. This versatile technique can be applied to any integral or peripheral membrane protein, including those residing in organelles, as long as it can be tagged with GFP or a similar tag for which affinity reagents are available. Developing a method to investigate lipid–protein interactions is crucial as lipids and membranes are essential in numerous biological processes. Here, we present a model study where we investigate lipid–protein interactions during cell division.

We show that membrane-associated cytokinetic proteins bind specific lipids, contributing to our understanding of why cells regulate their lipidomes during division with high precision^24,55^. Key cytokinesis proteins RACGAP1 and CHMP4B both bound distinct lipids in dividing compared with non-dividing cells, further supporting the observation that membranes are highly dynamic, yet specifically staged, during cell division. Gratifyingly, LTMS identified PIPs as interactors, as would be expected from literature, although with unprecedented chemical precision as the acyl chain configurations of key PIPs were not previously known. Our work also found several unexpected lipid–protein interactions with a range of different lipid species. This includes specific phospholipids and sphingolipids and, surprisingly, a small number of TGs. These TGs are not the most abundant TG species found in HeLa, suggesting that different TGs have distinct binding partners and therefore cellular roles. There is much research to be done to understand these interactions, as the field currently does not have a strong understanding of how cells regulate and mediate the coordinated synthesis, metabolism, transport and interactions of the large number of lipids it produces. LTMS will enable investigations towards this goal.

An advantage of LTMS is that it allows the identification of regions of lipids surrounding a protein of interest. This is important because lipids probably influence protein function by creating a very local membrane nanoenvironment with specific physical and signalling properties, which may be needed for function, including recruitment of proteins and lipids, specific protein conformations or interactions. This local environment can also include other proteins, which could be identified by blotting or proteomics if needed for functional analysis. We expect that LTMS can be expanded to distinguish between tightly bound (classically called non-annular) and associated lipids by adding low concentrations of detergent and/or changing the pH or ionic strength of the lysis and washing buffers, concentrating lipids that bind more tightly to the protein of interest^56^. Varying GFP-protein lysate-to-bead ratios and amounts will also probably result in the detection of lipids with differing binding affinity. In general, one would expect that bead-bound membrane fragments of ~100 nm diameter would contain many more lipid species than we have reported here. However, if these other lipids do not interact with the proteins that are being pulled down (that is, are random bystanders), they would dilute each other, bringing their concentrations below the detection limit of the MS experiment. In addition, abundant non-specific lipids would probably be excluded if they also associated with MyrPalm-GFP in control pulldowns. How many lipids are detected in a particular pulldown therefore gives a sense of the stability and cohesiveness of the membrane region surrounding the protein of interest. For example, the lipid profile of a relatively low abundance protein would probably reflect only highly local interactions, whereas LTMS of a highly abundant protein within a specific environment such as an organelle or vesicle might report on a larger membrane area.

While we present cellular studies in this initial report, we anticipate that LTMS will have applications in biophysical and structural investigations of membrane proteins, with the primary limitation being the somewhat time-intensive nature of lipidomics MS. Currently, proteins in cell-free studies are often embedded in model membranes that include a handful of standard lipid species such as 1,2-dioleoyl-*sn*-glycero-3-phosphocholine (DOPC) or 1-palmitoyl-2-oleoyl-*sn*-glycero-3-phosphocholine (POPC). Applying our lipid isolation protocol would allow reconstitution of a membrane that resembles the protein’s native cellular environment more closely.

This initial report of LTMS identified lipids that surround and interact with GFP-tagged proteins. However, the simple and modular nature of LTMS makes it versatile, and it should be applicable to proteins tagged with other widely used tags, such as FLAG, HA, GST, Halo or SNAP tags, which are frequently used in cell biology^57,58^. It may also be possible to adapt immunoprecipitations if the specific system allows a largely detergent-free workflow. We therefore hope, and expect, that LTMS will become an additional tool in our arsenal of understanding the lipids and proteins of many different membranes. A door has been opened for exploring the role of lipids in cell biology.

## Methods

### Cell culture

HeLa cells, a gift from Prof. Jeremy Carlton, were authenticated by short tandem repeat profiling (Eurofins MWG). HEK293GP and HEK293T cells were from Clontech (Takara Bio). All cell lines were regularly confirmed mycoplasma free by PCR assay (EZ-PCR Mycoplasma Test Kit, Biological Industries) and 4,6-diamidino-2-phenylindole (DAPI) staining. All cell lines were cultured under standard conditions at 37 **°**C and 5% CO_2_ and were maintained in DMEM medium (Gibco, Thermo Fisher Scientific) with addition of 10% fetal bovine serum (Gibco, Thermo Fisher Scientific) and 100 U/100 µg penicillin–streptomycin per litre (Sigma-Aldrich) (complete growth medium). Medium of stable cell lines was supplemented with Puromycin 0.5 μg ml^−1^ or Geneticin (G418) 400 μg ml^−1^ (Gibco, Thermo Fisher Scientific). HeLa cells expressing inducible RACGAP1-GFP or RACGAP1-GFP ∆C1 were further supplemented with Doxycycline 1 μg ml^−1^ (Sigma-Aldrich) for experiments.

### Antibodies and fluorescent dyes

The following primary antibodies were used: mouse anti-α-Tubulin (Thermo Fisher 236 10501, immunofluorescence microscopy 1:1,000), mouse anti-RACGAP1 (Everest Biotech EB05315, western blot 1:1,000), mouse anti-GAPDH (Proteintech10494-1-AP, western blot 1:10,000), mouse anti-GFP (Abcam ab1218, western blot 1:5,000), rabbit anti-GFP (Abcam ab6556, western blot 1:1,000), rabbit anti-CHMP4B (Abcam ab76334, western blot 1:1,000), rabbit and mouse horseradish peroxidase-conjugated secondary antibodies (Stratech-Jackson ImmunoResearch, western blot 1:1,000), Alexa 488 rabbit and mouse (Jackson ImmunoResearch AB_2340846 and Jackson ImmunoResearch AB_2338871, immunofluorescence microscopy 1:500) and Alexa 594 rabbit and mouse (Jackson ImmunoResearchAB_2313584 and Jackson ImmunoResearch AB_2338046, immunofluorescence microscopy 1:500). DNA was stained with 1 mg ml^−1^ DAPI (1:1,000) and neutral lipids with 9-(diethylamino)-5*H*-benzo[a]phenoxazin-5-one (Nile Red, Thermo Fisher N1142, 1:100).

### Cell cycle synchronization

HeLa cells stably expressing the GFP-tagged protein of interest were counted and plated at a density of 4 × 10^6^ cells per T75 flask^59^. Three replicates were used for each experiment. The day after, 100 ng ml^−1^ nocodazole (Sigma-Aldrich) was added for 12 h. Medium was removed, and mitotic cells were collected by mitotic shake-off. Cells were then transferred to Falcon tubes and centrifuged at 200*g*. Pellets were washed with Dulbecco’s PBS to remove the residual nocodazole and resuspended in medium. Cells were then re-plated on 10-cm dishes and released from mitotic arrest for 120 min before the LTMS pulldown. HeLa cells expressing inducible RACGAP1-GFP and RACGAP1-GFP ΔC1 were supplemented with Doxycycline 1 μg ml^−1^ (Sigma-Aldrich) during the release^59^.

### Immunofluorescence

Cells were plated on 11-mm glass coverslips and fixed using 4% paraformaldehyde (Alfa Aesar) in PBS. Cells were fixed for 20 min, permeabilized using 0.1% Triton X-100 in PBS for 5 min and washed twice with PBS/0.5% BSA/20 mM glycine/0.1% NaN_3_ to neutralize unreacted aldehydes for 15 min. Samples were then stained with primary antibodies overnight at 4 °C and secondary antibodies for 2 h at room temperature in blocking buffer (PBS/1% BSA (Apollo Scientific)/0.1% NaN_3_ (Sigma-Aldrich)). Confocal images were acquired at the Nikon Imaging Centre, King’s College London. Acquisition was performed on an inverted Nikon Eclipse Ti microscope equipped with a Yokogawa CSU-X1 spinning disk unit and a Andor Neo sCMOS camera. The microscope was equipped with a 405-nm laser for DAPI excitation, a 488-nm laser for fluorescein isothiocyanate (FITC) excitation and a 561-nm laser for tetramethylrhodamine isothiocyanate (TRITC) excitation. A 40× air lens was used for acquisition. Membrane fragments after ultracentrifugation were imaged using an inverted Nikon Eclipse microscope using widefield epifluorescence (Ti-E) equipped with a Cool SNAP HQ 2, DS-Fi2 Color CCD camera using a 20× objective. CHMP4B fluorescence intensity was quantified from sum-projected confocal images using manual selection and segmentation workflows in Fiji. Individual cells were delineated using the polygon selection tool, after which images were split into the blue, green and red channels as 8-bit stacks. Cell boundaries were defined by thresholding the tubulin signal in the red channel to generate binary masks, which were extracted using the Wand tool and saved as ROI files. These cell masks were subsequently applied to the CHMP4B (green channel) images to restrict thresholding and measurements to the corresponding cellular area. For each cell, mean grey value and cell area were measured.

### Western blot

Laemmli lysis buffer 1.5× without bromophenol blue or dithiothreitol (DTT; 3% SDS (Sigma-Aldrich), 15% glycerol, 0.094 M Tris–HCl pH 6.8 (Fisher Scientific)) was used to lyse cells. Plates were incubated on a heating block for 10 min at 100 °C. After removing the plates from the heating block, each sample was passed through a 25 G needle (Terumo) 10 times. Then, 3× sample buffer (6% SDS, 30% glycerol, 0.003% bromophenol blue, 0.2 M Tris/pH 6.8) and 0.3 M DTT were added to cells to give a final 1× concentration and then lysates were transferred to microcentrifuge tubes and heated for 5 min at 100 °C. Samples were stored at −20 °C until further analysis. Proteins were resolved using SDS–polyacrylamide gel electrophoresis and transferred onto a 0.22-μm nitrocellulose membrane. Proteins were then incubated with antibodies of interest in 5% milk overnight at 4 °C. The following day, nitrocellulose membranes were incubated with secondary horseradish peroxidase-conjugated antibodies (Jackson ImmunoResearch) and imaged.

### Plasmid constructs

pCW57-GFP-2A-MCS was a gift from Adam Karpf (Addgene plasmid #71783), Lact-C2-GFP was a gift from Sergio Grinstein (Addgene plasmid #22852) and mCherry-TOMM20-N-10 was a gift from Michael Davidson (Addgene plasmid #55146). RACGAP1 cDNA was a gift from Prof. Francis Barr^60^. pNG72-CHMP2A-LAP-GFP was a gift from Prof. Juan Martin Serrano^53^. pAG138-BFP-Livedrop and pCMS28-GFP-CHMP4B were a gift from Prof. Jeremy Carlton. pcW57 vector was a tetracycline/doxycycline-inducible lentiviral vector, Tet ON. pcW57-RACGAP1-GFP was created by digesting pcW57 with NheI and BamHI and ligating the GFP insert. The pcW57-GFP vector was then further digested with NheI and EcoRI to insert RACGAP1. RACGAP1 cDNA was amplified by PCR using primers 5′ AAAAAAGCTAGCACCATGGATACTATGATGCTGAATGTG 3′ and 5′ AAAAAAGAATTCCTTGAGCATTGGAGAAGC 3′. A Q5 site-directed mutagenesis kit (NEB) was used to delete the RACGAP1 C1 domain. The following primers were used for deletion: 5′ CCTACCCTGATAGGAACAC 3′ and 5′ GCGCATCCCTCCATTACT 3′. Ligation was performed with a T4-DNA-ligase kit (M0202, NEB). The constructs were verified by DNA sequencing.

### Generation of stable cell lines

Lentiviral and retroviral particles were generated using HEK293T or HEK293GP cells, respectively. Lentiviral transfection mixtures were prepared by adding 1,700 ng plasmid of interest, 1,700 ng pVSVG (Addgene, #138479), 1,700 ng psPAX2 (Addgene, #12260) and 4μg ml^−1^ polyethyleneimine (Sigma-Aldrich) in a total volume of 200 μl Dulbecco’s PBS (DPBS). Retroviral transfection mixtures were prepared by adding 1,200 ng ml^−1^ plasmid of interest, 800 ng ml^−1^ pVSVG and 1μg ml^−1^ polyethyleneimine in a total volume of 200 μl DPBS. The plasmids were then transfected into HEK293T or HEK293GP cells. HEK293GP cells constitutively express gag and pol proteins for retrovirus packaging, and psPAX2 was not added to the transfection mixtures. After 4 h, the medium was replaced and cells were left for 48 h to produce the viral particles. Medium was collected and viral particles were then transduced in HeLa cells.

### LTMS pulldowns

Non-synchronized HeLa cells stably expressing the GFP-tagged protein of interest or MyrPalm-GFP were counted and plated at a density of 10^6^ cells per 10-cm dish^61^. A total of three replicates were used for each experiment. The following day, cells were transferred to ice. Synchronized cells were released from mitotic arrest for 120 min before transfer to ice. The same protocol was then applied for both synchronized and non-synchronized cells. All LTMS experiments were run with cells expressing the GFP-tagged protein of interest or MyrPalm-GFP in parallel. Media were removed, and dishes were washed with DPBS. Cells were then collected in PIPES (Sigma) buffer 20 mM pH 6.8 (+1× protease inhibitor; Roche) by scraping and transferred to pre-cooled Eppendorf tubes on ice. Cells were lysed in 1 ml PIPES in 1.5-ml Eppendorf tubes (Safe-lock tubes, Eppendorf, #022363204) through probe sonication (Sonics) by alternating 20 s on-cycle and 30 s off-cycle on ice for a total of 3 min at 30 KHz. Samples were then centrifuged at 3,500*g* for 10 min at 4 °C. An aliquot of each cell lysate was saved as input lane for western blotting. GFP magnetic agarose beads (Chromotek) were washed twice with PIPES buffer before equilibration. Cell lysate was transferred to new Eppendorf tubes with 40 μl GFP magnetic beads and equilibrated for 1 h at 4 °C in tube rotators. After equilibration, Eppendorf tubes were placed in a magnetic rack, supernatants were discarded and beads were washed twice with PIPES buffer. Beads were subjected to lipid extraction for analysis of bound lipids or, alternatively, beads were resuspended in 3× sample buffer with DTT and boiled for 10 min at 95 °C to dissociate proteins for western blot analysis^61^.

### Confocal imaging of CHMP4B fragments on GFP-trap magnetic beads

An LTMS experiment was performed using a HeLa GFP-CHMP4B stable cell line as described above. Samples were collected at each step of the procedure to analyse the size distribution of GFP-CHMP4B fragments during purification. Specifically, lysate samples without beads, bead-bound fractions after lysate incubation and supernatants remaining after bead binding were collected. At each stage, samples were diluted 1:10 to enable optimal visualization of GFP-CHMP4B fragments. Images were acquired using an AXR point-scanning confocal microscope equipped with a super-resolution NSPARC detector (optical resolution 100–120 nm) at the Nikon Imaging Centre at King’s College London. Fragment size quantification was carried out using NIS Elements software. Quantification of fragment lengths was performed in the *xy* plane.

### Ultracentrifugation for subcellular fractionation

To isolate cytoplasm and membrane fragments from GFP-CHMP4B cells, samples were centrifuged in Eppendorf tubes at 720*g* for 5 min. Supernatants were transferred in new Eppendorf tubes on ice and centrifuged at 10,000*g* for 5 min. The new supernatants were transferred in ultracentrifuge Beckman tubes (Gibco, Thermo Fisher Scientific). Samples were then centrifuged at 100,000*g* for 1 h with a Beckman Coulter Optima MAX XP Ultracentrifuge. Pellets were resuspended in PIPES. The middle and upper fractions were processed separately. The different fractions were then transferred to new Eppendorf tubes with 40 μl GFP magnetic beads, and the steps in the section above were followed.

### LTMS lipid extraction from beads

After washing the magnetic beads, samples were transferred to glass tubes^61^. Aqueous phases were removed by using the magnetic rack flipped vertically.

For neutral lipid extraction, lipids were extracted using the method developed by Folch et al.^28^. A total of 250 μl CHCl_3_/MeOH (2:1, v/v) was added to the magnetic beads. Glass tubes were vortexed twice for 60 s, and the CHCl_3_/MeOH mixture was transferred to chloroform-resistant Eppendorf tubes.

For extraction of phosphoinositides, a modified version of the Folch method was used following the protocol by Mucksch et al.^62^. A total of 726 μl CHCl_3_/MeOH/HCl (1 M) (40:80:1, v/v/v) was added to the magnetic beads and the glass tubes were vortexed for 15 min. Then, 720 μl CHCl_3_ was added and the tubes were vortexed for 5 min. Thereafter, 354 μl Cl (1 M) was added and beads were vortexed for 2 min. The tubes were centrifuged at 1,000*g* for 5 min and the lower phase, containing the phosphoinositides, was transferred to fresh Eppendorf tubes. A total of 726 μl CHCl_3_/MeOH/ HCl (1 M) (40:80:1, v/v/v) was added, and samples were vortexed for 10 s. Samples were centrifuged at 1000*g* for 5 min and the lower phase was transferred to the Eppendorf tubes from the first extraction.

Organic phases were evaporated on a heat block at 37 °C under a constant N_2_ flow. After resuspension in the loading buffer, samples were analysed by liquid chromatography MS (LC–MS).

To exclude any unspecific binding to the beads or unspecific lipid association, we used HeLa cells stably expressing MyrPalm-GFP as a control. Samples were normalized by the number of cells plated.

### Phosphoinositide derivatization

For phosphoinositide derivatization, synchronized and non-synchronized cells were counted and plated at the same cell density as used for the Folch extraction^61^. A reaction was performed following the protocol by Hille et al.^63^. A total of 90 μl MeOH/CH_2_Cl_2_ (4/5, v/v) was added to resuspend the dried samples. We added 10 μl trimethylsilyl (TMS)-diazomethane in hexane (2 M) (Sigma-Aldrich) to each extract. The reaction was left to proceed for 30 min at room temperature. Then, 20 μl glacial acetic acid was added to quench the excess of TMS-diazomethane. A beaker with glacial acetic acid was placed in the hood to prevent inhalation of possible volatile TMS-diazomethane. This reaction releases N_2_ (gas) and can be vigorous if a high excess of TMS-diazomethane is neutralized. Organic phases were dried and resuspended in loading buffer. Samples were analysed by LC–MS.

A total of 30 pmol PI(4)P and 60 pmol PI(3,4)P_2_ brain lipid analytical internal standards (ISDs) were used in runs to compare exact mass, retention time and diacylglycerol fragments. ISDs were purchased from Avanti Polar Lipids (LIPID MAPS MS Standards; Avanti Polar Lipids). ISDs were dissolved in CHCl_3_/CH_3_OH/H_2_O (20:9:1, v/v/v) and further diluted to give a concentration of 2 ng μl^−1^.

### Whole-cell HeLa lipid extraction, internal standardization and lipid quantification

We seeded 1 × 10^6^ HeLa cells on a 15-cm dish, cultured under standard conditions with DMEM (high glucose, GlutaMAX Supplement, pyruvate (12077549, Fisher Scientific), 1% penicillin–streptomycin (P0781-100ML, Merck), 10% fetal bovine serum (10500064, Thermo Fisher)) and collected at ~80% confluence^64^. The cells were washed twice with ice-cold PBS (12037539, Fisher Scientific) and the lysate collected in 1 ml 155 mM ammonium bicarbonate-containing cOmplete Mini Protease Inhibitor Cocktail (11836153001, Merck). The lysate was sonicated at pulses of 20 s on, 30 s off over 2 min at 30% intensity and then centrifuged for 5 min at 3,500*g* to remove large debris. A total of 50 µl supernatant was stored at −20 °C to determine protein concentration by Pierce BCA Protein assay (23225, Thermo Scientific), as per the manufacturer’s instructions. The remainder was snap-frozen in liquid nitrogen and stored at −80 °C until extraction.

Lipid extraction was performed using a modified methyl *tert*-butyl ether (MTBE; 34875-1L, Merck) protocol. In brief, 60 µg of cell lysate in 200 µl 155 mM ammonium bicarbonate (15645440, Fisher Scientific) per sample was resuspended in 1.5 ml methanol (MeOH, 179957-1L, Merck). We added 10 µl SPLASH Lipidomix Mass Spec Standard (330707, Avanti Polar Lipids) to each sample before the addition of 5 ml MTBE, followed by a 1-h incubation at room temperature with shaking at 200 rpm. Each sample was spiked with known quantities of each isotopically labelled lipid standard (Supplementary Table 3). ISDs were included at constant concentration across all samples, including two negative controls (blanks) where no sample has been added, to normalize extraction efficiency and allow quantitative comparison of lipid abundances. Two blank samples containing no ISD were included to identify the ISD peaks.

Following MTBE incubation, phase separation was induced by addition of 1.25 ml LC–MS-grade H_2_O (10505904, Fisher Scientific) and incubation at room temperature for 10 min. Samples were centrifuged at 1,000*g* for 10 min, and the upper organic phase was collected. A total of 2 ml MTBE:MeOH:H_2_O (10:3:2.5, v/v/v) was added to the lower phase and the samples were again incubated at room temperature for 10 min and centrifuged at 1,000*g* for 10 min. The upper organic phase was collected and added to the first upper phase collection. Extracts were dried under a gentle stream of nitrogen and reconstituted in 110 µl isopropanol (190764-2.5L, Merck):acetonitrile (1000292500, Merck):water (2:1:1, v/v/v) for LC–MS analysis. All samples were randomized before MS acquisition to minimize batch effects^64^.

### Quantification

Absolute concentrations of lipid species were estimated by comparison with the corresponding SPLASH ISDs. Nanomolar concentrations were calculated using the molar concentration of each internal and the ion counts (peak areas) of each analyte and the matched ISD.

$${\left[\mathrm{nM}\right]}_{\left\{\mathrm{lipid}\right\}}=\left(\left(\frac{{I}_{\left\{\mathrm{lipid}\right\}}}{{I}_{\left\{\mathrm{IS}\right\}}}\right){M}_{\left\{\mathrm{IS}\right\}}\right){10}^{6},$$

$\mathrm{where}\,{I}_{\left\{\mathrm{lipid}\right\}}$ is the ion count (peak area) of the lipid species of interest, ${I}_{\left\{\mathrm{IS}\right\}}$ is the ion count of the corresponding isotopically labelled ISD and ${M}_{\left\{\mathrm{IS}\right\}}$ is the known molar concentration of the ISD spiked into the sample.

### Lipidomics analysis by LC–QTOF

Samples were resuspended in 100 μl loading buffer (1:1:2 water:acetonitrile (LC–MS grade):isopropanol (LC–MS grade)) (Honeywell, Fisher Scientific and Merck) and centrifuged at 6,500*g* for 2 min to pellet any particulates to avoid column clogging due to injection of beads or precipitated proteins. A total of 90 μl of each sample was then transferred to autosampler vials (Agilent).

An Agilent 1290 Infinity II ultra-HPLC system coupled to an Agilent 6550 iFunnel quadrupole time-of-flight (QTOF) MS (Agilent Technologies) was used for the reversed-phase ultra-HPLC (UHPLC)–MS analysis. The UHPLC system was equipped with an Acquity UHPLC CSH C18 column (100 × 2.1 mm, 1.7 μm; Waters). The column was maintained at 65 °C and at a flow rate of 0.6 ml min^−1^. The mobile phase was characterized by a mixture of (A) 60:40 (v/v) acetonitrile/H_2_O and (B) 10:90 (v/v) acetonitrile/isopropanol. For analysis in positive mode, the mobile phase was supplemented with 10 mM ammonium formate and 0.1% formic acid, whereas for analysis in negative mode, 10 mM ammonium acetate was added to the mobile phase. Samples were run in a gradient of mobile phase A and B. The UHPLC–MS method was optimized with minor modifications from Cajka and Fiehn^65^. Between injections, the needle was washed with 100% isopropanol.

The UHPLC analytical gradient was set to: B from 15% to 30% (0–2 min); B from 30% to 48% (2–2.5 min); B from 48% to 82% (2.5–11 min); B from 82% to 99% (11–11.5 min) and B kept on 99% for 4 min. In 0.5 min, B returned to its initial conditions and the column was equilibrated for 3 min for the next run.

The QTOF was calibrated in the mass range 50–1,700 *m*/*z*, and spectra were acquired in centroid mode. After MS1 analysis, the samples were aligned and statistically analysed as described in the last paragraphs. An inclusion list with *m/z* values and retention times of the features of interest was then created. The selected features were further investigated by targeted tandem mass spectrometry (MS/MS). The collision energy was set at 25 eV and −20 eV for positive and negative mode, respectively. Agilent tune mix (mass resolving power ~10,000 at full width at half maximum) was used to tune the instrument. The reference solution to correct small mass drifts was composed of *m*/*z* 121.0509, *m*/*z* 922.0007 in positive mode and *m*/*z* 119.0360, *m*/*z* 966.0007 (formate adducts), *m*/*z* 980.0164 (acetate adduct) in negative mode.

### Instrument parameters

The following parameters were used for both positive and negative mode analyses: SheathGasFlow 11, SheathGasTemp 350, Nebulizer (psig) 35, Gas Flow (l/min) 14 and Gas Temp (°C) 200.

### Scan source parameters

The following parameters were used for both positive and negative mode analyses: OctopoleRFPeak 750, Skimmer1 0, Fragmentor 350, Nozzle Voltage (V) 1000 and VCap 3500.

### Lipidomics data analysis

MassHunter Profinder Software (version B.08.00 and B.10.00, Agilent technologies) was used to process and analyse the data. For feature extraction, the ‘Batch Recursive Feature Extraction for Small Molecule’ option was chosen. Between the possible adducts, H^+^, Na^+^ and NH_4_^+^ were selected for positive mode, and H^−^, CH_3_COO^−^ and HCOO^−^ were selected for negative mode. For the correct peak alignments, retention time span was set to 0.500 min and mass tolerance was set to 20 ppm. A retention time window between 0.500 and 12.000 min was analysed. Features originating from the control (beads) and solvents were removed.

Processed data were exported as CEF files from MassHunter Profinder and were imported to Mass Profiler Professional (MPP; Agilent Technologies) for statistical analysis. Replicates for each experimental condition were grouped. Features that were not present in at least 80% of samples were excluded and the remaining features were subjected to statistical analysis by analysis of variance (ANOVA; *P* < 0.05) with a post hoc Tukey honestly significant difference test for each group compared with MyrPalm-GFP (control). According to the MPP analysis, raw data were checked again with MassHunter Profinder. Then, the (*.csv) files containing *m*/*z*, retention time and area of the features were exported from MassHunter Profinder and were further analysed with GraphPad Prism using *t*-tests based on false discovery rate (FDR) with the two-stage linear step-up procedure of Benjamini, Krieger and Yekutieli, with *Q* = 5%.

MassHunter Qualitative Analysis Software (version B.07.00, Agilent Technologies) was used to generate extracted ion chromatograms for lipid species with known *m*/*z* and retention times, such as derivatized phosphoinositides. The areas of the extracted ion chromatograms were then exported and analysed with GraphPad Prism using *t*-tests based on FDR.

Only features showing statistically significant changes between the control (MyrPalm-GFP) and GFP-protein of interest and that passed manual inspection were subsequently selected for MS/MS analysis.

Selected features were analysed by MS/MS, and lipid annotation was performed using LIPID MAPS^29^, MS-DIAL^30^ and MS-FINDER databases, which were used to match the lipid fragments. If the interpretation of the MS/MS spectrum was difficult owing to background noise, total HeLa extracts were used to target the features. In total HeLa extracts, some features are more abundant, which allows reduction of the background noise and assignment of the lipids with more confidence. Otherwise, accurate mass (<5 ppm deviation from expected mass), retention time and the pattern of adduct formation were the parameters considered for the assignment. For the total HeLa lipidome analysis (Supplementary Table 2), lipid assignments were double-checked using retention time dependencies (that is, plotting the number of carbons in acyl chains versus retention time for each group of lipids within a family that had the same number of double bonds)^66^. Lipids that did not align on these plots were removed from the annotation as potential misidentifications. Lipid annotations were performed following the guidelines of the Lipidomics Standard Initiative (https://lipidomics-standards-initiative.org/; Supplementary Note 2).

### Lipidomics statistics

Mass Hunter Profinder Software (version B 10.00) was used for data processing. The ‘Batch Recursive Feature Extraction for Small Molecule’ option was used to select the features of interest. H^+^, Na^+^ and NH_4_^+^ adducts were selected for positive mode and H^−^, CH_3_COO^−^ and HCOO^−^ adducts were selected for negative mode. Retention time span was set to 0.500 min and mass tolerance to 20 ppm.

CEF files were exported from Mass Hunter Profinder Software and imported in Mass Profinder Professional (MPP) for the statistical analysis. Features were statistically analysed using ANOVA (*P* < 0.05) with a post hoc Tukey honestly significant difference test for each group compared with MyrPalm-GFP (control). After deleting the non-significant features in Mass Hunter Profinder Software, the (*.csv) files containing *m*/*z*, retention time and intensity of features were exported. The intensity was analysed with GraphPad Prism using a *t*-test based on FDR.

The features were further analysed by MS/MS. LIPID MAPS, MS DIAL and MS FINDER databases were used for the in silico analysis of the fragments.

### Preparation of GUVs

GUVs were prepared by electroformation using the Nanion Vesicle Prep Pro (Nanion Technologies) as described^67^ previously^68,69^, with minor modifications. In brief, 200 nmol of specific lipids was dried onto the conductive side of the indium tin oxide-coated slide. POPC, 18:0-20:4 PI(3)P and PI(4)P brain mix were purchased from Avanti Polar Lipids. Three lipid compositions were used for GUV preparation, which include POPC, POPC/ PI(3)P (90/10) and POPC/ PI(4)P (90/10). The glass slide was dried in vacuum for ~2.5 h. A medium O-ring was coated with vacuum grease and placed around the dried lipid film. A total of 270 µl of 250 mM sucrose (Fluorochem) was added inside the O-ring, and the second conductive slide was placed on the top, creating a sandwich. Standard protocol for vesicle preparation of 120 min was used with a rise and fall of 3 min. The amplitude was set at 10 Hz, the voltage was 3 V and the temperature was 55 °C. GUVs were stored at 4 °C and visualized by spinning disk confocal microscopy measurements on the following day. For this, 20 µl of the GUV solution was slowly added to a microscope chamber (ibidi) filled with ~400 µl of 250 mM glucose (Sigma-Aldrich) solution for 2 h. This allowed vesicles to settle onto the bottom of the chamber before imaging measurements were carried out.

### Liposome binding assay

GFP-CHMP4B was partly purified from a stably expressing HeLa cell line using GFP magnetic beads^67^. GFP-CHMP4B-HeLa cells were seeded in 15-cm Petri dishes at a density of approximately 20–30 × 10^6^ cells per dish. Cells were detached using KPBS buffer, scraped and lysed by sonication. The lysate was clarified by centrifugation, and the supernatant was incubated with GFP magnetic beads (35 µl) for 1 h at 4 °C with gentle rotation. Following binding, the beads were collected using a magnetic stand and washed with ice-cold KPBS to remove unbound proteins.

For elution of CHMP4B, the beads were first briefly washed with PIPES wash buffer (1–2 min at 4 °C) and then subjected to acidic extraction using 0.1 M glycine–HCl (pH 2.5, 100 µl) with gentle rotation for 1–2 min at 4 °C. The eluted supernatant was immediately neutralized by the addition of 1/10 volume of 1 M Tris–HCl (pH 8.5).

The liposome binding assay was performed as reported^51^. POPC (100%), POPC/PI(4)P (90/10) and POPC/PI(3)P (90/10) GUVs were prepared and hydrated as discussed above. GUVs (65 nmol/50 μg) were allowed to incubate with eluted GFP-CHMP4B protein (20 μg) at room temperature for 30 min and further centrifuged at 100,000*g* for 30 min at 20 °C. The following supernatant and pellet fractions were subjected to western blotting and stained with GFP antibody for CHMP4B detection in supernatant, pellet and total protein fractions. The intensities of individual bands were quantified using ImageJ, and the liposome-bound protein in the pellet fractions was compared with the total protein processed in parallel in the absence of liposomes^67^.

### Visualizing GUVs using spinning disk confocal microscopy

GUVs were imaged using a Yokogawa CSU-X1 spinning disk confocal system mounted on the Eclipse Ti-E inverted microscope with Nikon CFI Plan Apo Lambda 100× oil objective (numerical aperture 1.45), 600 series SS 488 nm and SS 561 nm lasers and an Andor iXon Ultra U3-888-BV monochrome EMCCD camera. Brightfield excitation was used for imaging GUVs. *Z*-stacks were acquired with step size of 0.6 µm for 12 steps. Imaging was performed at 25 °C. Image processing was performed in Fiji^70^.

### Statistics and reproducibility

Statistical analyses were specific to the types of experiments conducted and are described in Methods for the experimental protocols as well as figure legends for specific results (for example, *P* values).

### Reporting summary

Further information on research design is available in the Nature Portfolio Reporting Summary linked to this article.

## Online content

Any methods, additional references, Nature Portfolio reporting summaries, source data, extended data, supplementary information, acknowledgements, peer review information; details of author contributions and competing interests; and statements of data and code availability are available at 10.1038/s41556-026-01928-6.

## Supplementary information

Supplementary Information Supplementary Notes 1 and 2.

Reporting Summary

Supplementary Table 1 Lipids detected by LTMS from membrane-associated proteins in this study. The table displays lipid species detected, including length of the acyl chains, molecular formula, *m*/*z*, error ppm, retention time (Rt) (min), adduct type, annotation software used for identification and mean value of ion counts with s.d. for MyrPalm-GFP (control), Lact-C2-GFP, TOM20-GFP, RACGAP1-GFP, RACGAP1-GFP ∆C1, GFP-CHMP4B or CHMP2A-L-GFP. S represents samples from cells synchronized at cytokinesis.

Supplementary Table 2 Library of lipids detected in untreated wild type HeLa cells and their relative abundance within each lipid family. The table displays the lipid species, mass error, mean value of ion counts with s.d. and relative abundance within each lipid class in negative or positive modes. According to their physicochemical properties, lipids can be ionized in positive mode, negative mode or both. We show all species identified by the primary ionization mode, as well as any species additionally identified by the opposite mode. These separate experiments are combined in this table for better comparison. Colours indicate lipid species pulled down from RACGAP1-GFP, GFP-CHMP4B or CHMP2A-L-GFP (orange) or from Lact-C2 or TOM20-GFP (green). Tabs show plots for each lipid family of retention times versus carbon length for each set of double bounds. Straight lines are a measure of correct assignments.

Supplementary Table 3 Concentrations of SPLASH Lipidomix Mass Spec Standard (Avanti Polar Lipids) added to whole-cell HeLa lipidome samples (presented in Supplementary Table 2).

Supplementary Table 4 Light microscopy reporting table.

## Source data

Source Data Fig. 2 Numerical source data.

Source Data Fig. 3 Numerical source data.

Source Data Fig. 4 Numerical source data.

Source Data Fig. 5 Numerical source data.

Source Data Extended Data Fig. 1 Unprocessed blots.

Source Data Extended Data Fig. 4 Numerical source data.

Source Data Extended Data Fig. 5 Unprocessed blots.

Source Data Extended Data Fig. 6 Numerical source data.

Source Data Extended Data Fig. 6 Unprocessed blots.

## Data Availability

All data supporting this study, including lipidomics data, are available in this Article and its Supplementary Information. This includes MS/MS data used to identify the lipid species reported in the manuscript (Supplementary Note 1). Should any further data be required, they are available from the corresponding author upon reasonable request. Source data are provided with this paper.
