# Supplementary material for: Lipid-trap mass spectrometry identifies lipid–protein interactions in cells
Source: Nat Cell Biol. 2026 Apr 13;28(5):1066–75. doi: 10.1038/s41556-026-01928-6 (PMC13179130; doi:10.1038/s41556-026-01928-6)
Supplement: Supplementary file 1 — Supplementary Notes 1 and 2. [file 41556_2026_1928_MOESM1_ESM.pdf]

nature cell biology

Supplementary information https://doi.org/10.1038/s41556-026-01928-6

Lipid-trap mass spectrometry identifies lipid–protein interactions in cells

In the format provided by the authors and unedited

Supplementary Notes 1

MS2 spectra acquired (black) and *in silico* spectra (red) for relevant lipids shown in figures 2, 3 and 4. The comparison was performed using computer-generated *in silico* tandem mass spectral (MS/MS) database, MS-DIAL, or MS-FINDER. Note that our MS method cannot always distinguish the position of fatty acid attachment on the glycerol backbone nor identify the locations of double bonds, including on ether lipids, where vinyl ethers (or plasmalogens) are a distinct ether lipid species Therefore, indicative structures are shown.

LactC2-GFP (Fig. 2c)

PS 18:1_18:1 as $[M-H]^-$

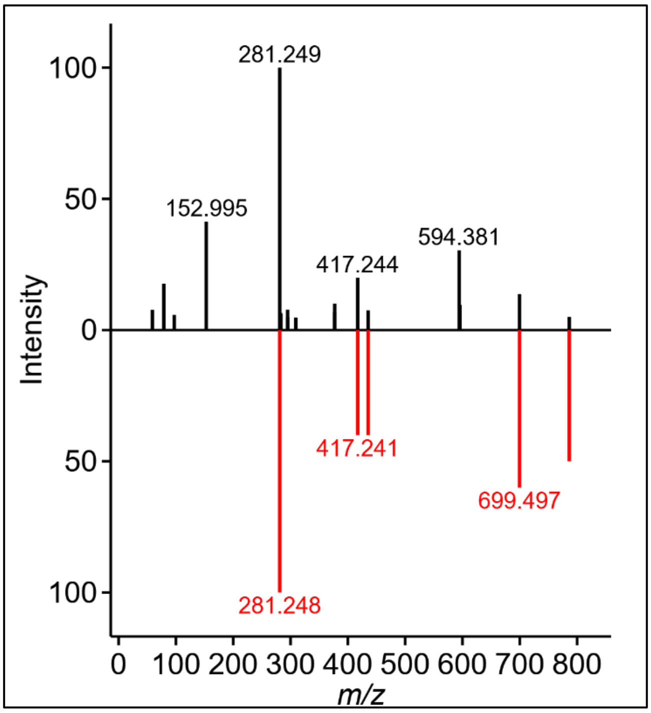

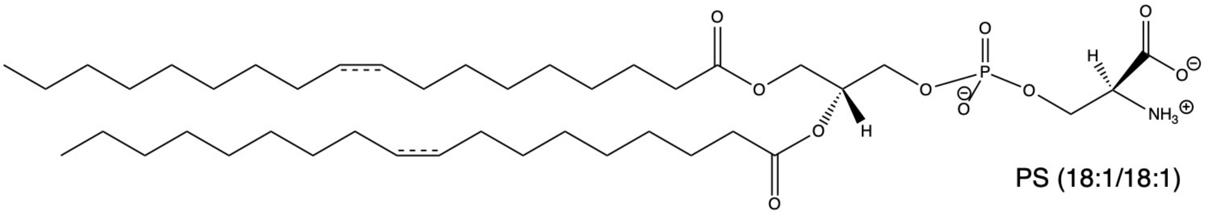

PS (18:1/18:1)

TOM20-GFP (Fig. 2d)

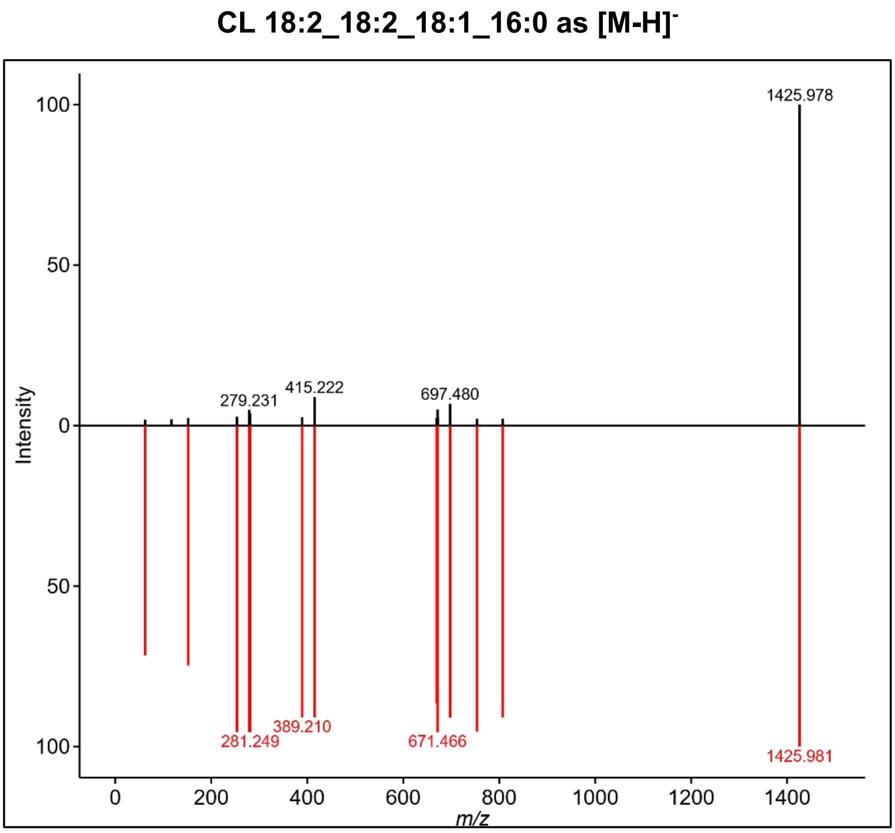

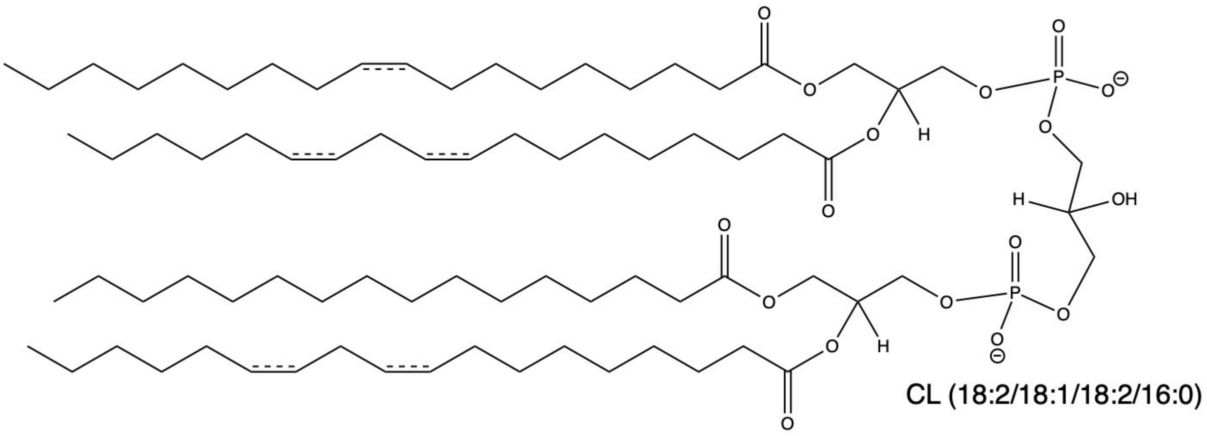

CL 18:1_18:1_18:1_18:2 as $[M-H]^-$

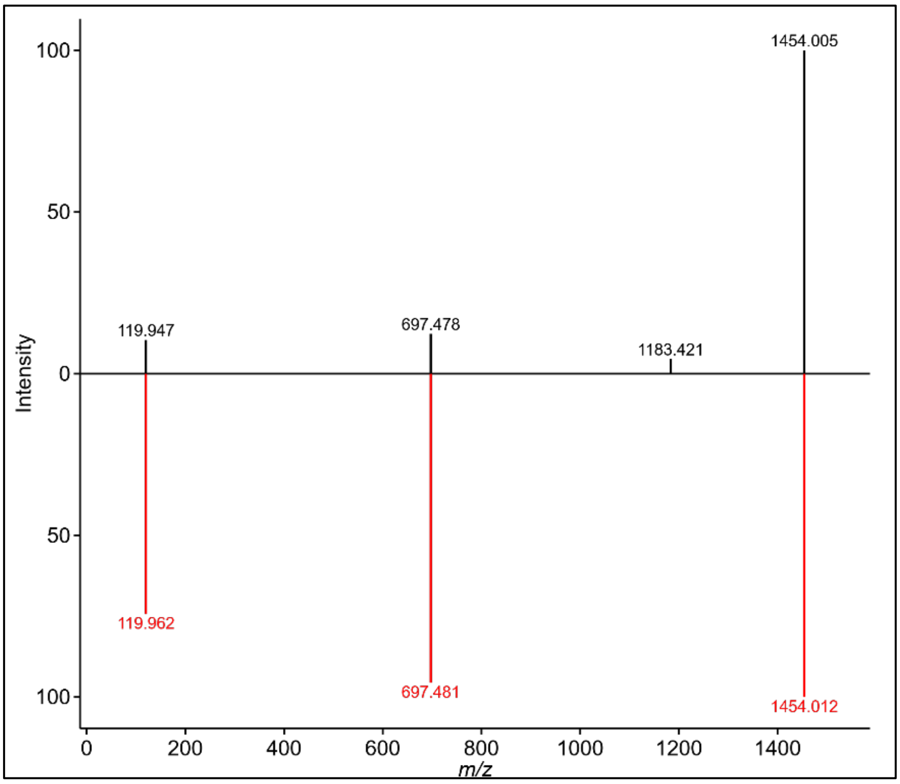

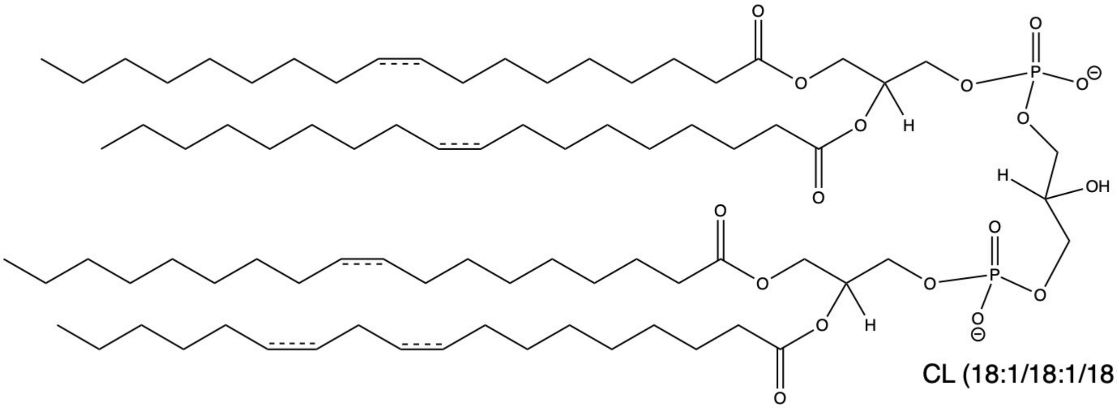

CL (18:1/18:1/18:1/18:2)

PC 18:1_18:2 as [M+OAc]$^-$

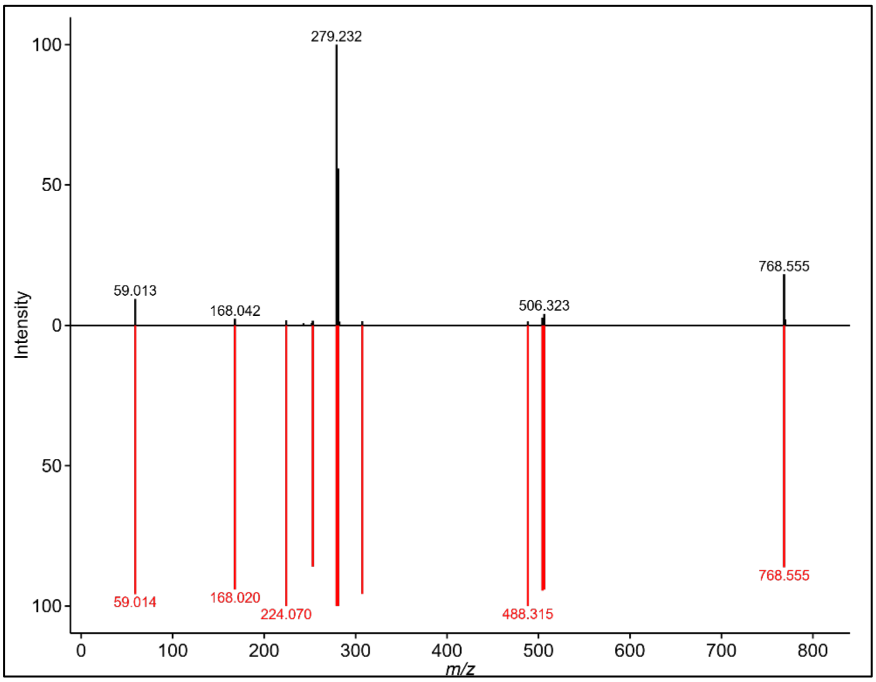

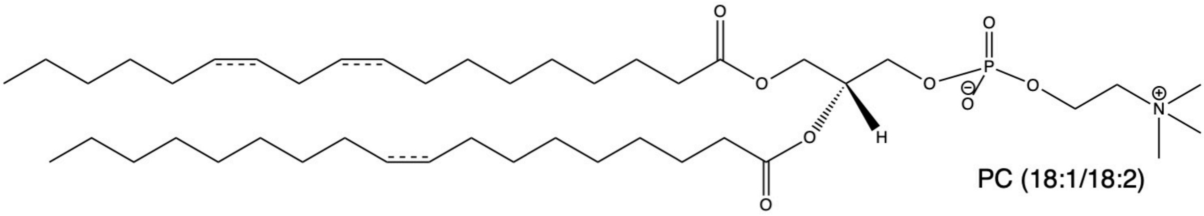

PE 18:0_18:1 as [M-H]⁻

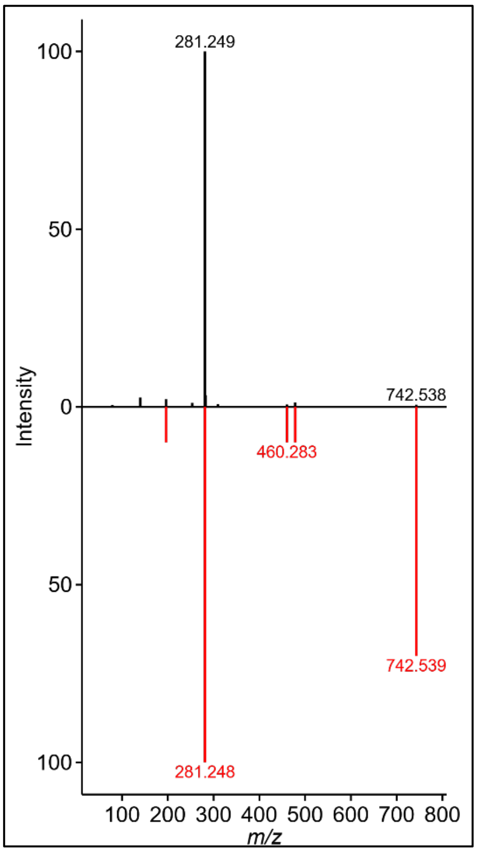

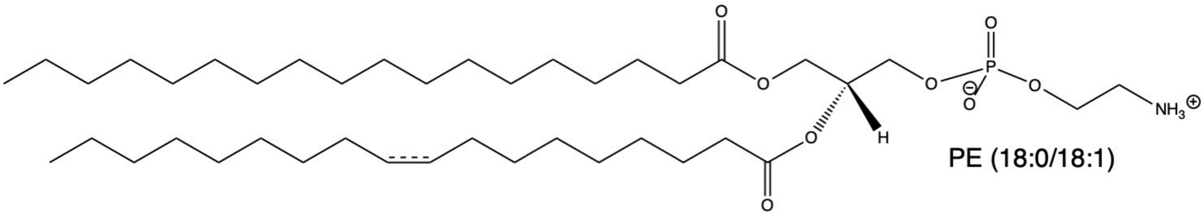

PE (18:0/18:1)

PI 16:0_20:4 as [M-H]$^-$

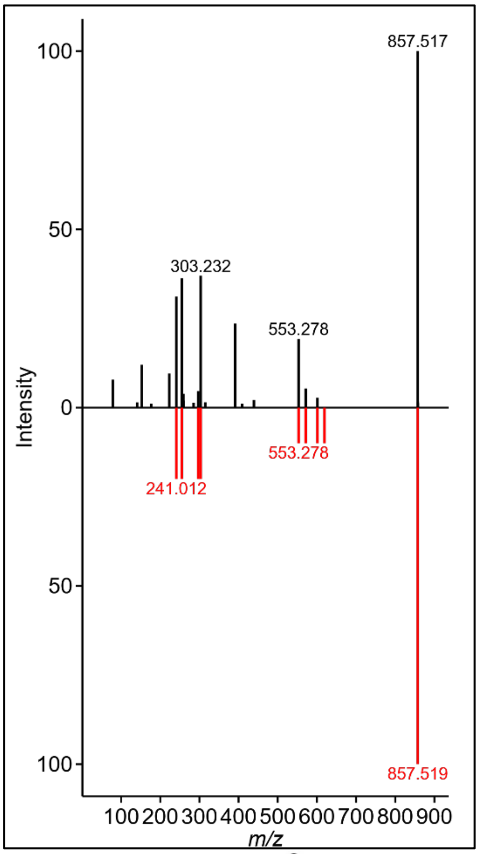

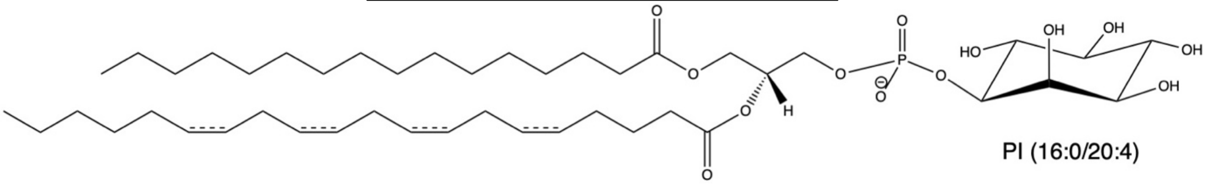

PI (16:0/20:4)

PI 16:1_22:6 as [M-H]$^-$

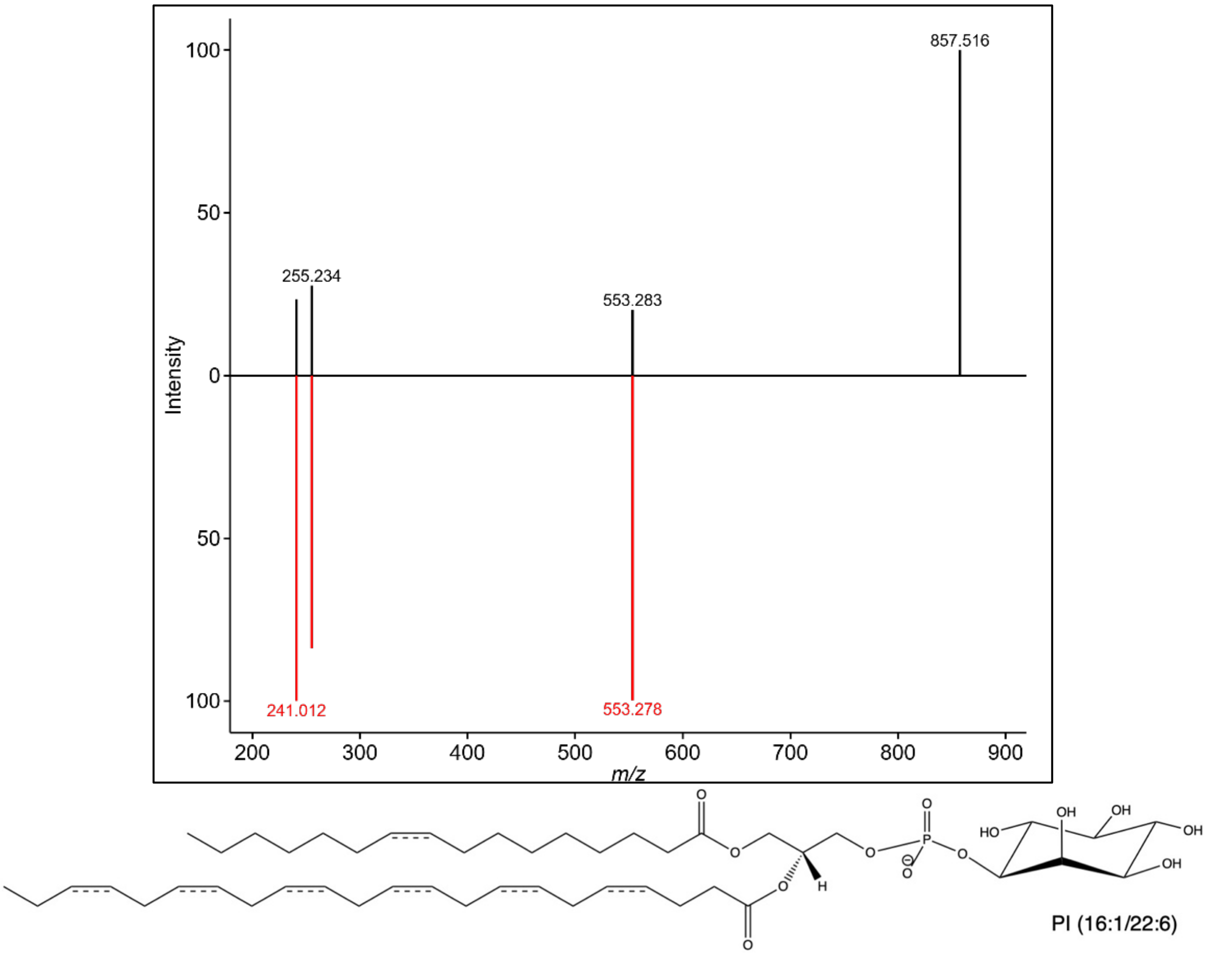

RACGAP1 (Fig. 3b and c)

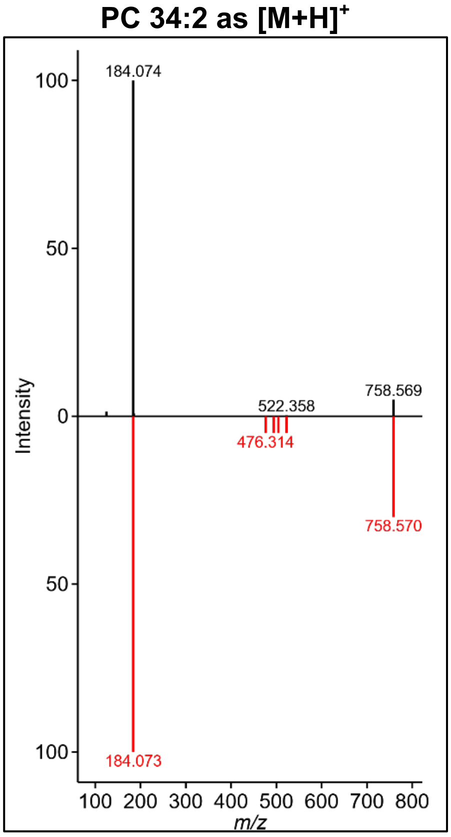

PC O-18:1_16:1 as [M+OAc]⁻

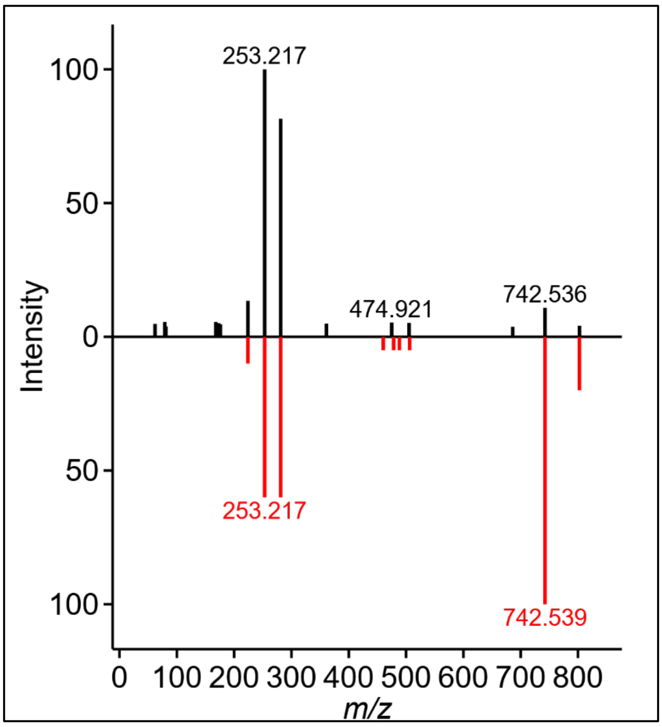

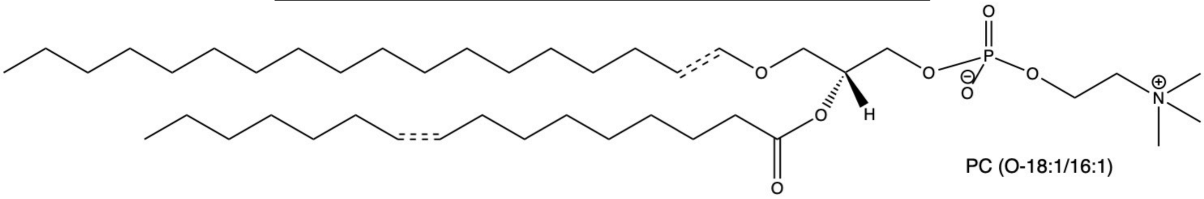

PC (O-18:1/16:1)

PC O-18:0_18:1 as [M+OAc]⁻

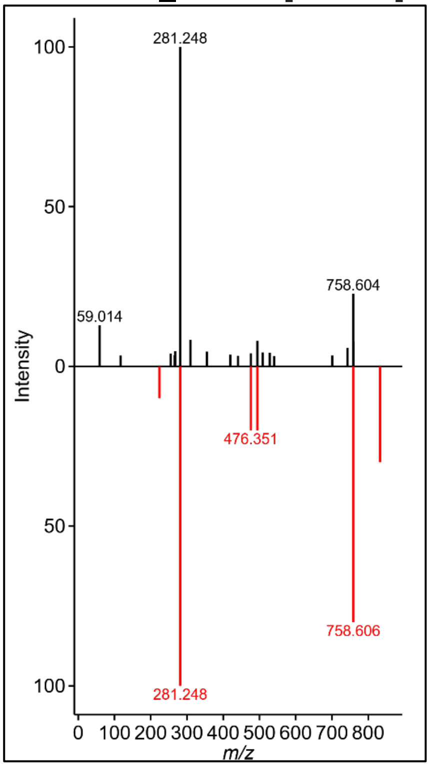

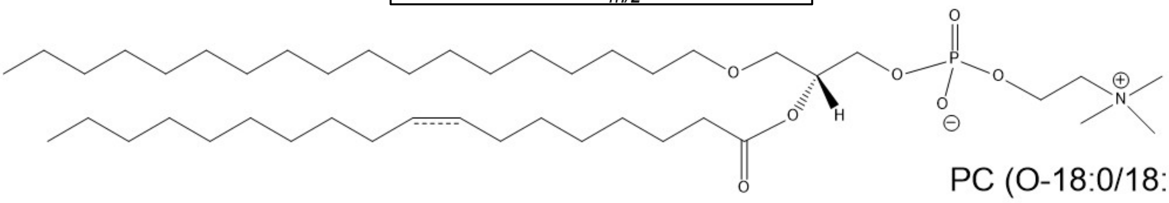

PC (O-18:0/18:1)

PC O-18:1_18:1 as [M+OAc]-

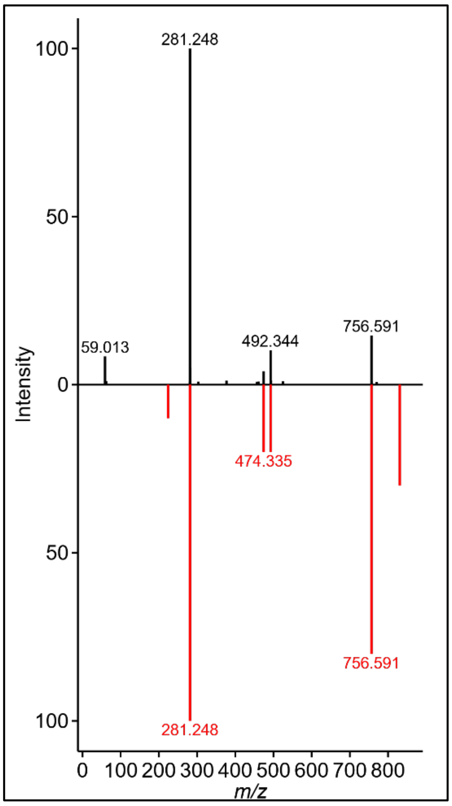

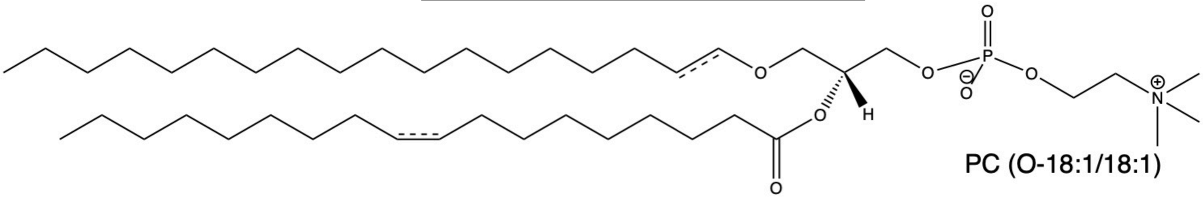

PC (O-18:1/18:1)

PE O-34:3 as $[M+H]^+$

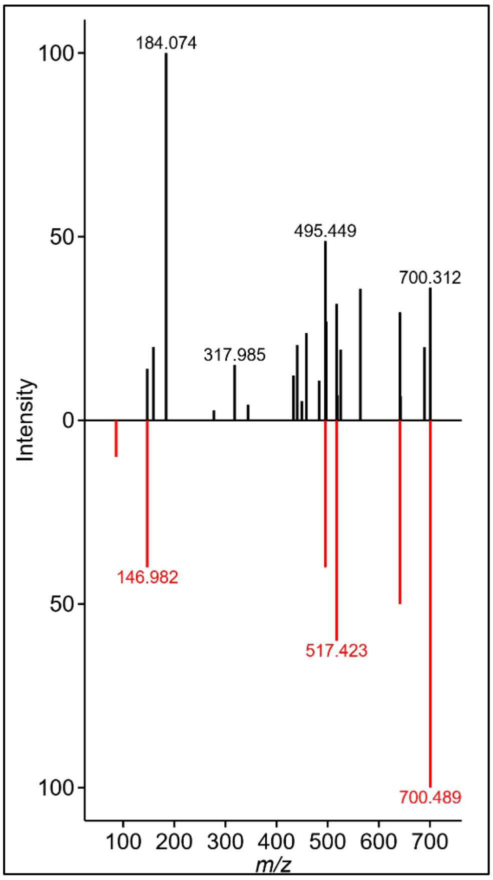

184.074
495.449
700.312
317.985
146.982
517.423
700.489
100
50
0
50
100
Intensity
100 200 300 400 500 600 700
m/z

PE 18:1_19:1 as [M-H]⁻

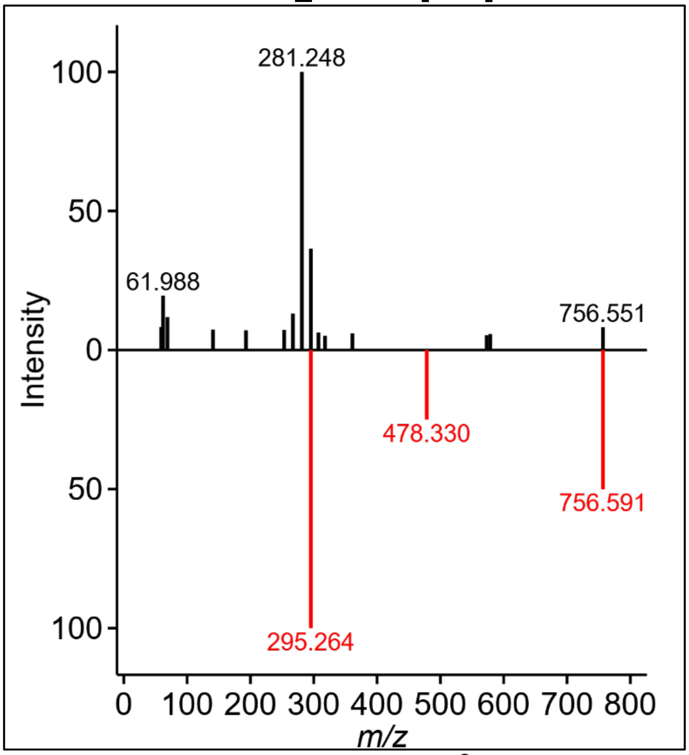

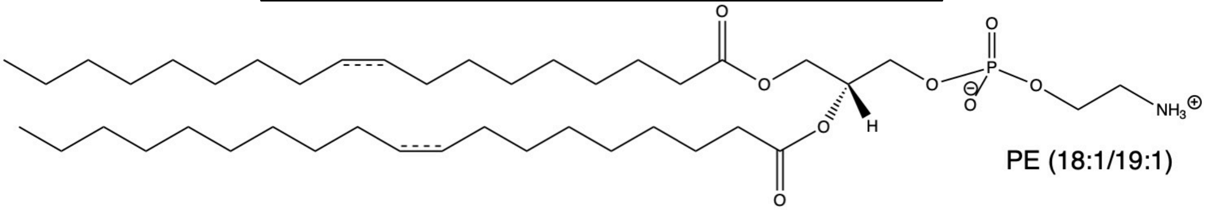

PE (18:1/19:1)

PE 18:1_22:1 as $[M-H]^-$

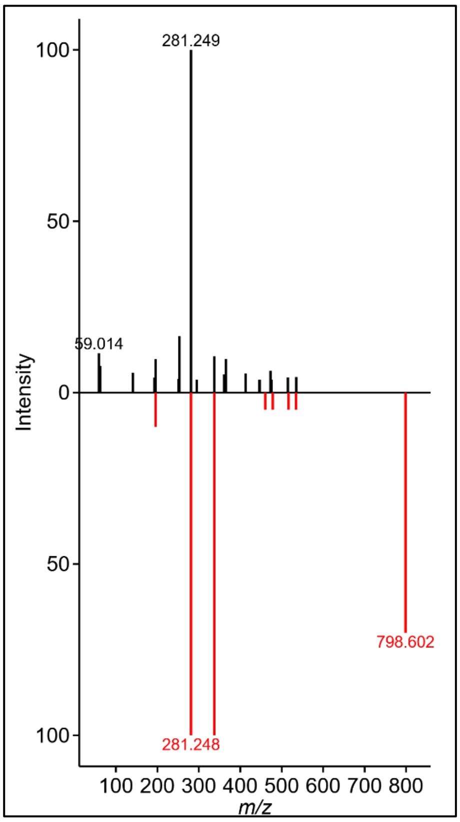

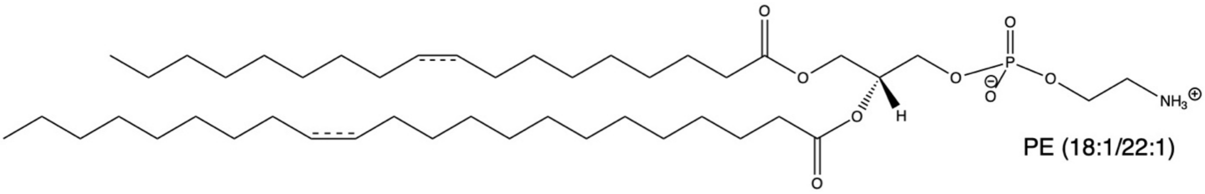

PE (18:1/22:1)

PI 18:0_18:2 as [M-H]-

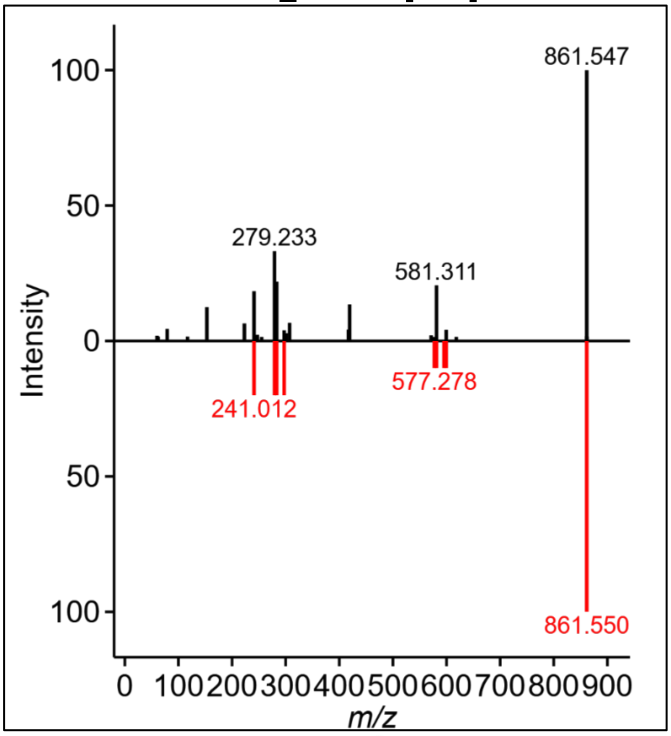

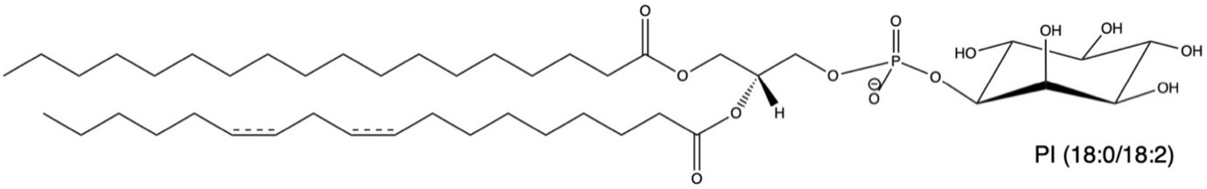

PI 18:0_19:2 as [M-H]⁻

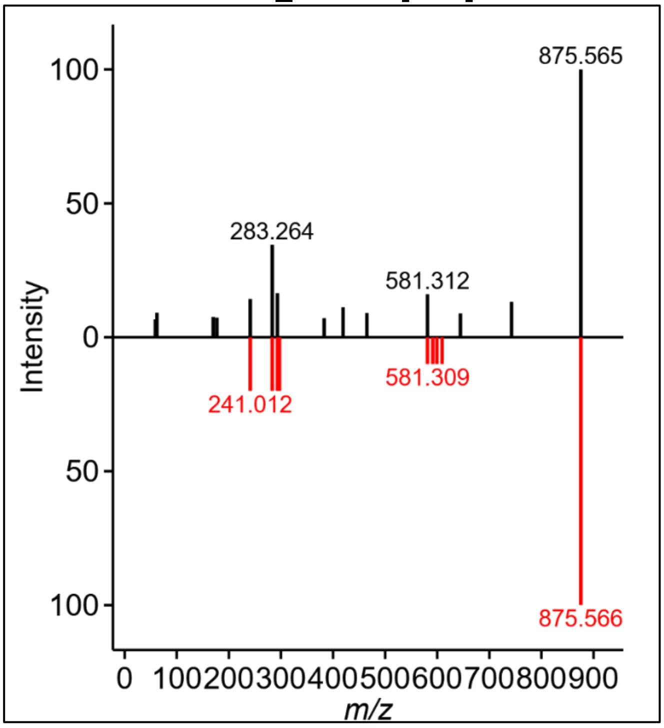

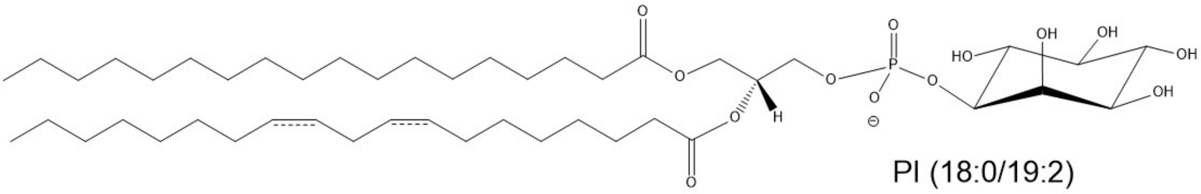

PI (18:0/19:2)

CHMP4B (Fig. 4b, d, e and f)

SM 32:1 as $[M+H]^+$

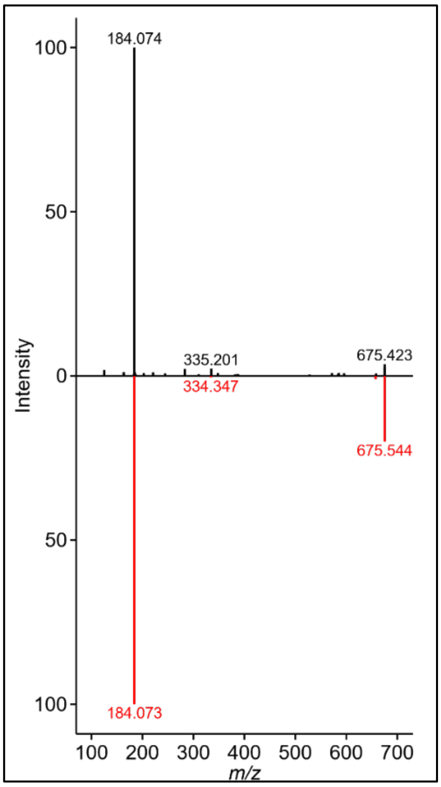

SM 34:0 as [M+OAc]⁻

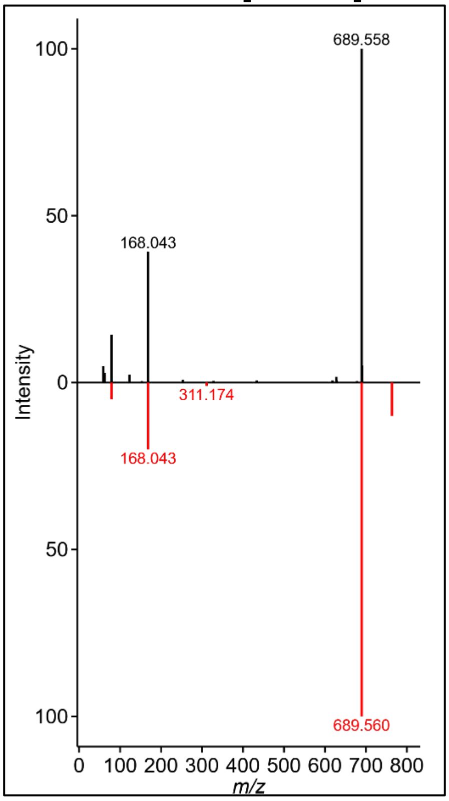

SM 40:0 as $[M+H]^+$

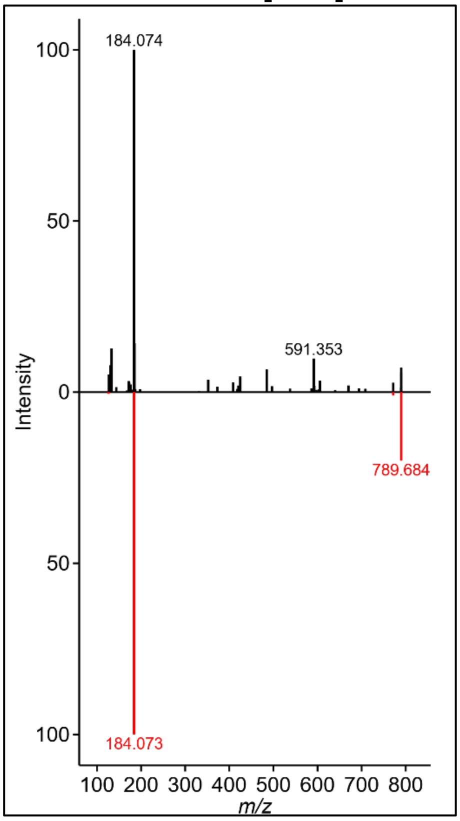

184.074
591.353
789.684
184.073
100
50
0
50
100
Intensity
100 200 300 400 500 600 700 800
m/z

SM 42:1 as [M+OAc]$^{-}$

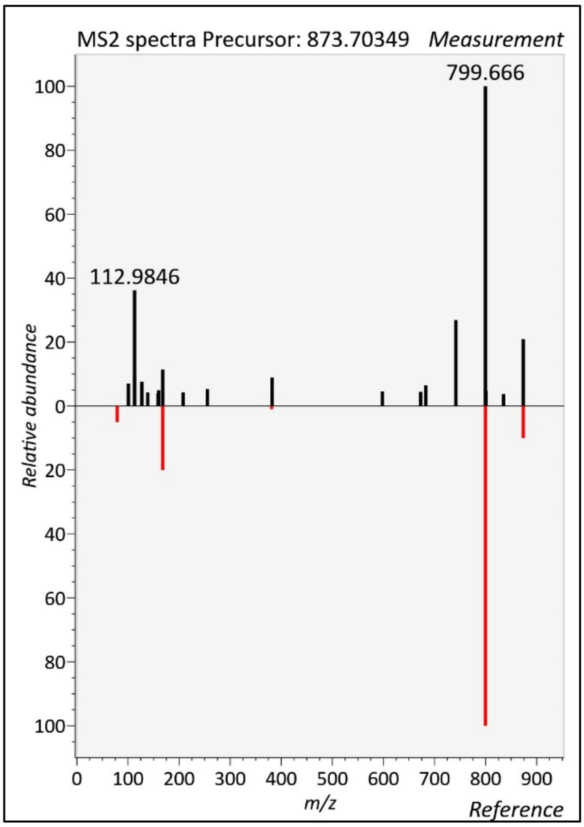

SM 44:2 as [M+OAc]⁻

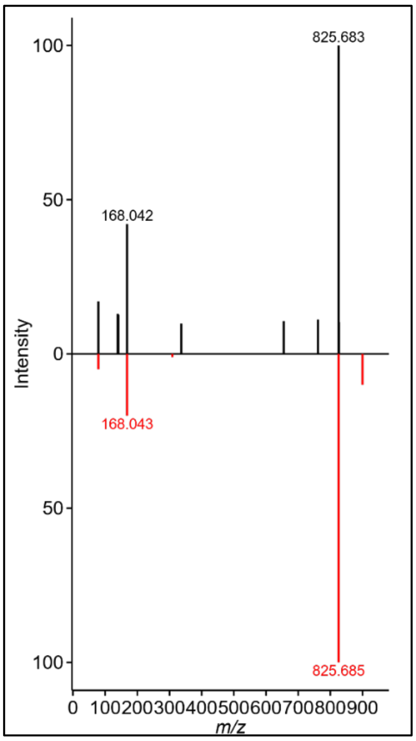

PC 20:4_24:0 as [M+OAc]$^-$

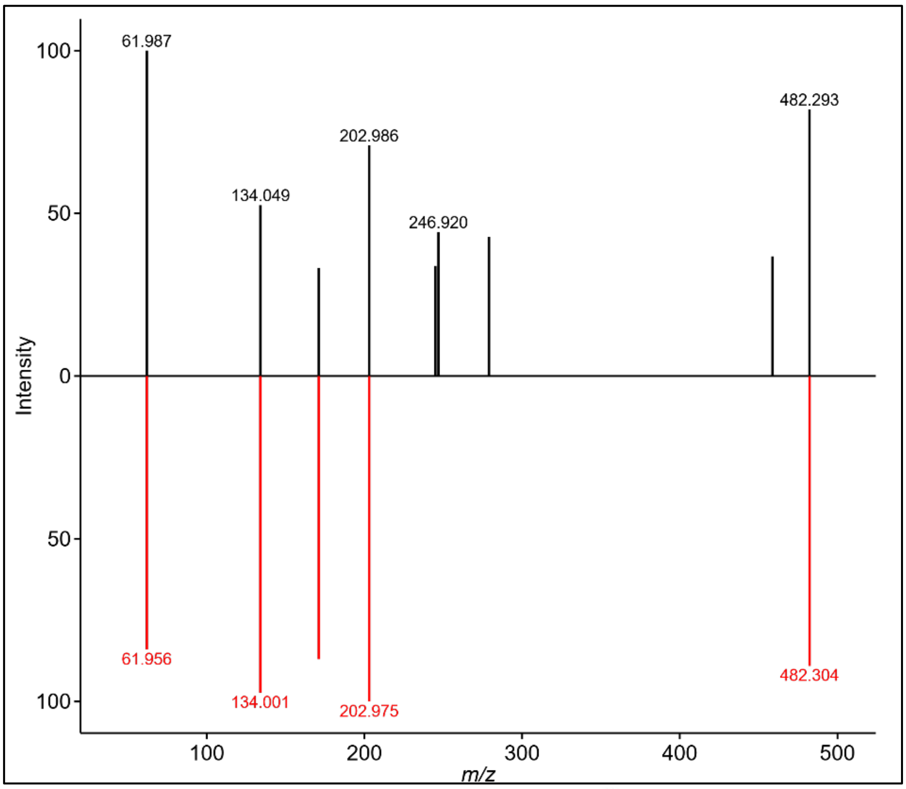

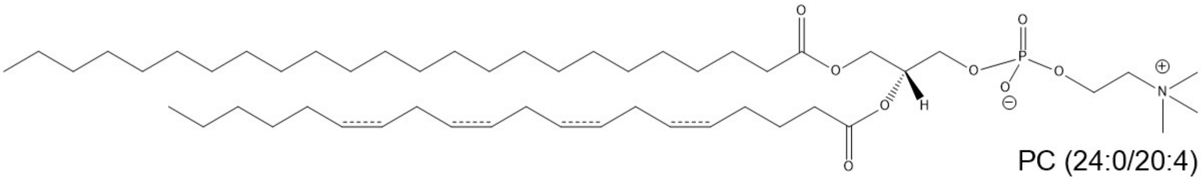

PC (24:0/20:4)

PC O-18:1_18:1 as [M+OAc]⁻

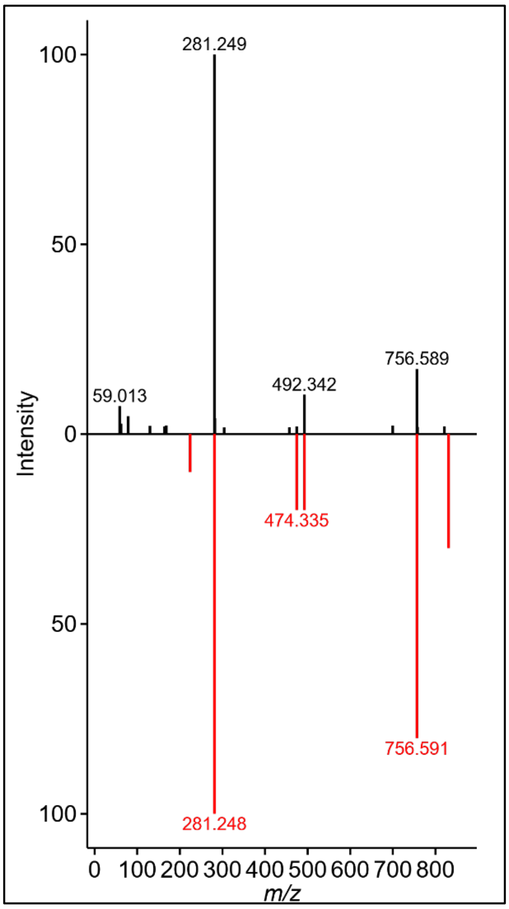

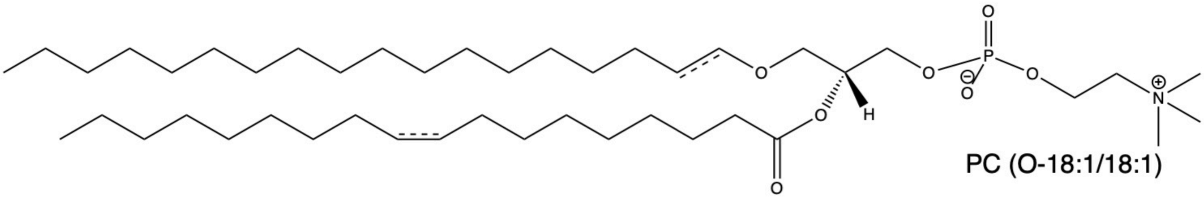

PC (O-18:1/18:1)

PC O-18:1_20:4 as [M+OAc]$^-$

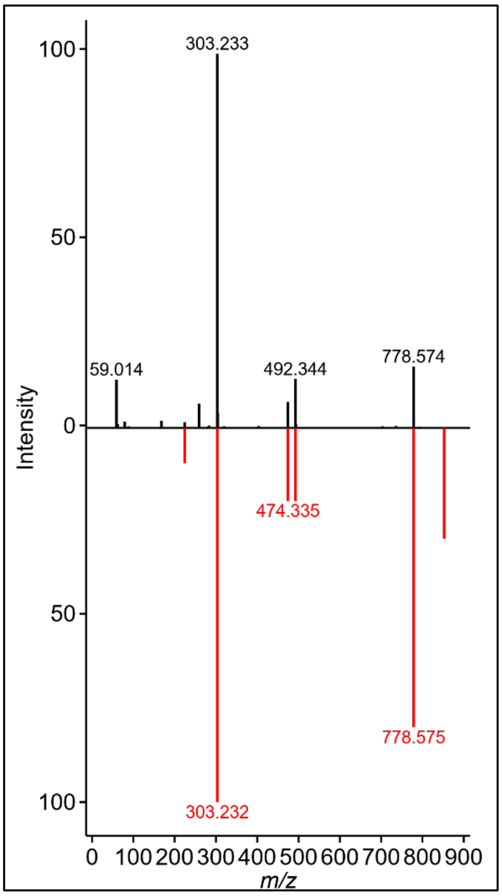

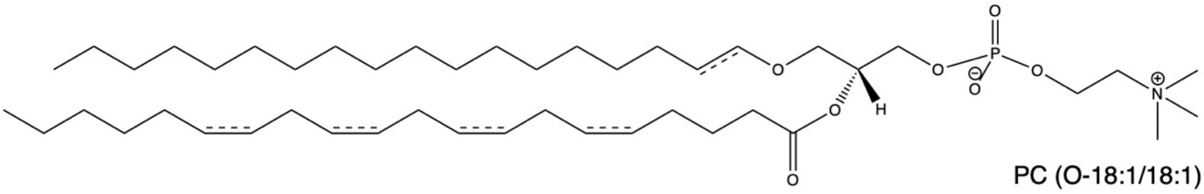

PC (O-18:1/18:1)

PE 16:0_18:0 as [M-H]⁻

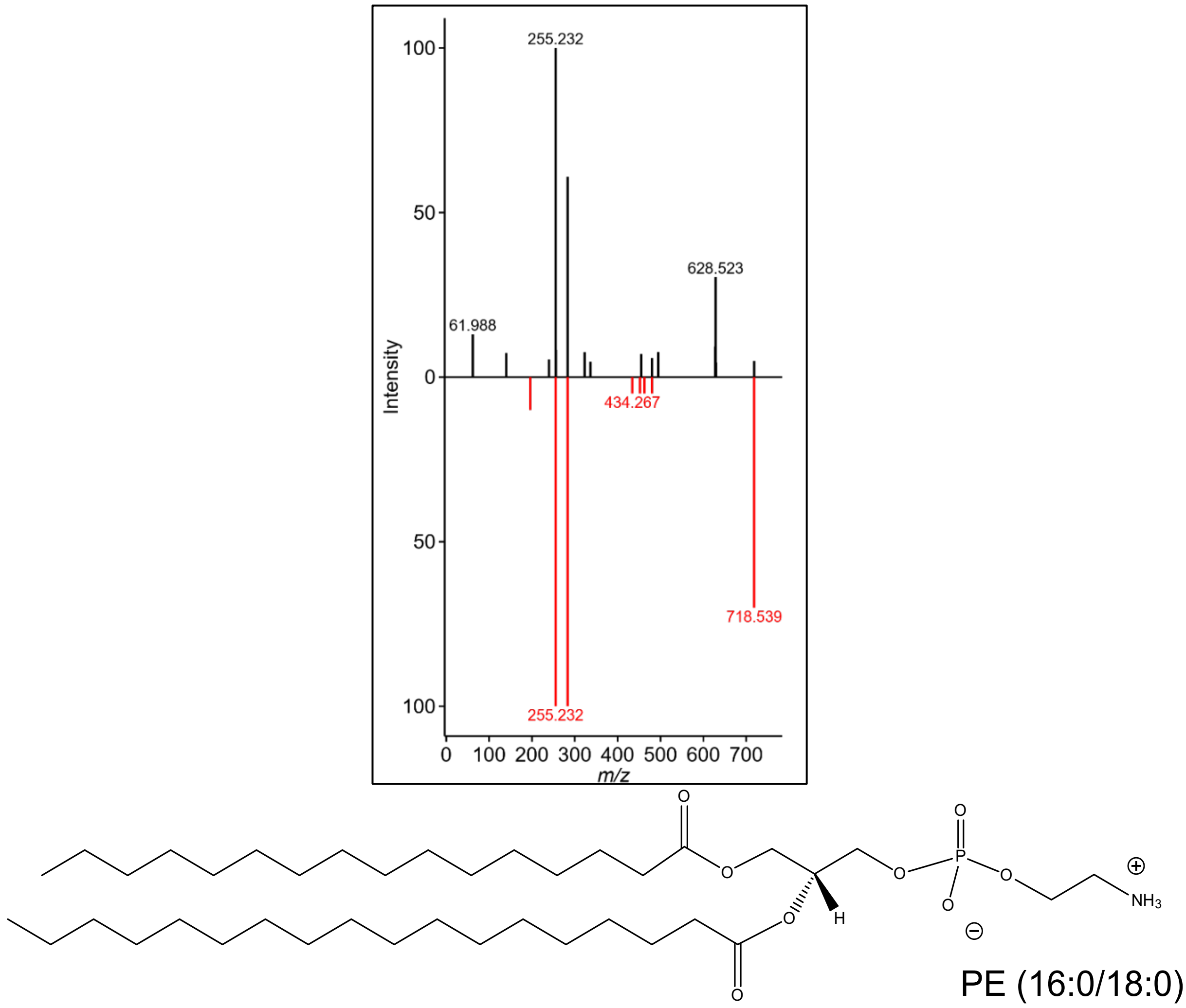

PE (16:0/18:0)

PE 18:0_19:1 as [M-H]⁻

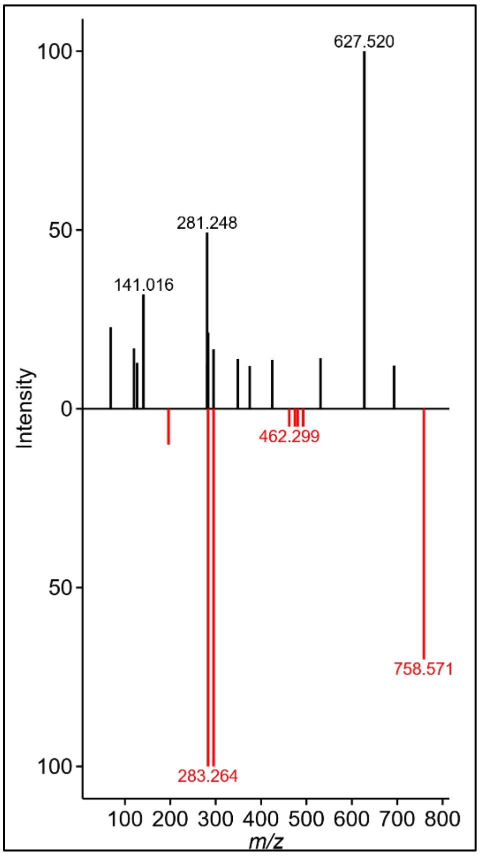

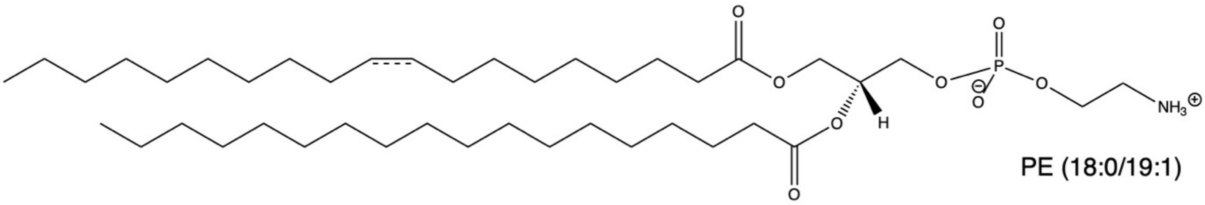

PE (18:0/19:1)

PE 18:0_20:1 as [M-H]⁻

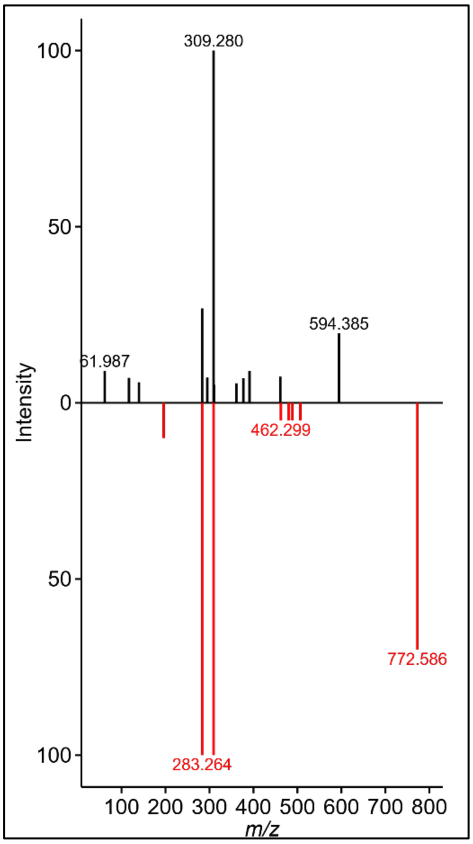

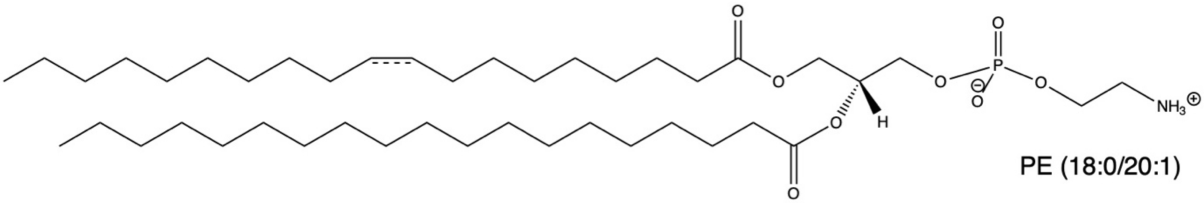

PE 18:0_22:4 as [M-H]$^-$

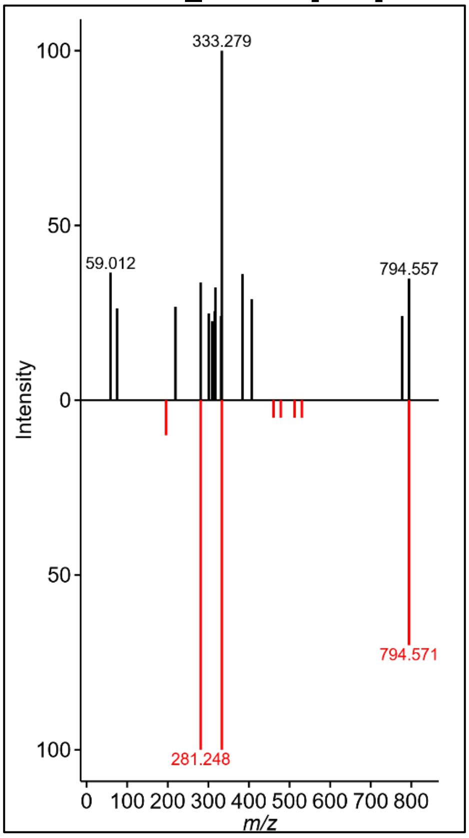

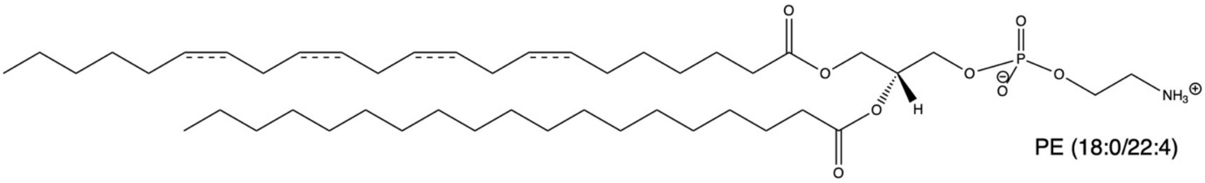

PE (18:0/22:4)

PE 18:0_22:5 as $[M-H]^-$

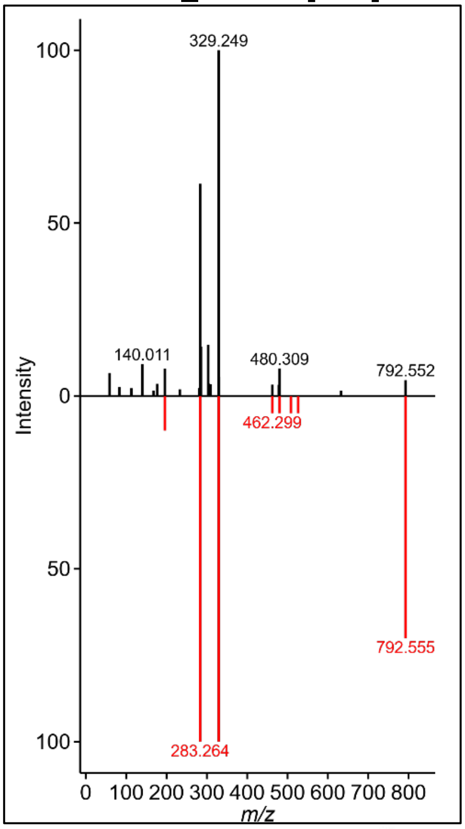

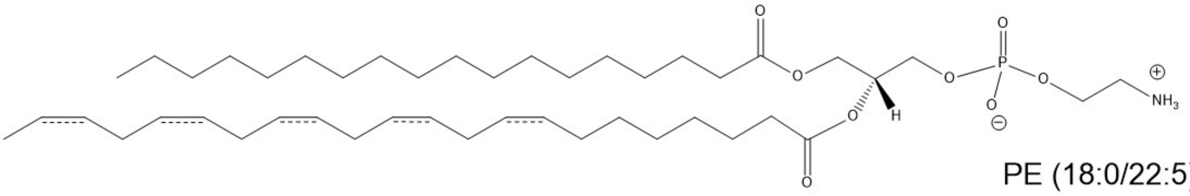

PE (18:0/22:5)

PE O-16:1_16:0 as [M-H]⁻

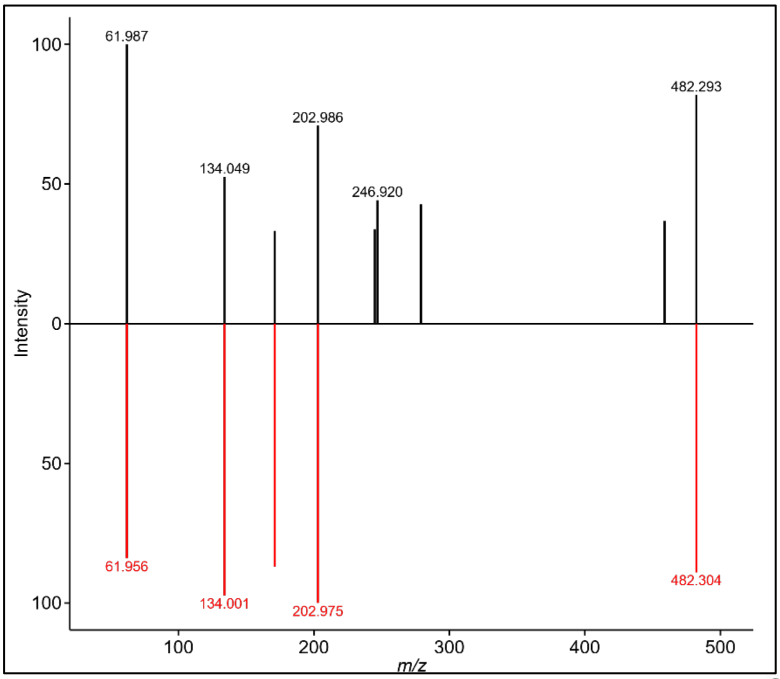

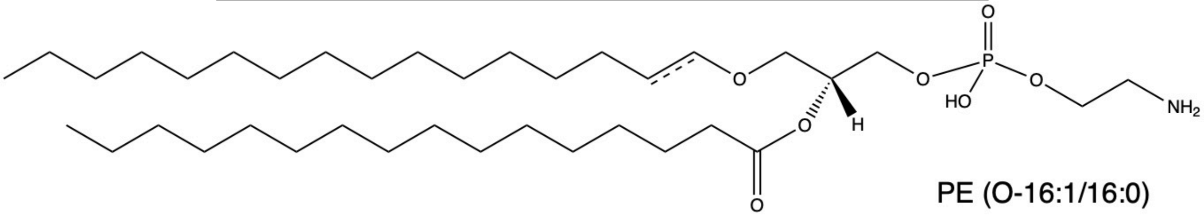

PE (O-16:1/16:0)

PE O-18:1_16:0 as [M-H]$^-$

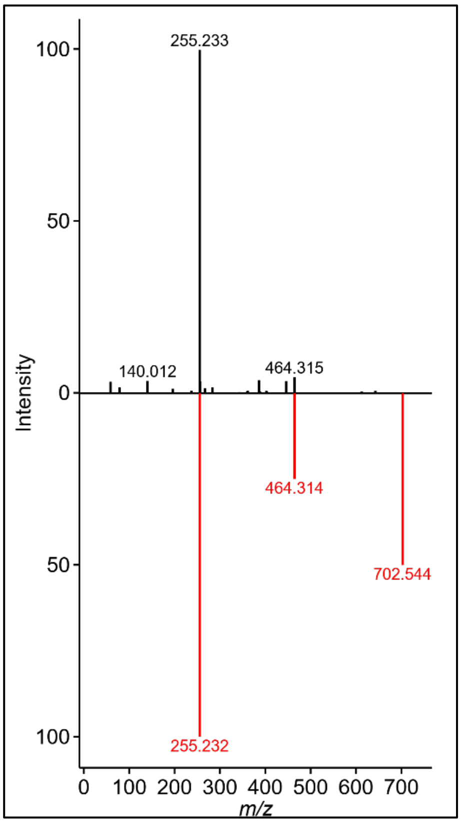

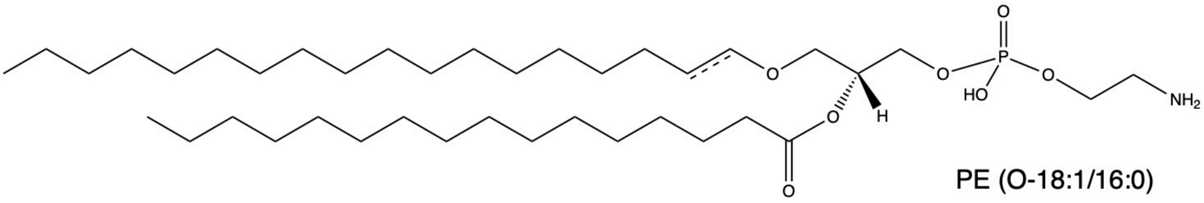

PE (O-18:1/16:0)

PE O-34:3 as $[M+H]^+$

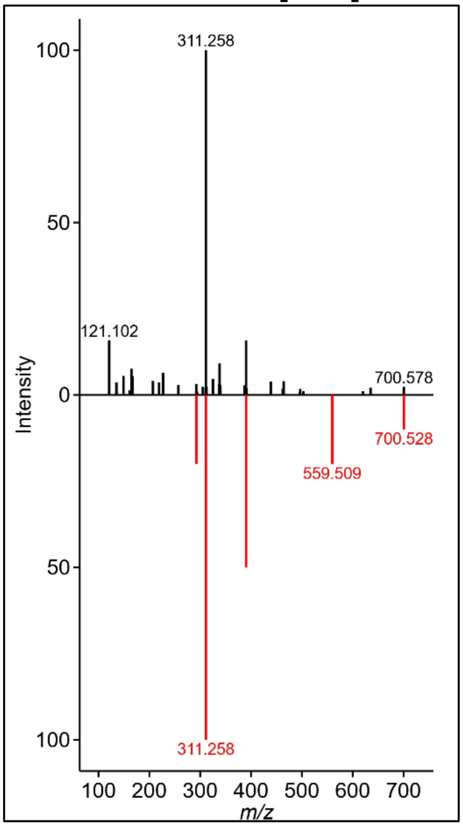

311.258
121.102
700.578
700.528
559.509
311.258
Intensity
100
50
0
50
100
100 200 300 400 500 600 700
m/z

PE O-36:2 [M+H]$^+$

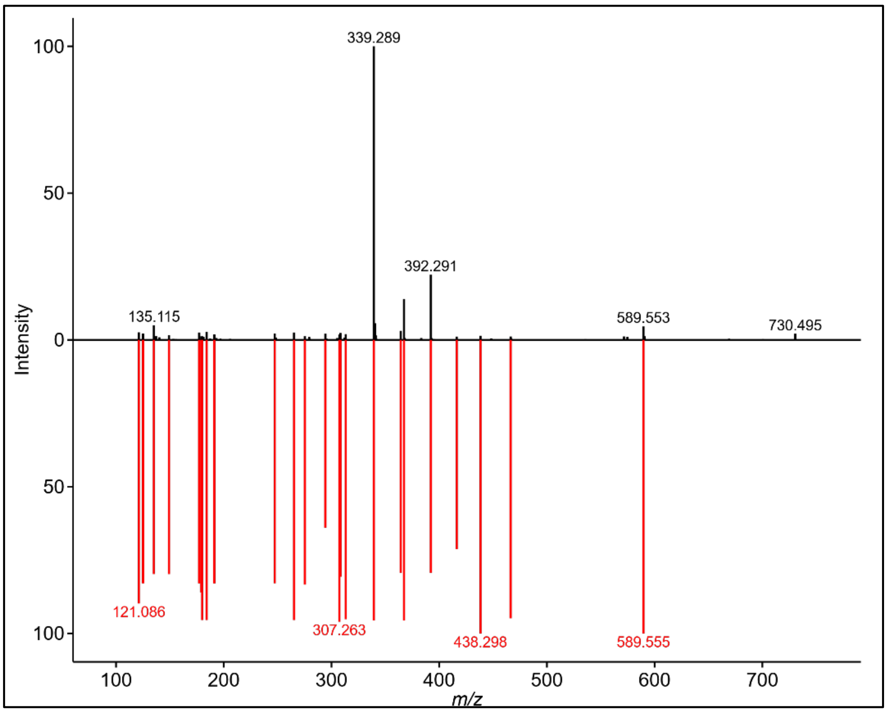

Intensity
100
50
0
50
100
339.289
392.291
135.115
589.553
730.495
121.086
307.263
438.298
589.555
100
200
300
400
500
600
700
m/z

PE O-18:2_20:1 as $[M-H]^-$

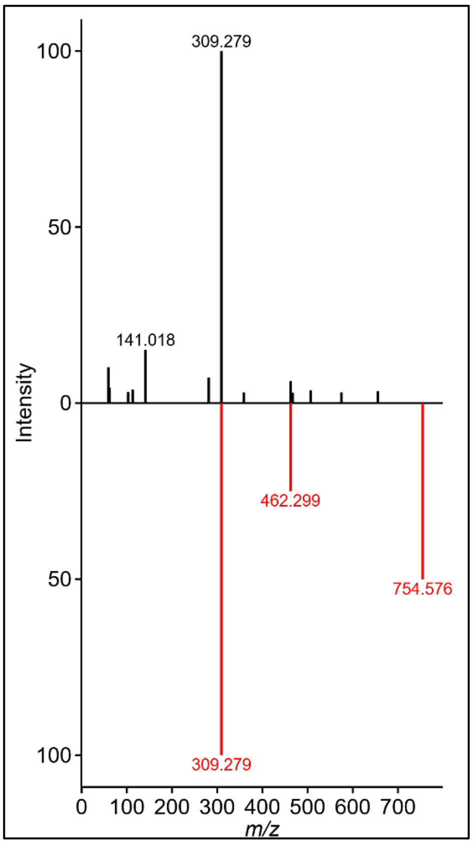

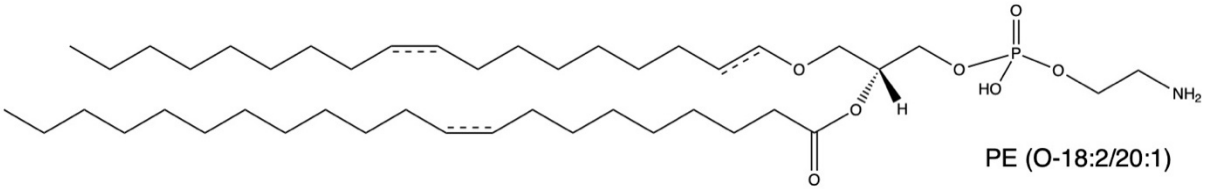

PE (O-18:2/20:1)

PI 34:2 as $[M+H]^+$

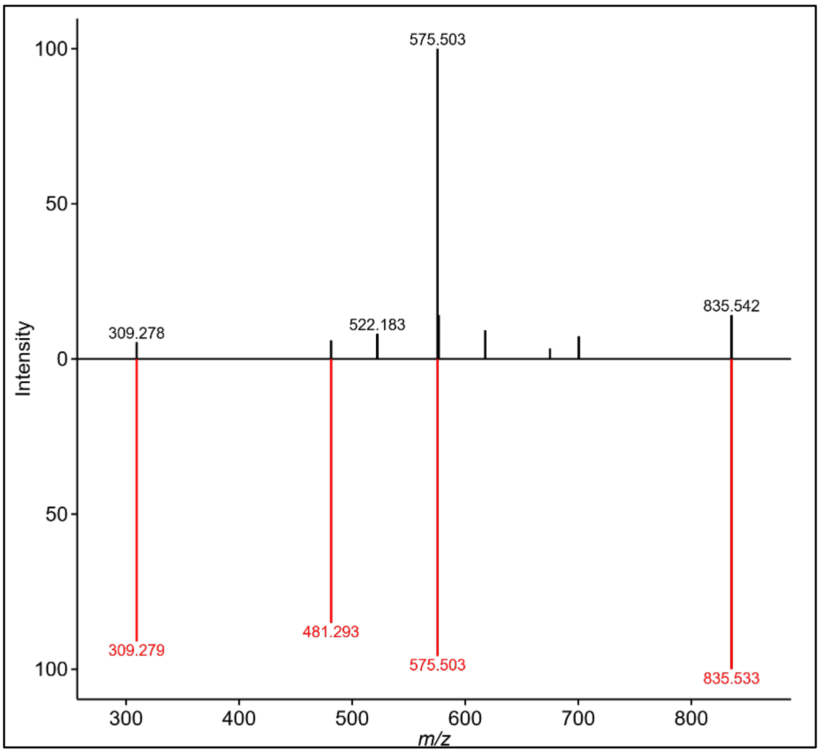

575.503
835.542
309.278
522.183
309.279
481.293
575.503
835.533
Intensity
m/z

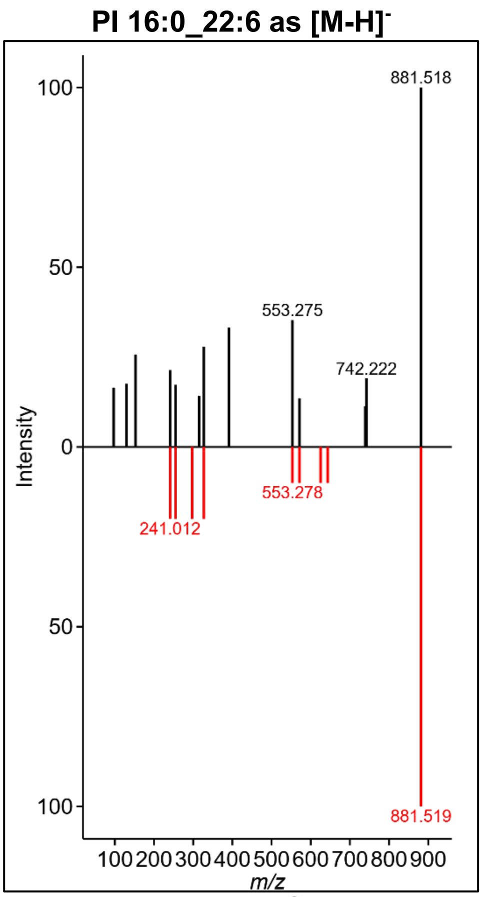
PI 16:0_22:6 as [M-H]-
100
50
0
50
100
Intensity
881.518
553.275
742.222
241.012
553.278
881.519
100 200 300 400 500 600 700 800 900
m/z

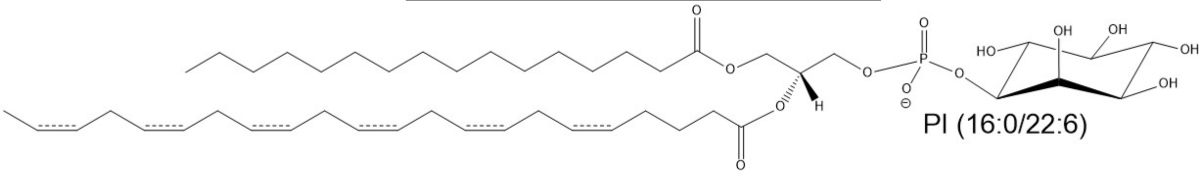
HO
OH
OH
OH
OH
H

PI (16:0/22:6)

TG 17:1_18:1_18:1 as $[M+NH_4]^+$

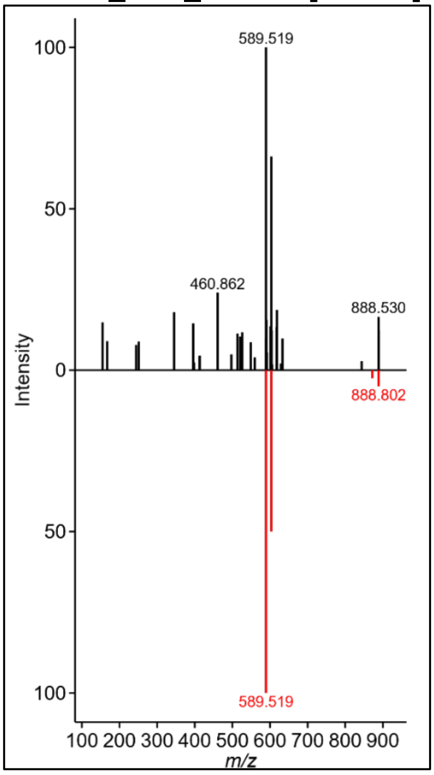

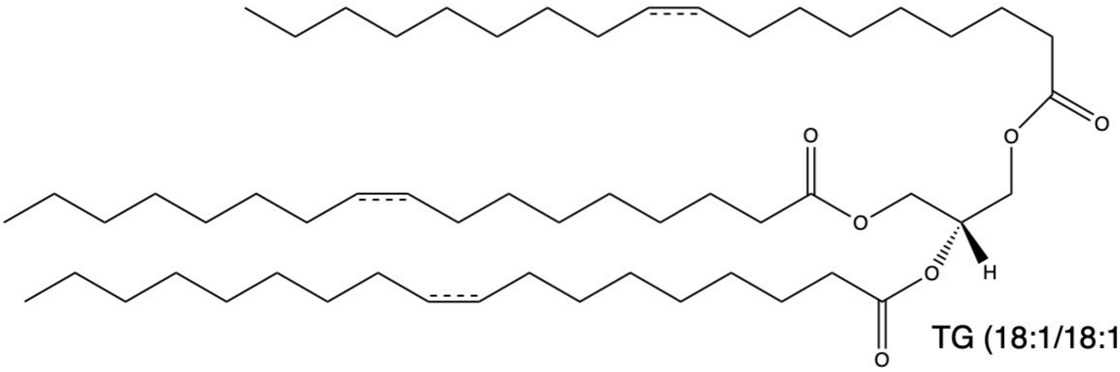

TG (18:1/18:1/17:1)

TG 16:0_18:1_20:4 as $[M+NH_4]^+$

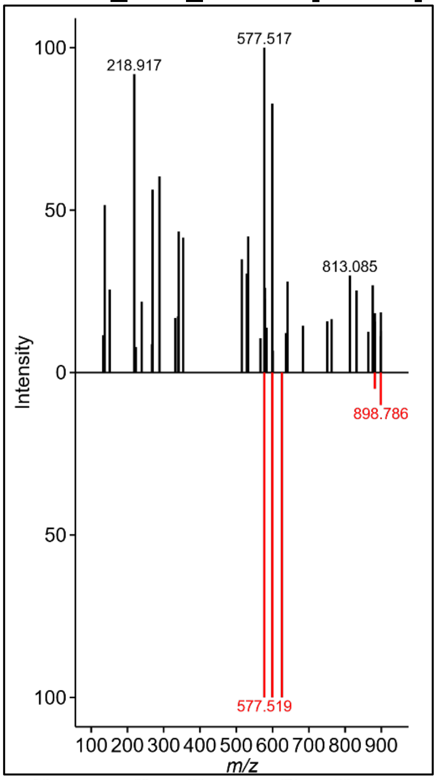

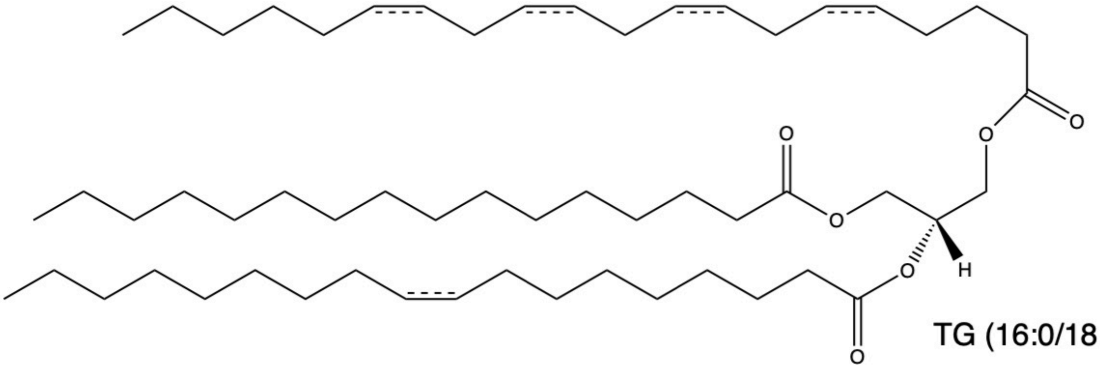

TG (16:0/18:1/20:4)

TG 16:0_18:1_22:5 as $[M+NH_4]^+$

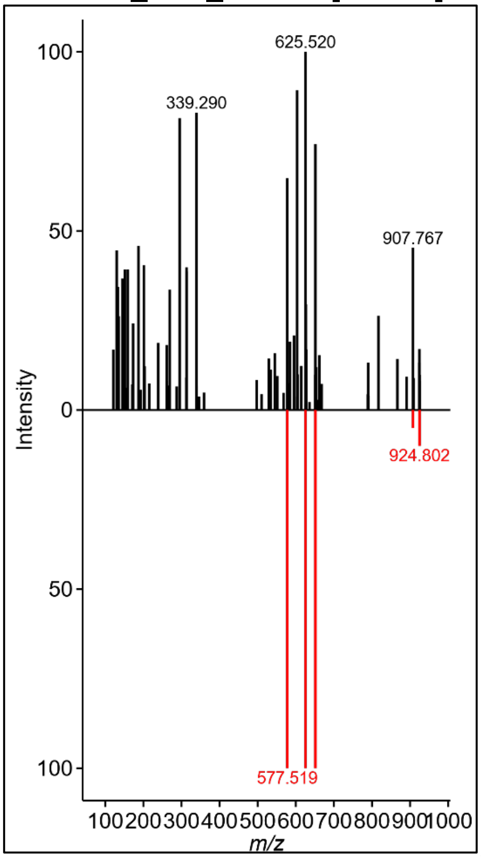

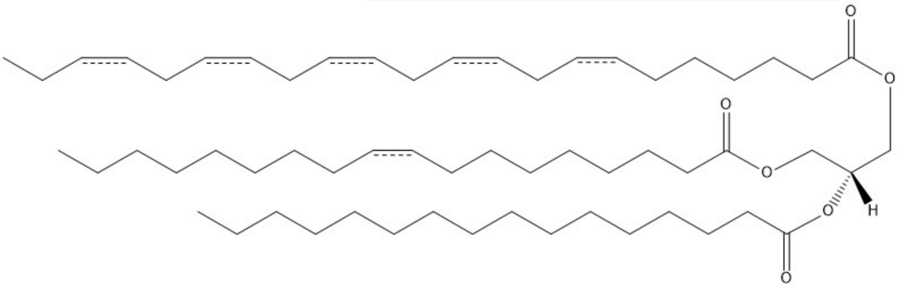

TG (16:0/18:1/22:5)

TG 18:0_18:1_22:5 as [M+NH$_4$]$^+$

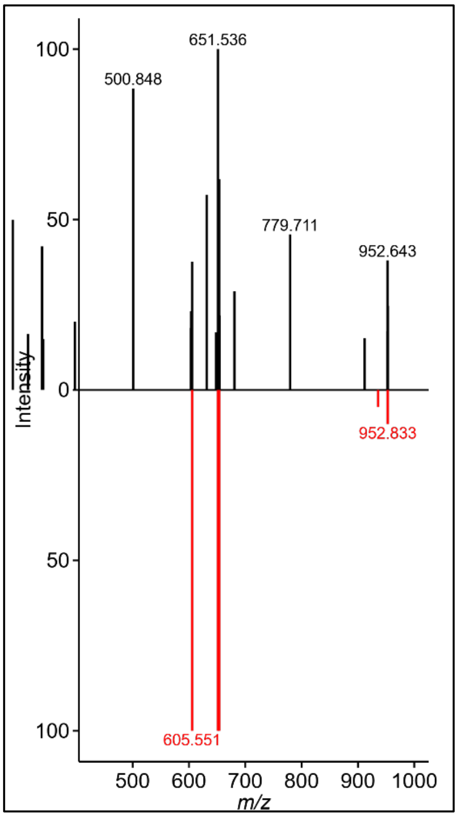

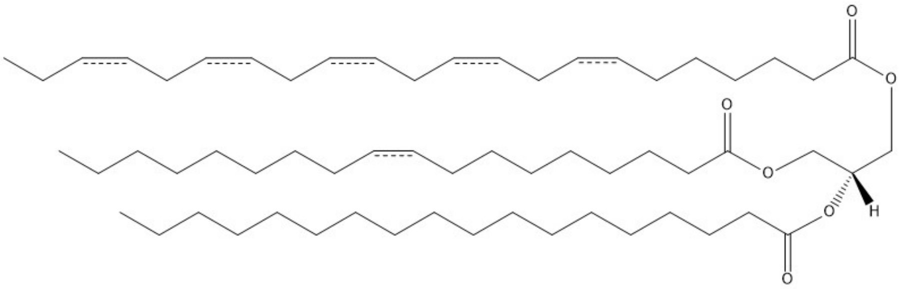

TG (18:0/18:1/22:5)

CHMP2A (Fig. 4h)

PC 18:2_18:2 as [M+OAc]⁻

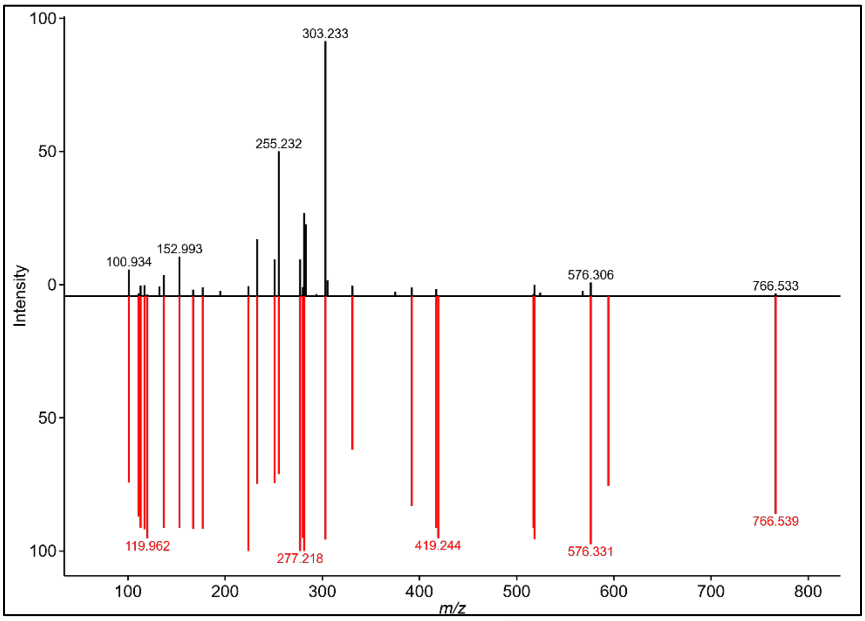

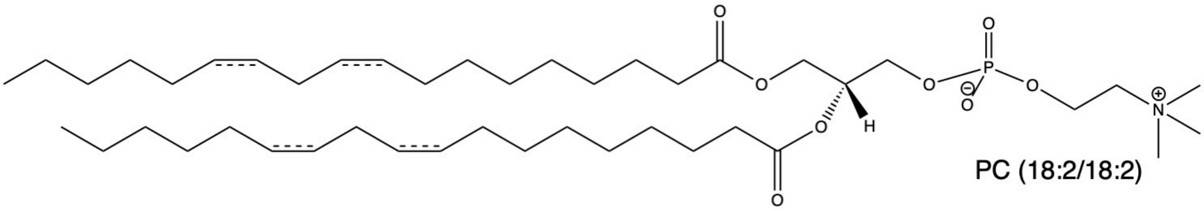

PC (18:2/18:2)

TG 16:0_18:1_22:5 as $[M+NH_4]^+$

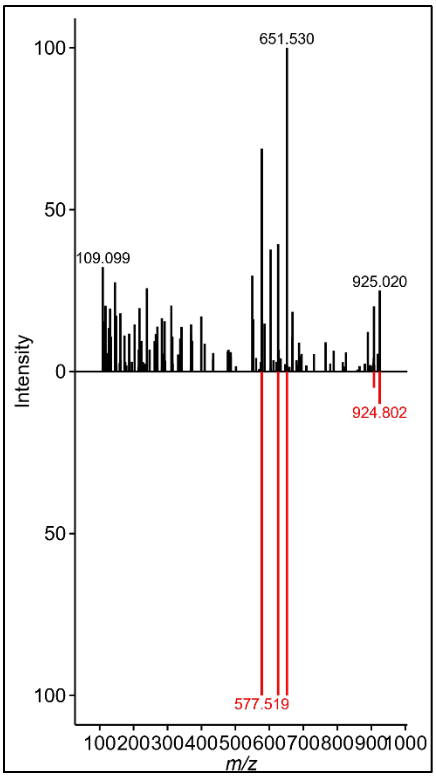

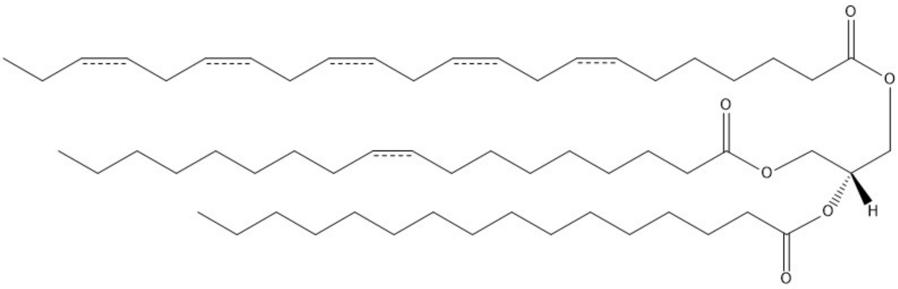

TG (16:0/18:1/22:5)

TG 18:1_18:1_22:5 as $[M+NH_4]^+$

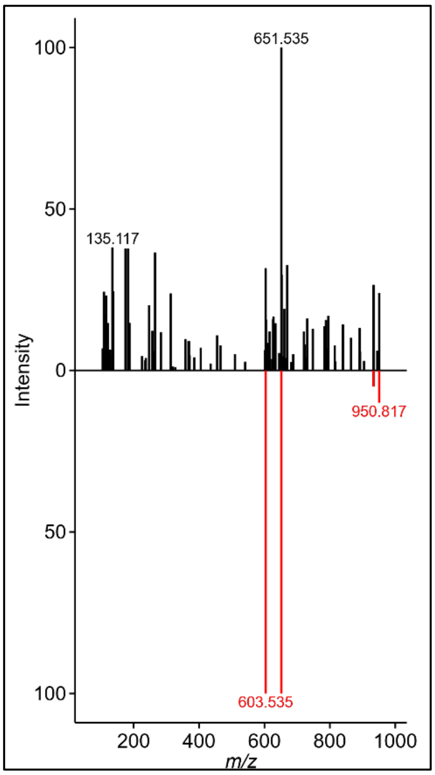

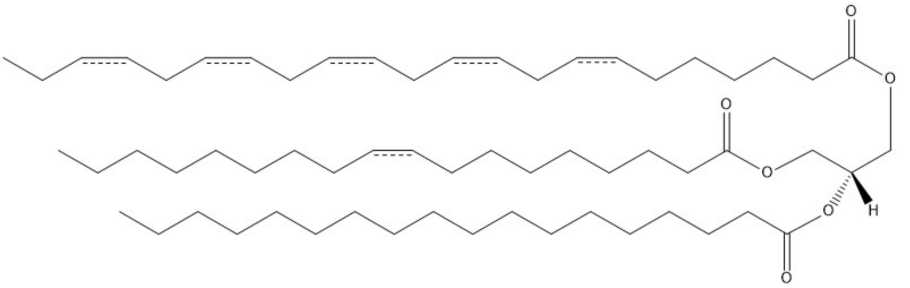

TG (18:0/18:1/22:5)

Supplementary Notes 2

Lipidomics reports. This appendix includes reports detailing *the overall study design, lipid extraction, analytical platform, quality control, method quantification and validation, and the lipid class description*.

Contents of Report

Created by https://lipidomicstandards.org, version v2.5.0

Separation Workflow 2

Overall study design 2
Lipid extraction 2
Analytical platform 2
Quality control 2
Method qualification and validation 2
Reporting 3

Sample Descriptions 3

Supplementary whole lipidome HeLa samples / Human / Cells 3

Lipid Class Descriptions 3

1) FA[M-H]- / Lipid identification 3
1) FA[M-H]- / Lipid quantification 3
2) Cer[M+CH3COO]- / Lipid identification 4
2) Cer[M+CH3COO]- / Lipid quantification 4
3) CE[M+NH4]+ / Lipid identification 4
3) CE[M+NH4]+ / Lipid quantification 5
4) CL[M-H]- / Lipid identification 5
4) CL[M-H]- / Lipid quantification 5
5) DG[M+Na]+ / Lipid identification 5
5) DG[M+Na]+ / Lipid quantification 6
6) HexCer[M-H]- / Lipid identification 6
6) HexCer[M-H]- / Lipid quantification 6
7) Hex2Cer[M+CH3COO]- / Lipid identification 7
7) Hex2Cer[M+CH3COO]- / Lipid quantification 7
8) Hex3Cer[M+CH3COO]- / Lipid identification 7
8) Hex3Cer[M+CH3COO]- / Lipid quantification 7
9) TG[M+NH4]+ / Lipid identification 8
9) TG[M+NH4]+ / Lipid quantification 8
10) SM[M+H]+ / Lipid identification 8
10) SM[M+H]+ / Lipid quantification 9
11) PS[M-H]- / Lipid identification 9
11) PS[M-H]- / Lipid quantification 9
12) PC[M+H]+ / Lipid identification 10
12) PC[M+H]+ / Lipid quantification 10
13) PC O[M+H]+ / Lipid identification 10
13) PC O[M+H]+ / Lipid quantification 11
14) LPC[M+H]+ / Lipid identification 11
14) LPC[M+H]+ / Lipid quantification 11
15) LPC O[M+H]+ / Lipid identification 12
15) LPC O[M+H]+ / Lipid quantification 12
16) PE[M+H]+ / Lipid identification 12
16) PE[M+H]+ / Lipid quantification 13
17) PI[M+NH4]+ / Lipid identification 13
17) PI[M+NH4]+ / Lipid quantification 13
18) PE[M-H]- / Lipid identification 13
18) PE[M-H]- / Lipid quantification 14
19) PE O[M-H]- / Lipid identification 14
19) PE O[M-H]- / Lipid quantification 14
20) PI[M-H]- / Lipid identification 15
20) PI[M-H]- / Lipid quantification 15
21) PG[M-H]- / Lipid identification 16
21) PG[M-H]- / Lipid quantification 16
22) PS[M+H]+ / Lipid identification 16
22) PS[M+H]+ / Lipid quantification 17
23) LPE[M-H]- / Lipid identification 17
23) LPE[M-H]- / Lipid quantification 17
24) LPE O[M-H]- / Lipid identification 17
24) LPE O[M-H]- / Lipid quantification 18
25) LPI[M-H]- / Lipid identification 18
25) LPI[M-H]- / Lipid quantification 18
26) LPS[M-H]- / Lipid identification 19

26) LPS[M-H]- / Lipid quantification 19
27) SM[M+CH3COO]- / Lipid identification 19
27) SM[M+CH3COO]- / Lipid quantification 19
28) PI O[M-H]- / Lipid identification 20
28) PI O[M-H]- / Lipid quantification 20
29) PS O[M-H]- / Lipid identification 20
29) PS O[M-H]- / Lipid quantification 21
30) TG O[M+Na]+ / Lipid identification 21
30) TG O[M+Na]+ / Lipid quantification 21
31) HexCer / Lipid identification 22
31) HexCer / Lipid quantification 22
32) Hex2Cer[M+H]+ / Lipid identification 22
32) Hex2Cer[M+H]+ / Lipid quantification 23
33) Hex3Cer[M+H]+ / Lipid identification 23
33) Hex3Cer[M+H]+ / Lipid quantification 23

Separation Workflow

Overall study design

Title of the study	Identification of specific lipid-protein interactions in dividing cells using lipid-trap mass spectrometry		
Document creation date	01/06/2026	Principal investigator	Ulrike Eggert
Institution	King's College London	Corresponding Email	ulrike.eggert@kcl.ac.uk
Is the workflow targeted or untargeted?	Untargeted	Clinical	No

Lipid extraction

Extraction method	2-phase system	pH adjustment	None
2-phase system	MTBE	Were internal standards added prior extraction?	Yes

Analytical platform

Ionization additives	Ammonium acetate, Ammonium formate, Formic acid	Number of separation dimensions	Two dimensions
Separation type 1	LC	Separation mode 1 (liquid)	RP
Separation window for lipid analyte selection (±) in minutes	0.3	Detector	Mass spectrometer
MS type	QTOF	MS vendor	Agilent
Ion source	ESI	MS Level	MS^2
Mass window for precursor ion isolation (in Da total isolation window)	1.3	Mass resolution for detected ion at MS^2	High resolution
Resolution at m/z 200 at MS^2	20000	Mass accuracy in ppm at MS^2	30
Recording mode of raw data at MS^2	Centroid mode	Was/Were additional dimension/techniques used	No

Quality control

Blanks	Yes	Type of Blanks	Extraction blank, Solvent blank, Internal standard blank

Quality control	Yes	Type of QC sample	Sample pool

Method qualification and validation

Method validation	Yes	Lipid recovery	Yes
Dynamic quantification range	No	Limit of quantitation (LOQ)/Limit of detection (LOD)	No

Precision	No	Accuracy	No
Guidelines followed	None		

Reporting

Are reported raw data uploaded into repository?	No	Are metadata available?	No
Raw data upload	Yes	Additional comments	Raw data is available in the supplementary data

Sample Descriptions

Supplementary whole lipidome HeLa samples / Human / Cells

Storage and collection conditions	Available	Provided preanalytical information	Time to freeze, Storage time (month), Freeze-thaw cycles, Preservation method
Temperature handling original sample	4-8 °C	Instant sample preparation	No
Time to freeze	between 10 and 20 minutes	Snap freezing in liquid N2	Yes
Storage temperature	-80 °C	Storage time (month)	0
Freeze-thaw cycles	0	Additives	None

Lipid Class Descriptions

1) FA[M-H]- / Lipid identification

Lipid class	FA	MS Level for identification	MS^2
Identification level	Species level	MS^2 adduct	[M-H]-
Fragments for identification			

Fragment name
$[FA-H]^-$

Isotope correction at MS^2	No	MS^2 verified by standard	No
Background check at MS^2	No	Did you presume assumptions for identification?	No
Limit of detection	No	RT verified by standard	No
Separation of isobaric/isomeric interferece confirmed	No	Model for separation prediction	No
Lipid Identification Software	MS-DIAL	Data manipulation	Centroiding
Nomenclature for intact lipid molecule	Yes	Nomenclature for fragment ions	N/A
Further identification remarks	MSDial was the main method of identification, however each assignment was checked using LipidMaps		

1) FA[M-H]- / Lipid quantification

Quantitative	No	Normalization to reference	No
Batch correction	No		

2) Cer[M+CH3COO]- / Lipid identification

Lipid class	Cer	MS Level for identification	MS^2
Identification level	Species level	MS^2 adduct	[M+CH3COO]-

Fragments for identification

Fragment name
-(+CH3COO)

Isotope correction at MS^2	No	MS^2 verified by standard	No
Background check at MS^2	No	Did you presume assumptions for identification?	No
Limit of detection	No	RT verified by standard	No
Separation of isobaric/isomeric interferece confirmed	No	Model for separation prediction	No

Lipid Identification Software	MS-DIAL	Data manipulation	Centroiding
Nomenclature for intact lipid molecule	Yes	Nomenclature for fragment ions	N/A

Further identification remarks	Lipids identified using MSDial were checked using LipidMaps		

2) Cer[M+CH3COO]- / Lipid quantification

Quantitative	No	Normalization to reference	No
Batch correction	No		

3) CE[M+NH4]+ / Lipid identification

Lipid class	CE	MS Level for identification	MS^2
Identification level	Species level	MS^2 adduct	[M+NH4]+

Fragments for identification

Fragment name
Cholesteryl fragment ion at m/z 369.3516

Isotope correction at MS^2	No	MS^2 verified by standard	Yes
Background check at MS^2	No	Did you presume assumptions for identification?	No
Limit of detection	No	RT verified by standard	Yes
Separation of isobaric/isomeric interferece confirmed	No	Model for separation prediction	No

Lipid Identification Software	MS-DIAL	Data manipulation	Centroiding
Nomenclature for intact lipid molecule	Yes	Nomenclature for fragment ions	N/A

Further identification remarks	Lipids identified by MSDial were checking using LipidMaps		

3) CE[M+NH4]+ / Lipid quantification

Quantitative	Yes	MS Level for quantification	MS^2

Internal lipid standard(s) MS^2

Internal standard	Fragment(s)	Endogenous subclass
18:1(d7) Chol Ester	Cholesteryl fragment ion at m/z 369.3516	Cholesterol esters

Type of quantification	Internal standard amount	Response correction	No
Type I isotope correction	No	Limit of quantification	No
Normalization to reference	No	Lipid Quantification Software	MS-DIAL
Batch correction	No		

4) CL[M-H]- / Lipid identification

Lipid class	CL	MS Level for identification	MS^2
Identification level	Species level	MS^2 adduct	[M-H]-

Fragments for identification

Fragment name
-FA1(-H) [-1]

Isotope correction at MS^2	No	MS^2 verified by standard	No
Background check at MS^2	No	Did you presume assumptions for identification?	No
Limit of detection	No	RT verified by standard	No
Separation of isobaric/isomeric interferece confirmed	No	Model for separation prediction	No
Lipid Identification Software	MS-DIAL	Data manipulation	Centroiding
Nomenclature for intact lipid molecule	Yes	Nomenclature for fragment ions	N/A
Further identification remarks	Lipids identified using MSDial were checked using LipidMaps		

4) CL[M-H]- / Lipid quantification

Quantitative	No	Normalization to reference	No
Batch correction	No		

5) DG[M+Na]+ / Lipid identification

Lipid class	DG	MS Level for identification	MS^2
Identification level	Species level	MS^2 adduct	[M+Na]+

Fragments for identification

Fragment name
[M-17]+ ion

Isotope correction at MS^2	No	MS^2 verified by standard	Yes
Background check at MS^2	No	Did you presume assumptions for	No

identification?

Limit of detection	No	RT verified by standard	Yes
Separation of isobaric/isomeric interferece confirmed	No	Model for separation prediction	No
Lipid Identification Software	MS-DIAL	Data manipulation	Centroiding
Nomenclature for intact lipid molecule	Yes	Nomenclature for fragment ions	N/A
Further identification remarks	Lipids identified by MSDial were checked using LipidMaps		

5) DG[M+Na]+ / Lipid quantification

Quantitative	Yes	MS Level for quantification	MS^2

Internal lipid standard(s) MS^2

Internal standard	Fragment(s)	Endogenous subclass
15:0-18:1(d7) DAG	Monoacylglycerol-type fragment	Diacylglycerides

Type of quantification	Internal standard amount	Response correction	No
Type I isotope correction	No	Limit of quantification	No
Normalization to reference	No	Lipid Quantification Software	MS-DIAL
Batch correction	No	Further quantification remarks	Lipids identified by MSDial were checked by LipidMaps

6) HexCer[M-H]- / Lipid identification

Lipid class	HexCer	MS Level for identification	MS^2
Identification level	Species level	MS^2 adduct	[M-H]-

Fragments for identification

Fragment name
-HG(Hex,162)

Isotope correction at MS^2	No	MS^2 verified by standard	No
Background check at MS^2	No	Did you presume assumptions for identification?	No
Limit of detection	No	RT verified by standard	No
Separation of isobaric/isomeric interferece confirmed	No	Model for separation prediction	No
Lipid Identification Software	MS-DIAL	Data manipulation	Centroiding
Nomenclature for intact lipid molecule	Yes	Nomenclature for fragment ions	N/A
Further identification remarks	Lipids identified by MSDial were checked using LipidMaps		

6) HexCer[M-H]- / Lipid quantification

Quantitative	No	Normalization to reference	No
Batch correction	No		

7) Hex2Cer[M+CH3COO]- / Lipid identification

Lipid class	Hex2Cer	MS Level for identification	MS^2
Identification level	Species level	MS^2 adduct	[M+CH3COO]-
Fragments for identification			

Fragment name
-(+CH3COO)
-HG(Hex,162)
-HG(Hex2,324)

Isotope correction at MS^2	No	MS^2 verified by standard	No
Background check at MS^2	No	Did you presume assumptions for identification?	No
Limit of detection	No	RT verified by standard	No
Separation of isobaric/isomeric interferece confirmed	No	Model for separation prediction	No
Lipid Identification Software	MS-DIAL	Data manipulation	Centroiding
Nomenclature for intact lipid molecule	Yes	Nomenclature for fragment ions	N/A
Further identification remarks	Lipids identified by MSDial were checked using LipidMaps		

7) Hex2Cer[M+CH3COO]- / Lipid quantification

Quantitative	No	Normalization to reference	No
Batch correction	No		

8) Hex3Cer[M+CH3COO]- / Lipid identification

Lipid class	Hex3Cer	MS Level for identification	MS^2
Identification level	Species level	MS^2 adduct	[M+CH3COO]-
Fragments for identification			

Fragment name
-(+CH3COO)
-HG(Hex,162)
-HG(Hex2,324)

Isotope correction at MS^2	No	MS^2 verified by standard	No
Background check at MS^2	No	Did you presume assumptions for identification?	No
Limit of detection	No	RT verified by standard	No
Separation of isobaric/isomeric interferece confirmed	No	Model for separation prediction	No
Lipid Identification Software	MS-DIAL	Data manipulation	Centroiding
Nomenclature for intact lipid molecule	Yes	Nomenclature for fragment ions	N/A
Further identification remarks	Lipids identified by MSDial were checked using LipidMaps		

8) Hex3Cer[M+CH3COO]- / Lipid quantification

Quantitative	No	Normalization to reference	No
Batch correction	No		

9) TG[M+NH4]+ / Lipid identification

Lipid class	TG	MS Level for identification	MS^2
Identification level	Species level	MS^2 adduct	[M+NH4]+
Fragments for identification			

Fragment name
FA1

Isotope correction at MS^2	No	MS^2 verified by standard	Yes
Background check at MS^2	No	Did you presume assumptions for identification?	No
Limit of detection	No	RT verified by standard	Yes
Separation of isobaric/isomeric interferece confirmed	No	Model for separation prediction	No
Lipid Identification Software	MS-DIAL	Data manipulation	Centroiding
Nomenclature for intact lipid molecule	Yes	Nomenclature for fragment ions	N/A
Further identification remarks	Lipids identified by MSDial were checked using LipidMaps		

9) TG[M+NH4]+ / Lipid quantification

Quantitative	Yes	MS Level for quantification	MS^2
Internal lipid standard(s) MS^2			

Internal standard	Fragment(s)	Endogenous subclass
15:0-18:1(d7)-15:0 TAG	FA1	Triacylglycerides

Type of quantification	Internal standard amount	Response correction	No
Type I isotope correction	No	Limit of quantification	No
Normalization to reference	No	Lipid Quantification Software	MS-DIAL
Batch correction	No	Further quantification remarks	Lipids identified by MSDial were checked by LipidMaps

10) SM[M+H]+ / Lipid identification

Lipid class	SM	MS Level for identification	MS^2
Identification level	Species level	MS^2 adduct	[M+H]+
Fragments for identification			

Fragment name
HG(PC,184)

Isotope correction at MS^2	No	MS^2 verified by standard	Yes
Background check at MS^2	No	Did you presume assumptions for identification?	No

Limit of detection	No	RT verified by standard	Yes

Separation of isobaric/isomeric interferece confirmed	No	Model for separation prediction	No
Lipid Identification Software	MS-DIAL	Data manipulation	Centroiding
Nomenclature for intact lipid molecule	Yes	Nomenclature for fragment ions	N/A
Further identification remarks	Lipids identified by MSDial were checked using LipidMaps		

10) SM[M+H]+ / Lipid quantification

Quantitative	Yes	MS Level for quantification	MS^2

Internal lipid standard(s) MS^2

Internal standard	Fragment(s)	Endogenous subclass
d18:1-18:1(d9) SM	HG(PC,184)	Sphingomyelins

Type of quantification	Internal standard amount	Response correction	No
Type I isotope correction	No	Limit of quantification	No
Normalization to reference	No	Lipid Quantification Software	MS-DIAL
Batch correction	No		

11) PS[M-H]- / Lipid identification

Lipid class	PS	MS Level for identification	MS^2
Identification level	Species level	MS^2 adduct	[M-H]-

Fragments for identification

Fragment name
-(C3H5NO2,87)

Isotope correction at MS^2	No	MS^2 verified by standard	Yes
Background check at MS^2	No	Did you presume assumptions for identification?	No
Limit of detection	No	RT verified by standard	Yes
Separation of isobaric/isomeric interferece confirmed	No	Model for separation prediction	No
Lipid Identification Software	MS-DIAL	Data manipulation	Centroiding
Nomenclature for intact lipid molecule	Yes	Nomenclature for fragment ions	N/A
Further identification remarks	Lipids identified by MSDial were checked using LipidMaps		

11) PS[M-H]- / Lipid quantification

Quantitative	Yes	MS Level for quantification	MS^2

Internal lipid standard(s) MS^2

Internal standard	Fragment(s)	Endogenous subclass
15:0-18:1(d7) PS	-HG(PS,185)	Phosphatidylserines

Type of quantification	Internal standard amount	Response correction	No

Type I isotope correction	No	Limit of quantification	No
Normalization to reference	No	Lipid Quantification Software	MS-DIAL
Batch correction	No		

12) PC[M+H]+ / Lipid identification

Lipid class	PC	MS Level for identification	MS^2
Identification level	Species level	MS^2 adduct	[M+H]+

Fragments for identification

Fragment name
HG(PC,184)

Isotope correction at MS^2	No	MS^2 verified by standard	Yes
Background check at MS^2	No	Did you presume assumptions for identification?	No
Limit of detection	No	RT verified by standard	Yes
Separation of isobaric/isomeric interferece confirmed	No	Model for separation prediction	No

Lipid Identification Software	MS-DIAL	Data manipulation	Centroiding
Nomenclature for intact lipid molecule	Yes	Nomenclature for fragment ions	N/A

Further identification remarks	Lipids identified by MSDial were checked using LipidMaps

12) PC[M+H]+ / Lipid quantification

Quantitative	Yes	MS Level for quantification	MS^2

Internal lipid standard(s) MS^2

Internal standard	Fragment(s)	Endogenous subclass
15:0-18:1(d7) PC	HG(PC,184)	Phosphatidylcholines

Type of quantification	Internal standard amount	Response correction	No
Type I isotope correction	No	Limit of quantification	No
Normalization to reference	No	Lipid Quantification Software	MS-DIAL
Batch correction	No		

13) PC O[M+H]+ / Lipid identification

Lipid class	PC O	MS Level for identification	MS^2
Identification level	Molecular species level	MS^2 adduct	[M+H]+

Fragments for identification

Fragment name
HG(PC,184)

Isotope correction at MS^2	No	MS^2 verified by standard	Yes
Background check at MS^2	No	Did you presume assumptions for identification?	No
Limit of detection	No	RT verified by standard	Yes
Separation of isobaric/isomeric	interferece confirmed		

	No	Model for separation prediction	No
Lipid Identification Software	MS-DIAL	Data manipulation	Centroiding

Nomenclature for intact lipid molecule	Yes	Nomenclature for fragment ions	N/A
Further identification remarks	Lipids identified by MSDial were checked using LipidMaps		

13) PC O[M+H]+ / Lipid quantification

Quantitative	Yes	MS Level for quantification	MS^2

Internal lipid standard(s) MS^2

Internal standard	Fragment(s)	Endogenous subclass
15:0-18:1(d7) PC	HG(PC,184)	Ether phosphatidylcholines

Type of quantification	Internal standard amount	Response correction	No
Type I isotope correction	No	Limit of quantification	No
Normalization to reference	No	Lipid Quantification Software	MS-DIAL
Batch correction	No	Further quantification remarks	PC Os were quantified using a PC internal standard. Because ether- and ester-linked PCs exhibit different ionisation efficiencies, PC O abundances should be interpreted as semi-quantitative.

14) LPC[M+H]+ / Lipid identification

Lipid class	LPC	MS Level for identification	MS^2
Identification level	Species level	MS^2 adduct	[M+H]+

Fragments for identification

Fragment name
HG(PC,184)

Isotope correction at MS^2	No	MS^2 verified by standard	Yes
Background check at MS^2	No	Did you presume assumptions for identification?	No
Limit of detection	No	RT verified by standard	Yes
Separation of isobaric/isomeric interferece confirmed	No	Model for separation prediction	No
Lipid Identification Software	MS-DIAL	Data manipulation	Centroiding
Nomenclature for intact lipid molecule	Yes	Nomenclature for fragment ions	N/A
Further identification remarks	Lipids identified by MSDial were checked using LipidMaps		

14) LPC[M+H]+ / Lipid quantification

Quantitative	Yes	MS Level for quantification	MS^2

Internal lipid standard(s) MS^2

Internal standard	Fragment(s)	Endogenous subclass
18:1(d7) Lyso PC	HG(PC,184)	Lysophosphatidylcholines

Type of quantification	Internal standard amount	Response correction	No
Type I isotope correction	No	Limit of quantification	No

Normalization to reference	No	Lipid Quantification Software	MS-DIAL
Batch correction	No		

15) LPC O[M+H]+ / Lipid identification

Lipid class	LPC O	MS Level for identification	MS^2
Identification level	Species level	MS^2 adduct	[M+H]+

Fragments for identification

Fragment name
HG(PC,184)

Isotope correction at MS^2	No	MS^2 verified by standard	Yes
Background check at MS^2	No	Did you presume assumptions for identification?	No
Limit of detection	No	RT verified by standard	Yes
Separation of isobaric/isomeric interferece confirmed	No	Model for separation prediction	No
Lipid Identification Software	MS-DIAL	Data manipulation	Centroiding
Nomenclature for intact lipid molecule	Yes	Nomenclature for fragment ions	N/A
Further identification remarks	Lipids identified by MSDial were checked using LipidMaps		

15) LPC O[M+H]+ / Lipid quantification

Quantitative	Yes	MS Level for quantification	MS^2

Internal lipid standard(s) MS^2

Internal standard	Fragment(s)	Endogenous subclass
18:1(d7) Lyso PC	HG(PC,184)	Lysophosphatidylcholines

Type of quantification	Internal standard amount	Response correction	No
Type I isotope correction	No	Limit of quantification	No
Normalization to reference	No	Lipid Quantification Software	MS-DIAL
Batch correction	No	Further quantification remarks	LPC Os were quantified using an LPC internal standard. Because ether- and ester-linked LPCs exhibit different ionisation efficiencies, LPC O abundances should be interpreted as semi-quantitative.

16) PE[M+H]+ / Lipid identification

Lipid class	PE	MS Level for identification	MS^2
Identification level	Species level	MS^2 adduct	[M+H]+

Fragments for identification

Fragment name

Fragment name
-HG(PE,141)

Isotope correction at MS^2	No	MS^2 verified by standard	No
Background check at MS^2	No	Did you presume assumptions for identification?	No
Limit of detection	No	RT verified by standard	Yes
Separation of isobaric/isomeric interferece confirmed	No	Model for separation prediction	No

Lipid Identification Software	MS-DIAL	Data manipulation	Centroiding
Nomenclature for intact lipid molecule	Yes	Nomenclature for fragment ions	N/A
Further identification remarks	Lipids identified by MSDial were checked using LipidMaps		

16) PE[M+H]+ / Lipid quantification

Quantitative	No	Normalization to reference	No
Batch correction	No		

17) PI[M+NH4]+ / Lipid identification

Lipid class	PI	MS Level for identification	MS^2
Identification level	Species level	MS^2 adduct	[M+NH4]+

Fragments for identification

Fragment name
-HG(PI,260+NH4)

Isotope correction at MS^2	No	MS^2 verified by standard	No
Background check at MS^2	No	Did you presume assumptions for identification?	No
Limit of detection	No	RT verified by standard	Yes
Separation of isobaric/isomeric interferece confirmed	No	Model for separation prediction	No

Lipid Identification Software	MS-DIAL	Data manipulation	Centroiding
Nomenclature for intact lipid molecule	Yes	Nomenclature for fragment ions	N/A
Further identification remarks	Lipids identified by MSDial were checked using LipidMaps		

17) PI[M+NH4]+ / Lipid quantification

Quantitative	No	Normalization to reference	No
Batch correction	No		

18) PE[M-H]- / Lipid identification

Lipid class	PE	MS Level for identification	MS^2
Identification level	Species level	MS^2 adduct	[M-H]-

Fragments for identification

Fragment name
HG(PE,196)

Isotope correction at MS^2	No	MS^2 verified by standard	Yes
Background check at MS^2	No	Did you presume assumptions for identification?	No
Limit of detection	No	RT verified by standard	Yes
Separation of isobaric/isomeric interferece confirmed	No	Model for separation prediction	No

Lipid Identification Software	MS-DIAL	Data manipulation	Centroiding
Nomenclature for intact lipid molecule	Yes	Nomenclature for fragment ions	N/A

Further identification remarks	Lipids identified by MSDial were checked using LipidMaps

18) PE[M-H]- / Lipid quantification

Quantitative	Yes	MS Level for quantification	MS^2

Internal lipid standard(s) MS^2

Internal standard	Fragment(s)	Endogenous subclass
15:0-18:1(d7) PE	HG(PE,196)	Phosphatidylethanolamines

Type of quantification	Internal standard amount	Response correction	No
Type I isotope correction	No	Limit of quantification	No
Normalization to reference	No	Lipid Quantification Software	MS-DIAL
Batch correction	No		

19) PE O[M-H]- / Lipid identification

Lipid class	PE O	MS Level for identification	MS^2
Identification level	Species level	MS^2 adduct	[M-H]-

Fragments for identification

Fragment name
HG(PE,196)

Isotope correction at MS^2	No	MS^2 verified by standard	Yes
Background check at MS^2	No	Did you presume assumptions for identification?	No
Limit of detection	No	RT verified by standard	Yes
Separation of isobaric/isomeric interferece confirmed	No	Model for separation prediction	No

Lipid Identification Software	MS-DIAL	Data manipulation	Centroiding
Nomenclature for intact lipid molecule	Yes	Nomenclature for fragment ions	N/A

Further identification remarks	Lipids identified by MSDial were checked using LipidMaps

19) PE O[M-H]- / Lipid quantification

Quantitative	Yes	MS Level for quantification	MS^2

Internal lipid standard(s) MS^2

Internal standard	Fragment(s)	Endogenous subclass
15:0-18:1(d7) PE	HG(PE,196)	Phosphatidylethanolamines

Type of quantification	Internal standard amount	Response correction	No
Type I isotope correction	No	Limit of quantification	No
Normalization to reference	No	Lipid Quantification Software	MS-DIAL
Batch correction	No	Further quantification remarks	PE Os were quantified using a PE internal standard. Because ether- and ester-linked PEs exhibit different ionisation efficiencies, PE O abundances should be interpreted as semi-quantitative.

20) PI[M-H]- / Lipid identification

Lipid class	PI	MS Level for identification	MS^2
Identification level	Species level	MS^2 adduct	[M-H]-

Fragments for identification

Fragment name
HG(PI,241)

Isotope correction at MS^2	No	MS^2 verified by standard	Yes
Background check at MS^2	No	Did you presume assumptions for identification?	No
Limit of detection	No	RT verified by standard	Yes
Separation of isobaric/isomeric interferece confirmed	No	Model for separation prediction	No
Lipid Identification Software	MS-DIAL	Data manipulation	Centroiding
Nomenclature for intact lipid molecule	Yes	Nomenclature for fragment ions	N/A
Further identification remarks	Lipids identified by MSDial were checked using LipidMaps		

20) PI[M-H]- / Lipid quantification

Quantitative	Yes	MS Level for quantification	MS^2

Internal lipid standard(s) MS^2

Internal standard	Fragment(s)	Endogenous subclass
15:0-18:1(d7) PI	HG(PI,241)	Phosphatidylinositols

Type of quantification	Internal standard amount	Response correction	No
Type I isotope correction	No	Limit of quantification	No
Normalization to reference	No	Lipid Quantification Software	MS-DIAL
Batch correction	No		

21) PG[M-H]- / Lipid identification

Lipid class	PG	MS Level for identification	MS^2
Identification level	Species level	MS^2 adduct	[M-H]-

Fragments for identification

Fragment name
HG(PG,227)

Isotope correction at MS^2	No	MS^2 verified by standard	Yes
Background check at MS^2	No	Did you presume assumptions for identification?	No
Limit of detection	No	RT verified by standard	Yes
Separation of isobaric/isomeric interferece confirmed	No	Model for separation prediction	No

Lipid Identification Software	MS-DIAL	Data manipulation	Centroiding
Nomenclature for intact lipid molecule	Yes	Nomenclature for fragment ions	N/A

Further identification remarks	Lipids identified by MSDial were checked using LipidMaps		

21) PG[M-H]- / Lipid quantification

Quantitative	Yes	MS Level for quantification	MS^2

Internal lipid standard(s) MS^2

Internal standard	Fragment(s)	Endogenous subclass
15:0-18:1(d7) PG	HG(PG,227)	Phosphatidylglycerols

Type of quantification	Internal standard amount	Response correction	No
Type I isotope correction	No	Limit of quantification	No
Normalization to reference	No	Lipid Quantification Software	MS-DIAL
Batch correction	No		

22) PS[M+H]+ / Lipid identification

Lipid class	PS	MS Level for identification	MS^2
Identification level	Species level	MS^2 adduct	[M+H]+

Fragments for identification

Fragment name
-HG(PS,185)

Isotope correction at MS^2	No	MS^2 verified by standard	No
Background check at MS^2	No	Did you presume assumptions for identification?	No
Limit of detection	No	RT verified by standard	Yes
Separation of isobaric/isomeric interferece confirmed	No	Model for separation prediction	No

Lipid Identification Software	MS-DIAL	Data manipulation	Centroiding
Nomenclature for intact lipid molecule	Yes	Nomenclature for fragment ions	N/A

Further identification remarks	Lipids identified by MSDial		

were checked using LipidMaps

22) PS[M+H]+ / Lipid quantification

Quantitative	No	Normalization to reference	No
Batch correction	No		

23) LPE[M-H]- / Lipid identification

Lipid class	LPE	MS Level for identification	MS^2
Identification level	Species level	MS^2 adduct	[M-H]-

Fragments for identification

Fragment name
GP(153)

Isotope correction at MS^2	No	MS^2 verified by standard	Yes
Background check at MS^2	No	Did you presume assumptions for identification?	No
Limit of detection	No	RT verified by standard	Yes
Separation of isobaric/isomeric interferece confirmed	No	Model for separation prediction	No
Lipid Identification Software	MS-DIAL	Data manipulation	Centroiding
Nomenclature for intact lipid molecule	Yes	Nomenclature for fragment ions	N/A
Further identification remarks	Lipids identified by MSDial were checked using LipidMaps		

23) LPE[M-H]- / Lipid quantification

Quantitative	Yes	MS Level for quantification	MS^2

Internal lipid standard(s) MS^2

Internal standard	Fragment(s)	Endogenous subclass
18:1(d7) Lyso PE	GP(153)	Lyso-phosphatidylethanolamine

Type of quantification	Internal standard amount	Response correction	No
Type I isotope correction	No	Limit of quantification	No
Normalization to reference	No	Lipid Quantification Software	MS-DIAL
Batch correction	No		

24) LPE O[M-H]- / Lipid identification

Lipid class	LPE O	MS Level for identification	MS^2
Identification level	Species level	MS^2 adduct	[M-H]-

Fragments for identification

Fragment name
GP(153)

Isotope correction at MS^2	No	MS^2 verified by standard	Yes
Background check at MS^2	No	Did you presume assumptions for	No

identification?

Limit of detection	No	RT verified by standard	Yes
Separation of isobaric/isomeric interferece confirmed	No	Model for separation prediction	No
Lipid Identification Software	MS-DIAL	Data manipulation	Centroiding
Nomenclature for intact lipid molecule	Yes	Nomenclature for fragment ions	N/A
Further identification remarks	Lipids identified by MSDial were checked using LipidMaps		

24) LPE O[M-H]- / Lipid quantification

Quantitative	Yes	MS Level for quantification	MS^2

Internal lipid standard(s) MS^2

Internal standard	Fragment(s)	Endogenous subclass
18:1(d7) Lyso PE	GP(153)	Lyso-phosphatidylethanolamine

Type of quantification	Internal standard amount	Response correction	No
Type I isotope correction	No	Limit of quantification	No
Normalization to reference	No	Lipid Quantification Software	MS-DIAL
Batch correction	No	Further quantification remarks	LPE Os were quantified using an LPE internal standard. Because ether- and ester-linked LPEs exhibit different ionisation efficiencies, LPE O abundances should be interpreted as semi-quantitative.

25) LPI[M-H]- / Lipid identification

Lipid class	LPI	MS Level for identification	MS^2
Identification level	Species level	MS^2 adduct	[M-H]-

Fragments for identification

Fragment name
GP(153)
HG(PI,241)

Isotope correction at MS^2	No	MS^2 verified by standard	No
Background check at MS^2	No	Did you presume assumptions for identification?	No
Limit of detection	No	RT verified by standard	Yes
Separation of isobaric/isomeric interferece confirmed	No	Model for separation prediction	No
Lipid Identification Software	MS-DIAL	Data manipulation	Centroiding
Nomenclature for intact lipid molecule	Yes	Nomenclature for fragment ions	N/A
Further identification remarks	Lipids identified by MSDial were checked using LipidMaps		

25) LPI[M-H]- / Lipid quantification

Quantitative	No	Normalization to reference	No
Batch correction	No		

26) LPS[M-H]- / Lipid identification

Lipid class	LPS	MS Level for identification	MS^2
Identification level	Species level	MS^2 adduct	[M-H]-
Fragments for identification			

Fragment name
GP(153)

Isotope correction at MS^2	No	MS^2 verified by standard	No
Background check at MS^2	No	Did you presume assumptions for identification?	No
Limit of detection	No	RT verified by standard	Yes
Separation of isobaric/isomeric interferece confirmed	No	Model for separation prediction	No
Lipid Identification Software	MS-DIAL	Data manipulation	Centroiding
Nomenclature for intact lipid molecule	Yes	Nomenclature for fragment ions	N/A
Further identification remarks	Lipids identified by MSDial were checked using LipidMaps		

26) LPS[M-H]- / Lipid quantification

Quantitative	No	Normalization to reference	No
Batch correction	No		

27) SM[M+CH3COO]- / Lipid identification

Lipid class	SM	MS Level for identification	MS^2
Identification level	Species level	MS^2 adduct	[M+CH3COO]-
Fragments for identification			

Fragment name
HG(PC,168)

Isotope correction at MS^2	No	MS^2 verified by standard	No
Background check at MS^2	No	Did you presume assumptions for identification?	No
Limit of detection	No	RT verified by standard	Yes
Separation of isobaric/isomeric interferece confirmed	No	Model for separation prediction	No
Lipid Identification Software	MS-DIAL	Data manipulation	Centroiding
Nomenclature for intact lipid molecule	Yes	Nomenclature for fragment ions	N/A
Further identification remarks	Lipids identified by MSDial were checked using LipidMaps		

27) SM[M+CH3COO]- / Lipid quantification

Quantitative	No	Normalization to reference	No
Batch correction	No		

28) PI O[M-H]- / Lipid identification

Lipid class	PI O	MS Level for identification	MS^2
Identification level	Species level	MS^2 adduct	[M-H]-
Fragments for identification			

Fragment name
HG(PI,241)

Isotope correction at MS^2	No	MS^2 verified by standard	Yes
Background check at MS^2	No	Did you presume assumptions for identification?	No
Limit of detection	No	RT verified by standard	Yes
Separation of isobaric/isomeric interferece confirmed	No	Model for separation prediction	No
Lipid Identification Software	MS-DIAL	Data manipulation	Centroiding
Nomenclature for intact lipid molecule	Yes	Nomenclature for fragment ions	N/A
Further identification remarks	Lipids identified by MSDial were checked using LipidMaps		

28) PI O[M-H]- / Lipid quantification

Quantitative	Yes	MS Level for quantification	MS^2
Internal lipid standard(s) MS^2			

Internal standard	Fragment(s)	Endogenous subclass
15:0-18:1(d7) PI	HG(PI,241)	Phosphatidylinositols

Type of quantification	Internal standard amount	Response correction	No
Type I isotope correction	No	Limit of quantification	No
Normalization to reference	No	Lipid Quantification Software	MS-DIAL
Batch correction	No	Further quantification remarks	PI Os were quantified using a PI internal standard. Because ether- and ester-linked PIs exhibit different ionisation efficiencies, PI O abundances should be interpreted as semi-quantitative.

29) PS O[M-H]- / Lipid identification

Lipid class	PS O	MS Level for identification	MS^2
Identification level	Species level	MS^2 adduct	[M-H]-
Fragments for identification			

Fragment name
-(C3H5NO2,87)

Isotope correction at MS^2	No	MS^2 verified by standard	Yes
Background check at MS^2	No	Did you presume assumptions for identification?	No
Limit of detection	No	RT verified by standard	Yes
Separation of isobaric/isomeric interferece confirmed	No	Model for separation prediction	No

Lipid Identification Software	MS-DIAL	Data manipulation	Centroiding
Nomenclature for intact lipid molecule	Yes	Nomenclature for fragment ions	N/A

Further identification remarks	Lipids identified by MSDial were checked using LipidMaps

29) PS O[M-H]- / Lipid quantification

Quantitative	Yes	MS Level for quantification	MS^2

Internal lipid standard(s) MS^2

Internal standard	Fragment(s)	Endogenous subclass
15:0-18:1(d7) PS	-(C3H5NO2,87)	Phosphatidylserines

Type of quantification	Internal standard amount	Response correction	No
Type I isotope correction	No	Limit of quantification	No
Normalization to reference	No	Lipid Quantification Software	MS-DIAL
Batch correction	No	Further quantification remarks	PS Os were quantified using a PS internal standard. Because ether- and ester-linked PSs exhibit different ionisation efficiencies, PS O abundances should be interpreted as semi-quantitative.

30) TG O[M+Na]+ / Lipid identification

Lipid class	TG O	MS Level for identification	MS^2
Identification level	Species level	MS^2 adduct	[M+Na]+

Fragments for identification

Fragment name
FA1

Isotope correction at MS^2	No	MS^2 verified by standard	Yes
Background check at MS^2	No	Did you presume assumptions for identification?	No
Limit of detection	No	RT verified by standard	Yes
Separation of isobaric/isomeric interferece confirmed	No	Model for separation prediction	No

Lipid Identification Software	MS-DIAL	Data manipulation	Centroiding
Nomenclature for intact lipid molecule	Yes	Nomenclature for fragment ions	N/A

Further identification remarks	Lipids identified by MSDial were checked using LipidMaps

30) TG O[M+Na]+ / Lipid quantification

Quantitative	Yes	MS Level for quantification	MS^2

Internal lipid standard(s) MS^2

Internal standard	Fragment(s)	Endogenous subclass
15:0-18:1(d7)-15:0 TAG	FA1	Triacylglycerides

Type of quantification	Internal standard amount	Response correction	No
Type I isotope correction	No	Limit of quantification	No
Normalization to reference	No	Lipid Quantification Software	MS-DIAL
Batch correction	No	Further quantification remarks	Ether triacylglycerols (TG-O) were quantified using a TAG internal standard. Because ether- and ester-linked TAGs exhibit different ionisation efficiencies, TG-O abundances should be interpreted as semi-quantitative.

31) HexCer / Lipid identification

Lipid class	HexCer	MS Level for identification	MS^2

Fragments for identification

Fragment name
-(H2O,18)
-HG(Hex,198)

Identification level	Species level	Isotope correction at MS^2	No
MS^2 verified by standard	No	Background check at MS^2	No
Did you presume assumptions for identification?	No	Limit of detection	No
RT verified by standard	No	Separation of isobaric/isomeric interferece confirmed	No
Model for separation prediction	No	Lipid Identification Software	MS-DIAL
Data manipulation	Centroiding	Nomenclature for intact lipid molecule	Yes
Nomenclature for fragment ions	N/A	Further identification remarks	Lipids identified by MSDial were checked using LipidMaps

31) HexCer / Lipid quantification

Quantitative	No	Normalization to reference	No
Batch correction	No		

32) Hex2Cer[M+H]+ / Lipid identification

Lipid class	Hex2Cer	MS Level for identification	MS^2
Identification level	Species level	MS^2 adduct	[M+H]+

Fragments for identification

Fragment name
-(H2O,18)

Fragment name
-HG(Hex2,342)

Isotope correction at MS^2	No	MS^2 verified by standard	No
Background check at MS^2	No	Did you presume assumptions for identification?	No
Limit of detection	No	RT verified by standard	No
Separation of isobaric/isomeric interferece confirmed	No	Model for separation prediction	No

Lipid Identification Software	MS-DIAL	Data manipulation	Centroiding
Nomenclature for intact lipid molecule	Yes	Nomenclature for fragment ions	N/A
Further identification remarks	Lipids identified by MSDial were checked using LipidMaps		

32) Hex2Cer[M+H]+ / Lipid quantification

Quantitative	No	Normalization to reference	No
Batch correction	No		

33) Hex3Cer[M+H]+ / Lipid identification

Lipid class	Hex3Cer	MS Level for identification	MS^2
Identification level	Species level	MS^2 adduct	[M+H]+

Fragments for identification

Fragment name
-(H2O,18)
-HG(Hex,180)
-HG(Hex2,342)
-HG(Hex3,504)

Isotope correction at MS^2	No	MS^2 verified by standard	No
Background check at MS^2	No	Did you presume assumptions for identification?	No
Limit of detection	No	RT verified by standard	No
Separation of isobaric/isomeric interferece confirmed	No	Model for separation prediction	No

Lipid Identification Software	MS-DIAL	Data manipulation	Centroiding
Nomenclature for intact lipid molecule	Yes	Nomenclature for fragment ions	N/A
Further identification remarks	Lipids identified by MSDial were checked using LipidMaps		

33) Hex3Cer[M+H]+ / Lipid quantification

Quantitative	No	Normalization to reference	No
Batch correction	No		
