# Supplementary material for: Lipid-trap mass spectrometry identifies lipid–protein interactions in cells
Source: Nat Cell Biol. 2026 Apr 13;28(5):1066–75. doi: 10.1038/s41556-026-01928-6 (PMC13179130; doi:10.1038/s41556-026-01928-6)
Supplement: Supplementary file 2 — Reporting Summary [file 41556_2026_1928_MOESM2_ESM.pdf]

nature portfolio

Corresponding author(s): Ulrike Eggert

Last updated by author(s): Feb 20, 2026

Reporting Summary

Nature Portfolio wishes to improve the reproducibility of the work that we publish. This form provides structure for consistency and transparency in reporting. For further information on Nature Portfolio policies, see our Editorial Policies and the Editorial Policy Checklist.

Statistics

For all statistical analyses, confirm that the following items are present in the figure legend, table legend, main text, or Methods section.

n/a	Confirmed	
☐	☒	The exact sample size (*n*) for each experimental group/condition, given as a discrete number and unit of measurement
☐	☒	A statement on whether measurements were taken from distinct samples or whether the same sample was measured repeatedly
☐	☒	The statistical test(s) used AND whether they are one- or two-sided<br>*Only common tests should be described solely by name; describe more complex techniques in the Methods section.*
☒	☐	A description of all covariates tested
☐	☒	A description of any assumptions or corrections, such as tests of normality and adjustment for multiple comparisons
☐	☒	A full description of the statistical parameters including central tendency (e.g. means) or other basic estimates (e.g. regression coefficient) AND variation (e.g. standard deviation) or associated estimates of uncertainty (e.g. confidence intervals)
☐	☒	For null hypothesis testing, the test statistic (e.g. *F*, *t*, *r*) with confidence intervals, effect sizes, degrees of freedom and *P* value noted<br>*Give P values as exact values whenever suitable.*
☒	☐	For Bayesian analysis, information on the choice of priors and Markov chain Monte Carlo settings
☒	☐	For hierarchical and complex designs, identification of the appropriate level for tests and full reporting of outcomes
☒	☐	Estimates of effect sizes (e.g. Cohen's *d*, Pearson's *r*), indicating how they were calculated

Our web collection on statistics for biologists contains articles on many of the points above.

Software and code

Policy information about availability of computer code

Data collection	Imaging data was acquired using NIS elements (Nikon). Mass spec data was acquired using MassHunter Workstation Software (version B.08.00 , Build 8.00.8058.3 SP1, Agilent Technologies).
Data analysis	MassHunter Profinder Software (version B.08.00 and B.10.00, Agilent Technologies) was used for mass spectrometry data processing. Mass Profiler Professional (MPP, Agilent Technologies) was used for statistical analysis. MassHunter Qualitative Analysis Software (version B.07.00, Agilent Technologies) was used to generate extracted ion chromatograms (EIC) for lipid species with known m/z and retention times, such as derivatized phosphoinositides.

For manuscripts utilizing custom algorithms or software that are central to the research but not yet described in published literature, software must be made available to editors and reviewers. We strongly encourage code deposition in a community repository (e.g. GitHub). See the Nature Portfolio guidelines for submitting code & software for further information.

Data

Policy information about availability of data

All manuscripts must include a data availability statement. This statement should provide the following information, where applicable:

- Accession codes, unique identifiers, or web links for publicly available datasets
- A description of any restrictions on data availability
- For clinical datasets or third party data, please ensure that the statement adheres to our policy

All data supporting this study are available within the paper and its Supplementary Information. This includes MS/MS data used to identify the lipid species reported in the manuscript. Should any further raw data be required in a different format, they are available from the corresponding author upon reasonable request. Plasmids used to generate stable cell lines are available from Addgene (#138479 and #12260). All materials are available upon request.

Research involving human participants, their data, or biological material

Policy information about studies with human participants or human data. See also policy information about sex, gender (identity/presentation), and sexual orientation and race, ethnicity and racism.

Reporting on sex and gender	N/A
Reporting on race, ethnicity, or other socially relevant groupings	N/A
Population characteristics	N/A
Recruitment	N/A
Ethics oversight	N/A

Note that full information on the approval of the study protocol must also be provided in the manuscript.

Field-specific reporting

Please select the one below that is the best fit for your research. If you are not sure, read the appropriate sections before making your selection.

☒ Life sciences ☐ Behavioural & social sciences ☐ Ecological, evolutionary & environmental sciences

For a reference copy of the document with all sections, see nature.com/documents/nr-reporting-summary-flat.pdf

Life sciences study design

All studies must disclose on these points even when the disclosure is negative.

Sample size	No statistical methods were used to predetermine sample size. Samples sizes are similar to those commonly used in the field and reported in previous publications (e.g. Donà et al, 2021, JACS).
Data exclusions	No data were excluded.
Replication	All experiments were performed in at least two biological replicates to ensure reproducibility.
Randomization	N/A
Blinding	N/A

Reporting for specific materials, systems and methods

We require information from authors about some types of materials, experimental systems and methods used in many studies. Here, indicate whether each material, system or method listed is relevant to your study. If you are not sure if a list item applies to your research, read the appropriate section before selecting a response.

Materials & experimental systems

n/a	Involved in the study
☐	☒ Antibodies
☐	☒ Eukaryotic cell lines
☒	☐ Palaeontology and archaeology
☒	☐ Animals and other organisms
☒	☐ Clinical data
☒	☐ Dual use research of concern
☒	☐ Plants

Methods

n/a	Involved in the study
☒	☐ ChIP-seq
☒	☐ Flow cytometry
☒	☐ MRI-based neuroimaging

Antibodies

Antibodies used

mouse anti-αTubulin (Thermo Fisher 236 10501, immunofluorescence microscopy 1:1000), mouse anti-RACGAP1 (Everest Biotech EB05315, western blot 1:1000), mouse anti-GAPDH (Proteintech10494-1-AP, western blot 1:10000), mouse anti-GFP (Abcam ab1218, western blot 1:5000), rabbit anti-GFP (Abcam ab6556, western blot 1:1000), rabbit anti-CHMP4B (Abcam ab76334, western blot 1:1000), rabbit and mouse horseradish peroxidase (HRP) conjugated secondary antibodies (Stratech-Jackson Immunoresearch, western blot 1:1000), Alexa 488 rabbit and mouse (Jackson ImmunoResearch AB_2340846 and Jackson ImmunoResearch AB_2338871, immunofluorescence microscopy 1:500), Alexa 594 rabbit and mouse (Jackson ImmunoResearchAB_2313584 and Jackson ImmunoResearch AB_2338046. immunofluorescence microscopy 1:500)

Validation

All of these antibodies are well established commercial antibodies that have been used in numerous prior publications.

Eukaryotic cell lines

Policy information about cell lines and Sex and Gender in Research

Cell line source(s)

HeLa cells were a gift from Jeremy Carlton's lab. HEK293GP and HEK293T cells were from Clontech (Takara Bio).

Authentication

HeLa cell line was authenticated by STR profiling (Eurofins MWG). All cell lines were authenticated based on morphology and growth condition.

Mycoplasma contamination

Cell lines were tested and confirmed negative for mycoplasma.

Commonly misidentified lines
(See ICLAC register)

None

Plants

Seed stocks

Report on the source of all seed stocks or other plant material used. If applicable, state the seed stock centre and catalogue number. If plant specimens were collected from the field, describe the collection location, date and sampling procedures.

Novel plant genotypes

Describe the methods by which all novel plant genotypes were produced. This includes those generated by transgenic approaches, gene editing, chemical/radiation-based mutagenesis and hybridization. For transgenic lines, describe the transformation method, the number of independent lines analyzed and the generation upon which experiments were performed. For gene-edited lines, describe the editor used, the endogenous sequence targeted for editing, the targeting guide RNA sequence (if applicable) and how the editor was applied.

Authentication

Describe any authentication procedures for each seed stock used or novel genotype generated. Describe any experiments used to assess the effect of a mutation and, where applicable, how potential secondary effects (e.g. second site T-DNA insertions, mosiacism, off-target gene editing) were examined.
