# Supplementary figures and images for: Lipid-trap mass spectrometry identifies lipid–protein interactions in cells
Source: Nat Cell Biol. 2026 Apr 13;28(5):1066–75. doi: 10.1038/s41556-026-01928-6 (PMC13179130; doi:10.1038/s41556-026-01928-6)

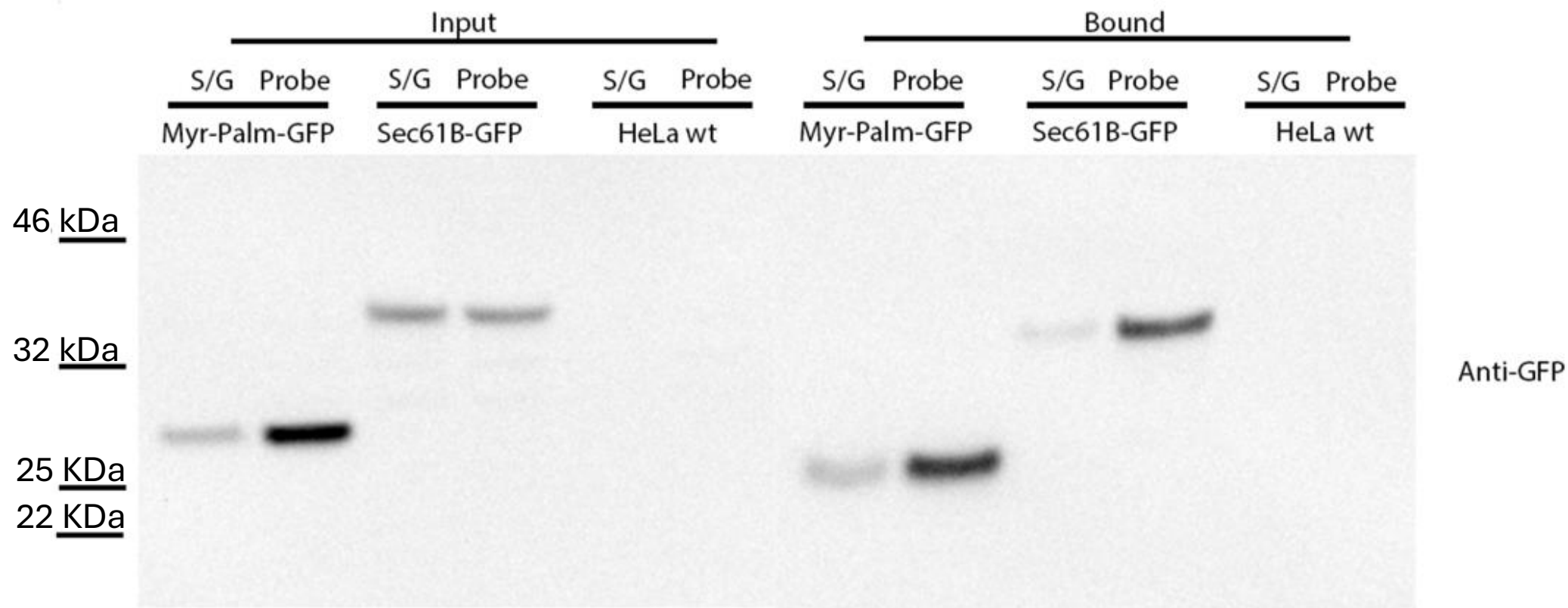

Supplement: Supplementary file 11 — Unprocessed blots. [file 41556_2026_1928_MOESM11_ESM.pdf]

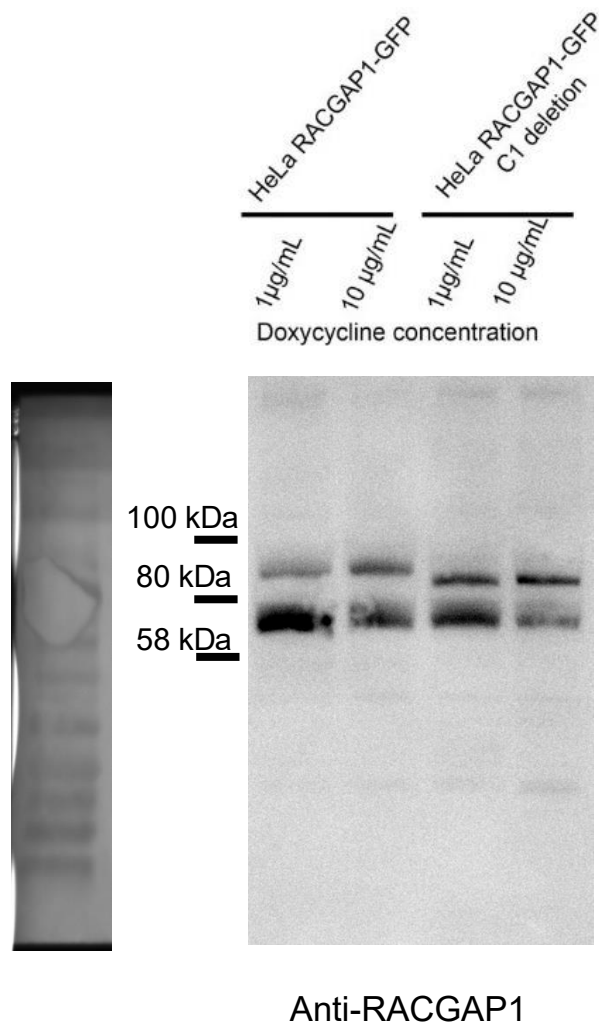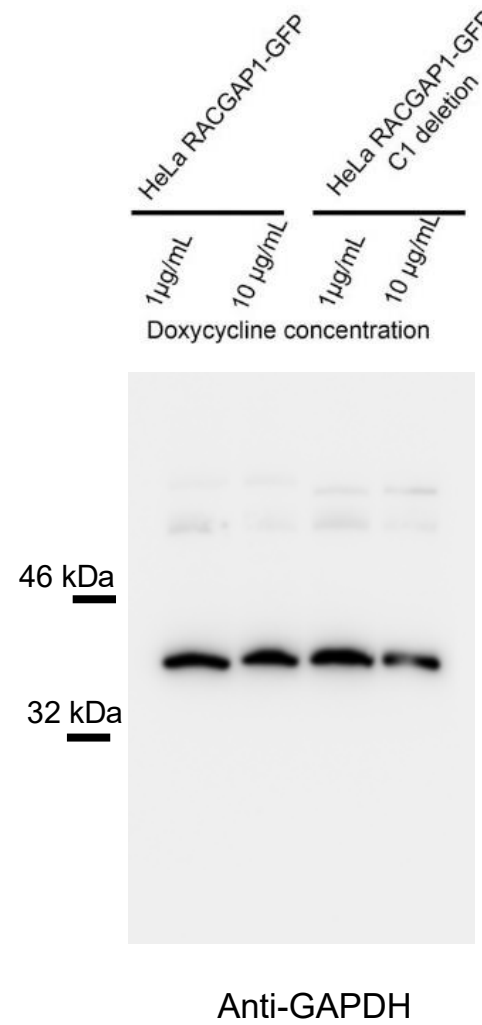

Supplement: Supplementary file 13 — Unprocessed blots. [file 41556_2026_1928_MOESM13_ESM.pdf]

**Fig. 6c**

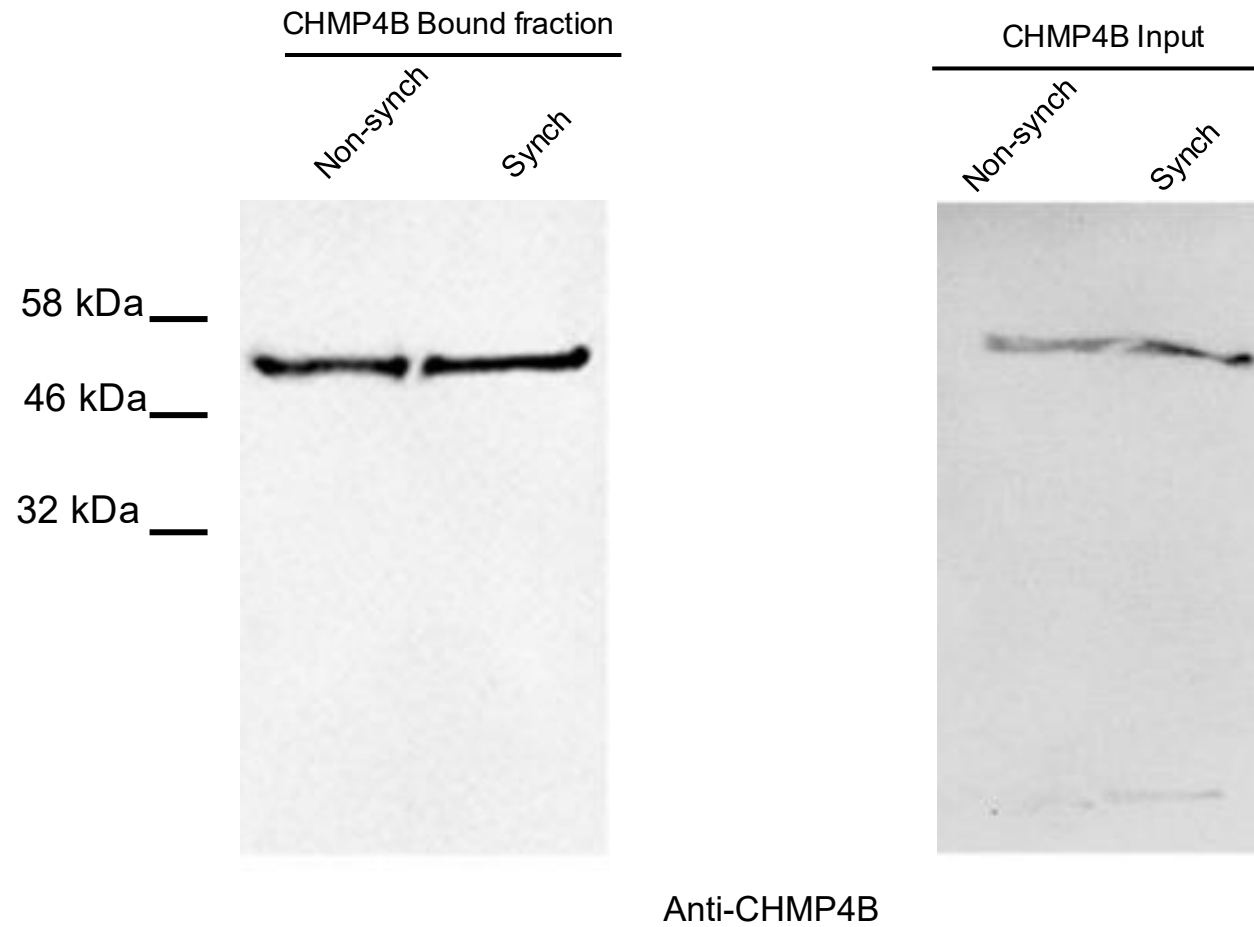

**Fig. 6e**

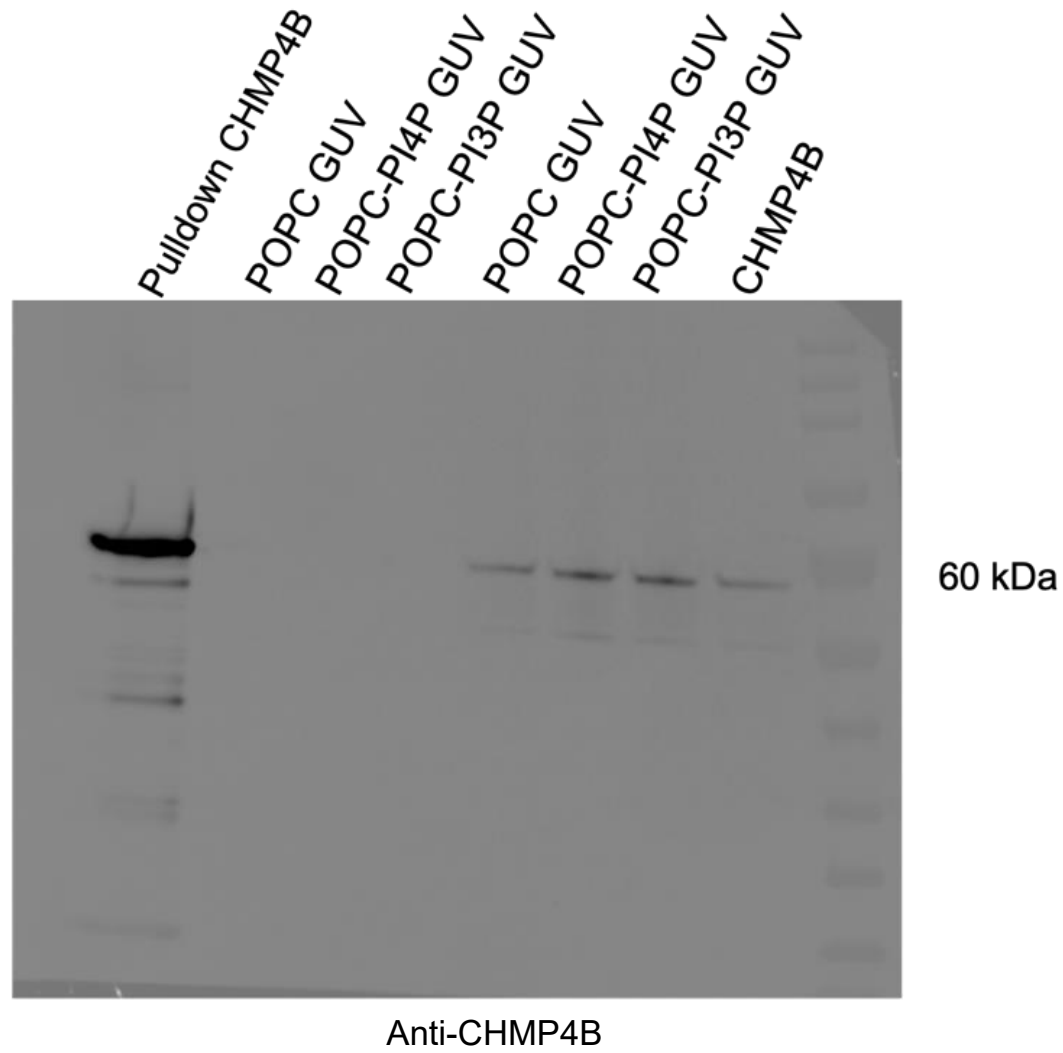

Supplement: Supplementary file 15 — Unprocessed blots. [file 41556_2026_1928_MOESM15_ESM.pdf]
